# Immune Cell Composition in Human Non-small Cell Lung Cancer

**DOI:** 10.3389/fimmu.2018.03101

**Published:** 2019-02-01

**Authors:** Branislava Stankovic, Heidi Anine Korsmo Bjørhovde, Renate Skarshaug, Henrik Aamodt, Astri Frafjord, Elisabeth Müller, Clara Hammarström, Kahsai Beraki, Espen S. Bækkevold, Per Reidar Woldbæk, Åslaug Helland, Odd Terje Brustugun, Inger Øynebråten, Alexandre Corthay

**Affiliations:** ^1^Tumor Immunology Lab, Department of Pathology, Rikshospitalet, Oslo University Hospital and University of Oslo, Oslo, Norway; ^2^Department of Cardiothoracic Surgery, Ullevål Hospital, Oslo University Hospital, Oslo, Norway; ^3^Department of Pathology, Rikshospitalet, Oslo University Hospital and University of Oslo, Oslo, Norway; ^4^Department of Oncology, Norwegian Radium Hospital, Oslo University Hospital, Oslo, Norway; ^5^Department of Genetics, Institute for Cancer Research, Norwegian Radium Hospital, Oslo University Hospital, Oslo, Norway; ^6^Institute of Clinical Medicine, University of Oslo, Oslo, Norway; ^7^Section of Oncology, Drammen Hospital, Vestre Viken Hospital Trust, Drammen, Norway

**Keywords:** tumor-infiltrating immune cells, human lung cancer, flow cytometry, immunomonitoring, NSCLC, immunoscore

## Abstract

Non-small cell lung cancer (NSCLC) is the leading cause of cancer-related death in the world. Immunological analysis of the tumor microenvironment (immunoscore) shows great promise for improved prognosis and prediction of response to immunotherapy. However, the exact immune cell composition in NSCLC remains unclear. Here, we used flow cytometry to characterize the immune infiltrate in NSCLC tumors, non-cancerous lung tissue, regional lymph node, and blood. The cellular identity of >95% of all CD45^+^ immune cells was determined. Thirteen distinct immune cell types were identified in NSCLC tumors. T cells dominated the lung cancer landscape (on average 47% of all CD45^+^ immune cells). CD4^+^ T cells were the most abundant T cell population (26%), closely followed by CD8^+^ T cells (22%). Double negative CD4^−^CD8^−^ T cells represented a small fraction (1.4%). CD19^+^ B cells were the second most common immune cell type in NSCLC tumors (16%), and four different B cell sub-populations were identified. Macrophages and natural killer (NK) cells composed 4.7 and 4.5% of the immune cell infiltrate, respectively. Three types of dendritic cells (DCs) were identified (plasmacytoid DCs, CD1c^+^ DCs, and CD141^+^ DCs) which together represented 2.1% of all immune cells. Among granulocytes, neutrophils were frequent (8.6%) with a high patient-to-patient variability, while mast cells (1.4%), basophils (0.4%), and eosinophils (0.3%) were less common. Across the cohort of patients, only B cells showed a significantly higher representation in NSCLC tumors compared to the distal lung. In contrast, the percentages of macrophages and NK cells were lower in tumors than in non-cancerous lung tissue. Furthermore, the fraction of macrophages with high HLA-DR expression levels was higher in NSCLC tumors relative to distal lung tissue. To make the method readily accessible, antibody panels and flow cytometry gating strategy used to identify the various immune cells are described in detail. This work should represent a useful resource for the immunomonitoring of patients with NSCLC.

## Introduction

Lung cancer is the leading cause of cancer-related death worldwide with a high annual incidence and a 5-year survival rate < 20% (1). Non-small cell lung cancer (NSCLC) is the most common type of lung cancers, representing ~90% of all cases (2). The two most prevalent NSCLC subtypes are adenocarcinoma and squamous cell carcinoma constituting 50 and 40% of all cases, respectively (3, 4). Currently, prognosis and selection of treatment are mainly based on the TNM staging system, which classifies the extent of cancer in four categories, stages I-IV, based on the size of the primary tumor (T), evidence of cancer cells in the regional lymph nodes (N), and presence of distal metastasis (M) (5). However, clinical outcome can vary greatly among patients within the same TNM stage (6).

Several reports indicated that the type, density, and location of immune cells within the tumor microenvironment play a central role in disease progression. In ovarian cancer (7), colorectal cancer (8, 9), breast cancer (10), and lung cancer (6, 11–13) immunological parameters were reported to better predict the clinical outcome than TNM staging. Therefore, it has been suggested that an immunoscore based on immunological analysis of the tumor microenvironment should be included as a separate component in the classification system (8). The benefits of including immune parameters in the TNM staging extend to individualized treatment selection (14). Recent development of immune checkpoint inhibitors, such as blocking antibodies against the CTLA-4 or PD-1/PD-L1 molecules, has greatly improved cancer treatment and prolonged patient survival. However, objective response rates to checkpoint blockade in NSCLC are currently only about 20% (15–18). Therefore, there is an urgent need to establish reliable methods to characterize the immune response in NSCLC tumors in order to be able to predict survival and response to immunotherapy for individual patients.

Early attempts to identify immune cells in NSCLC used immunohistochemistry (19–22). These studies revealed that NSCLC tumors contain numerous types of immune cells including T cells (23), B cells (24), macrophages (25), NK cells (26), and dendritic cells (DCs) (24, 26), and various associations between immune cell density and patient survival were reported (21, 27). However, these immunohistochemistry-based investigations typically used one single monoclonal antibody to identify a given immune cell type, which is generally not sufficient. To obtain a more reliable identification of immune cells in NSCLC, several recent reports used flow cytometry with multiple antibodies (28–30). The first flow cytometry study which investigated in detail a small group of patients (*n* = 6) with lung adenocarcinoma confirmed the presence of a large number of immune cell types in tumors (28). In contrast, a second study which focused on T cells only reported six different immune cell lineages in NSCLC tumors: CD4^+^ T cells, CD8^+^ T cells, granulocytes, monocytes, B cells, and NK cells (29). A surprising conclusion from a third study was that neutrophils were the most prevalent immune cell type in NSCLC tumors (30). Unfortunately, these studies included limited information about the flow cytometry gating strategy, making it challenging to compare the results (28–30). As a result of these conflicting data and unclear methodology, the exact immune cell content in NSCLC tumors remains undetermined.

In order to firmly establish the immune cell composition in NSCLC, we analyzed by 4-laser flow cytometry a large cohort of patients (*n* = 68), all operated at Oslo University Hospital. The exact cell type was determined for >95% of all CD45^+^ immune cells in NSCLC tumors. To make the method readily accessible to other laboratories, we present in detail the established antibody panels and the gating strategies used to identify the various immune cells. In total, thirteen different immune cell types were identified. In addition, four sub-populations of B cells and two subsets of NK cells were observed. This work should represent a useful resource for the establishment of an immunoscore for patient prognosis and treatment selection in NSCLC.

## Materials and Methods

### Ethics Statement

All samples were collected from patients diagnosed with NSCLC, operated at Oslo University Hospital between January 2013 and December 2016. All patients included in the study have signed a written informed consent. The study was approved by the Regional Committee for Medical and Health Research Ethics (Oslo, Norway, ref. S-05307).

### Patients and Clinical Materials

Tissue and blood samples were collected from patients undergoing lobectomy, bilobectomy or pneumonectomy. The patients were operated at the Department of Cardiothoracic Surgery at Rikshospitalet and Ullevål Hospitals, Oslo University Hospital, Oslo, Norway. Immunodeficient patients or patients who had received any previous cancer treatment were excluded from the study. Samples from 68 patients diagnosed with primary NSCLC stages IA to IIIB were examined (Table 1) (5). Of the 68 patients, 38 were diagnosed with adenocarcinoma, 26 with squamous cell carcinoma, and 4 patients were diagnosed with other, rare types of NSCLC (Table 1). Based on the smoking history, patients were separated into 3 groups: (i) active/present smokers (*n* = 32), (ii) former smokers (*n* = 28), and (iii) those who had never smoked (*n* = 8; denoted non-smokers, Table 1). Active or present smokers were patients who were actively smoking at the time of the operation and those who smoked at least up to 6 months prior to the operation. To be considered a former smoker, the patient had to have stopped smoking at the latest 6 months prior to the operation.

**Table 1:** Characterization of the patient population (*n* = 68).

Age–year	Mean	67.7
	Range	51–85
Gender (%)	Male	36 (53)
	Female	32 (47)
Smoking status^*^ (%)	Active/present	32 (47)
	Former	28 (41.1)
	Never (Non-smokers)	8 (11)
Histology (%)	Adenocarcinoma	38 (55.8)
	Squamous cell carcinoma	26 (38.2)
	Other^**^	4 (5.8)
pTNM stage and tumor diameter (%)	Ia 0–2 cm	16 (23.5)
	Ib 2–3 cm	17 (25)
	IIa 3–5 cm	3 (4.4)
	IIb 5–7 cm	18 (26.5)
	IIIa >7 cm	12 (17.6)
	IIIb >7 cm	2 (2.9)
Procedure (%)		
	Lobectomy	58 (85.3)
	Bilobectomy	3 (4.4)
	Pneumonectomy	7 (10.3)
Tumor location (%)	Right upper lobe	16 (23.5)
	Right middle lobe	6 (8.8)
	Right lower lobe	15 (22.1)
	Left upper lobe	17 (25)
	Left lower lobe	14 (20.6)
Concomitant disease (%)	COPD^***^	23 (33.8)
	Heart disease	19 (27.9)
	Diabetes	5 (7.3)

* *Active/present smokers were patients who were actively smoking at the time of the operation and those who smoked at least up to 6 months prior to the operation. To be considered a former smoker, the patient had to have stopped smoking at the latest 6 months prior to the operation*.

** *Other refers to: large cell carcinoma (n = 2), pleomorphic carcinoma (n = 2)*.

*** *COPD, chronic obstructive pulmonary disease*.

Four different samples were collected from the patients: a sample of the tumor, a sample of distal lung, half of a regional lymph node, and blood. The sample from non-cancerous lung tissue, termed distal lung, was sampled furthest away from the tumor in the resected lobe. Lymph nodes were sampled from station 10 (around the bronchus) of the resected lobe, after the lobe had been extracted from the patient. Lymph nodes were resected following the European guidelines for lung cancer surgery (31). Lymph node sampling did not influence the diagnostic process and only half of a lymph node per patient was used for research. The tissues were transported on ice in DMEM medium (Gibco, Thermo Fisher Scientific, Waltham, MA, USA, Cat-no: 10565-018) supplemented with 0.25 μg/mL amphotericin (Sigma-Aldrich, St. Louis, MO, USA, Cat-no: A2942). Tumor and distal lung samples were washed extensively (and squeezed) with DMEM/amphotericin in order to remove the blood as much as possible. To generate single cell suspensions, the tissues (tumor, distal lung and lymph node) were mechanically dissociated using scissors, and then incubated with DMEM supplemented with 2 mg/mL collagenase A (Roche, Basel, Switzerland, Cat-no: 10103586001) and 50 units/mL DNase (Roche, Cat-no: 11284932001) for 1 h, with stirring magnet, at 37°C. The digested tissues were filtered through a 100 μm filter (Falcon, Corning, NY, USA, Cat-no: 35236) to eliminate large cellular debris and aggregates. Single cell suspensions were centrifuged at 410 g for 6 min at 4°C, and the pellets were resuspended in DMEM with 10% fetal bovine serum (FBS) (Sigma-Aldrich, Cat-no: F7524). Blood was sampled from central venous catheter, immediately before the surgery, but after the patient had been anesthetized. Blood samples were kept at room temperature before isolation of peripheral blood mononuclear cells (PBMCs) using Lymphoprep density gradient according to the manufacturer's protocol (Axis-Shield, Dundee, UK, Cat-no: 07811).

### Flow Cytometry

Single cell suspensions of tumor, distal lung, lymph node and PBMCs were analyzed by flow cytometry. To block unspecific binding of antibodies, cells were incubated with 12.5 μg/mL mouse IgG (Sigma-Aldrich, Cat-no: I8765-10MG) diluted in phosphate buffered saline (PBS, Sigma, Ref-no: 14190-094) for 15 min on ice. Next, the cells were stained with fluorochrome-labeled monoclonal antibodies. We established 6 different antibody panels to identify the various immune cells (Supplementary Tables 1–6). To define positive and negative events, isotype-matched control antibodies were used. Antibodies were diluted in flow buffer consisting of PBS with 10% FBS (Sigma, Cat-no: F7524). Single cell suspensions from the tissues and the PBMCs were incubated with the antibody mix in 96 v-bottom well plates (Corning, Costar, Cat-no: 3894), on ice, in the dark, for 20 min. Following the incubation 100 μL of flow buffer was added to each well, and the plates were centrifuged at 410 g for 6 min at 4°C. Supernatants were discarded and cell pellets were re-suspended in 150 μL of flow buffer per well and centrifuged again (410 g for 6 min at 4°C). The cell pellets were re-suspended in 200 μL of flow buffer and filtered through a 100 μm mesh (Falcon, Cat-no: 352360). The cell suspensions were stained with 5 μg/mL propidium iodide (PI) immediately before analysis on BD LSRFortessa flow cytometer (BD Bioscience). Data were analyzed using FlowJo V10 software (FlowJo, LLC).

### Immunohistochemistry

Representative areas for the amount of inflammation in each tumor were chosen. The immunostaining was done on 2.5 μm thick FFPE tumor tissue using a Dako Autostainer instrument (Dako, Agilent Technologies, Santa Clara, California, USA, model Link 48). Epitope retrieval was performed with Dako Flex HpH according to the manufacturer's protocol (Dako EnVision FLEX, Cat-no: K8000). Tissue sections were incubated for 20 min with primary anti-CD45 monoclonal antibodies (clones 2B11 and PD7/26, diluted 1:300; Dako, Cat-no: M0701). The secondary detection was performed with Dako EnVision TM Flex (Dako, Cat-no: K8000) for 20 min, followed by diaminobenzidine (DAB) staining for 10 min (Dako EnVision FLEX, Cat-no. K800021-2). The slides were thereafter treated with 0.5% CuSO_4_ for 5 min before counterstaining with hematoxylin (Merck, Cat-no: 1.15938.0100) to visualize cell nuclei. Tissue sections were examined with a Nikon Eclipse model N *i*-U microscope (Nikon, Tokyo, Japan) equipped with Nikon Plan-Fluor objective lenses (2×, 10×, 20×, 40×, and 60×) and images were taken with an Infinity 2 digital camera (Lumenera Corporation, Nepean, Ontario, Canada).

### Statistics

Statistical calculations were performed using Graph Pad prism 6.0 (GraphPad). The percentages of the various immune cell populations were calculated from the total number of CD45^+^ live leukocytes. To determine whether the difference between three or more groups were statistically significant, we used non-parametric Kruskal-Wallis analysis of variance and *post-hoc* Dunn's multiple comparison test. The results were considered statistically significant when the *p*-value was < 0.05.

## Results

### Leukocytes Infiltrate Adenocarcinoma Lung Tumors in Higher Degree Than Distal Lung

**Figure 1:**
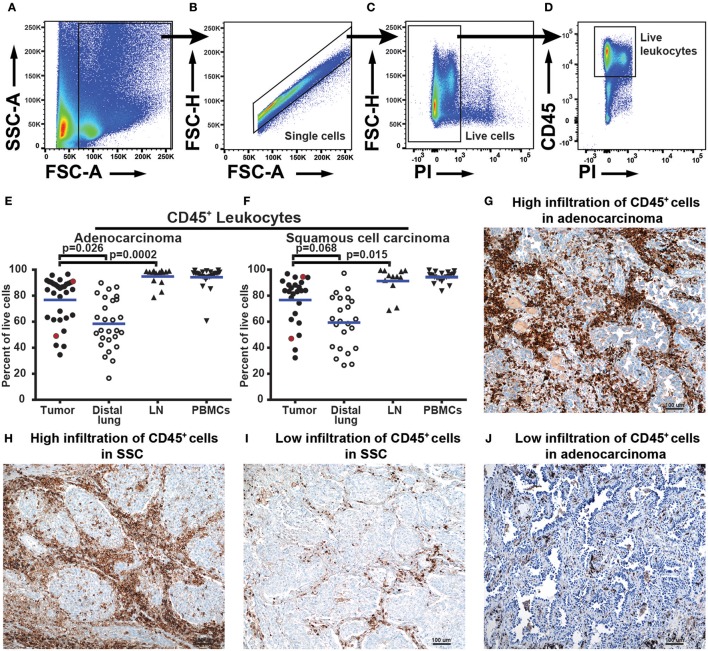
NSCLC tumors are infiltrated by various quantities of CD45^+^ leukocytes. **(A–F)** Data obtained by flow cytometry analysis of 57 patients, 32 with adenocarcinoma and 25 with squamous cell carcinoma. **(A)** The size of the events (FSC-A) was used to exclude cellular debris and define all cells. **(B)** Single cells were identified using FSC-A and FSC-H and doublets were gated out. **(C)** Live cells are negative for propidium iodide (PI) stain. **(D)** Live leukocytes were defined as CD45^+^PI^−^ cells. **(E)** and **(F)** show leukocytes (CD45^+^PI^−^) as percent of all live cells identified by flow cytometry of **(E)** adenocarcinoma and **(F)** squamous cell carcinoma. Each symbol in the graphs **(E,F)** represents data from one patient. Statistical calculations were performed with non-parametric Kruskal-Wallis analysis and Dunn's post-test, comparing tumor to distal lung and lymph node (LN). Red dots in graphs **(E)** and **(F)** show patient samples also analyzed by immunohistochemistry. **(G–J)** Immunohistochemistry analysis of tissue sections from tumors of four NSCLC patients. HE staining (blue) and anti-CD45 staining of immune cells (brown) at 100× magnifications. The area shown in the pictures is representative of the inflammation in the tumor as a whole. **(G)** Adenocarcinoma tumor showing high infiltration of CD45^+^ cells (91% of all cells in flow cytometry). **(H)** Squamous cell carcinoma tumor with high infiltration of CD45^+^ cells (96% of all cells in flow cytometry). **(I)** Low infiltration of CD45^**+**^ cells in squamous cell carcinoma (45% of all cells in flow cytometry). **(J)** Low infiltration of CD45^+^ cells in adenocarcinoma tumor (52% of all cells in flow cytometry).

To characterize the immune cell composition in NSCLC, we collected fresh samples from four different anatomical locations: the tumor, distal lung (non-cancerous lung tissue sampled furthest away from the tumor in the resected lobe), a regional lymph node, and blood. Single cells isolated from these samples were analyzed by multiparametric flow cytometry. In the gating strategy used to identify leukocytes, a nucleated cell gate based on FSC-A and SSC-A was set to remove debris from further analysis (Figure 1A). A single cell gate, based on FSC-A and FSC-H, was used to exclude doublets and cell aggregates (Figure 1B), and a gate with live cells was made using propidium iodide (PI) to exclude dead cells (Figure 1C). Next, the pan-leukocyte marker CD45 was used and live leukocytes were defined as CD45^+^PI^−^ cells (Figure 1D). The fraction of leukocytes among all live cells in adenocarcinoma and squamous cell carcinoma tumors varied from 34 to 96% across the patient cohort (Figures 1E,F). Leukocyte infiltration in NSCLC tumors was apparently not influenced by cortical steroid treatment of some of the patients suffering from chronic obstructive pulmonary disease (COPD) (Supplementary Figure 1), as suggested by a recent report (32). The percentage of leukocytes was significantly higher in adenocarcinoma tumor tissue compared to the distal lung (Figure 1E *p* = 0.026). A similar trend was observed for squamous cell carcinoma but the difference did not reach statistical significance (*p* = 0.068, Figure 1F). Immunohistological staining of CD45 on NSCLC tumor tissue sections was performed on a few, randomly selected samples and the results supported the findings obtained by flow cytometry. Patient samples with a high number of CD45^+^ leukocytes in flow cytometry also showed an abundant number of CD45^+^ cells in immunostained tissue sections (Figures 1G–H). Conversely, a lower frequency of CD45^+^ cells measured by flow cytometry corresponded with observation of fewer infiltrating CD45^+^ cells in immunostained tissue sections (Figures 1I,J).

### T Cells Dominate the Immune Cell Composition in NSCLC

**Figure 2:**
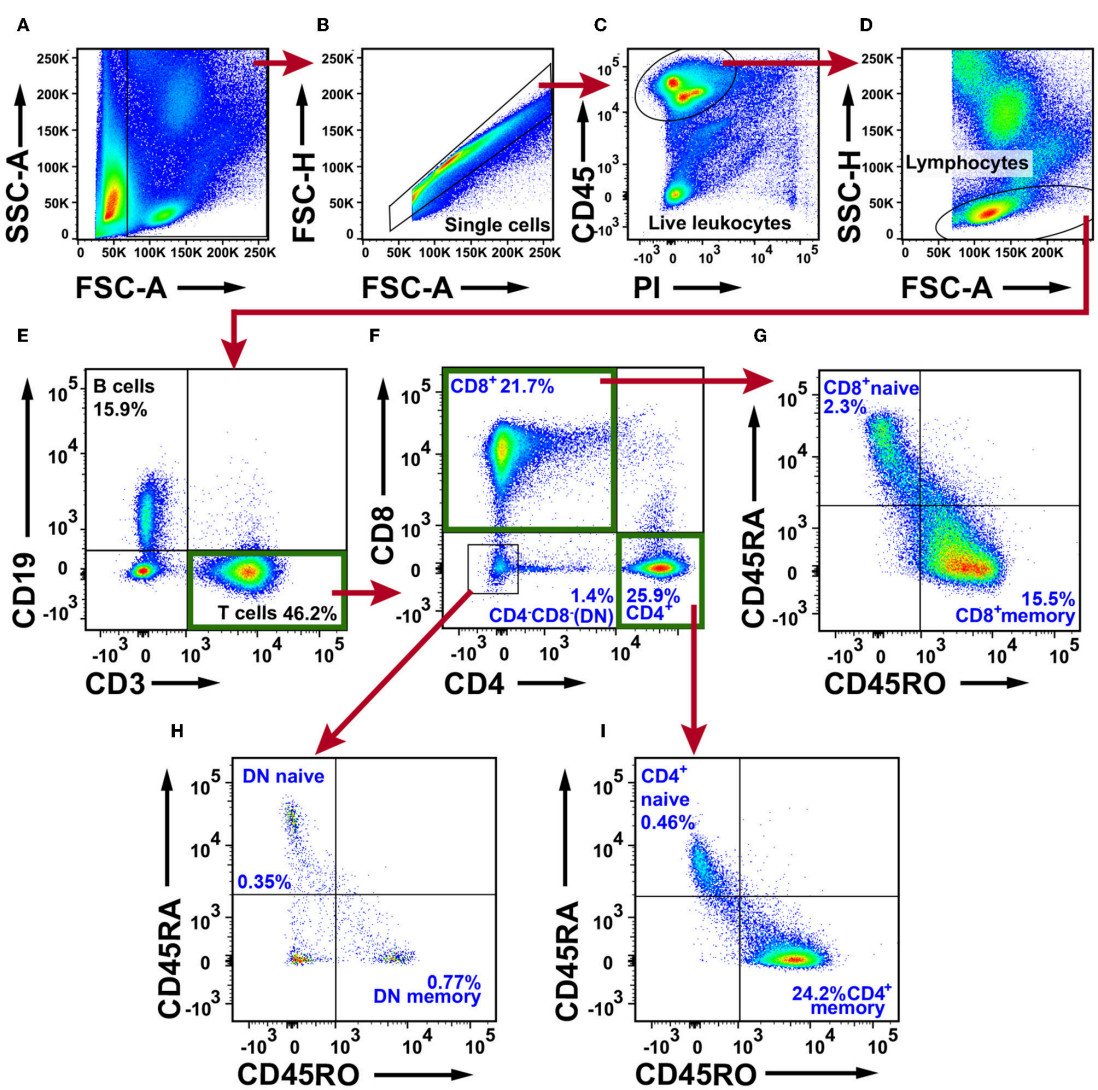
Flow cytometry analysis of T cells in NSCLC tumor tissue. **(A)** Gate for nucleated cells based on the size and complexity of the event (FCS-A and SSC-A, respectively). **(B)** Nucleated cells were further plotted in FSC-A and FSC-H to gate single cells and exclude doublets. **(C)** From the single cell gate, live leukocytes were defined as CD45^+^PI^−^. **(D)** A lymphocyte gate was made based on FSC-A and SSC-H. **(E)** CD19^+^ B cells were excluded from the lymphocyte population, and the CD19^−^CD3^+^ population was defined as T cells. **(F)** T cells were further divided in CD4^+^, CD8^+^, and CD4^−^CD8^−^ populations. Each subset was examined for the naive phenotype CD45RA^+^CD45RO^−^ and the effector/memory phenotype CD45RA^−^CD45RO^+^. **(G)** Naive/memory phenotyping of CD8^+^ T cells. **(H)** Naive/memory phenotyping of CD4^−^CD8^−^ T cells. **(I)** Naive/memory phenotyping of CD4^+^ T cells. The percentages presented in the figure are average values of all NSCLC patients analyzed for T cells (*n* = 30; 15 adenocarcinoma, 14 squamous cell carcinoma, one large cell carcinoma). Percentages were calculated from the total number of live leukocytes (CD45^+^PI^−^ population). DN, double negative T cells.

To investigate the T cell content in NSCLC tumors we first defined single, live, CD45^+^ cells, denoted live leukocyte population (Figures 2A–C). A lymphocyte gate was set based on the FSC and SSC parameters (Figure 2D). The lymphocytes were identified based on CD3 and CD19 expression and three populations were observed: CD19^−^CD3^+^ T cells, CD19^+^CD3^−^ B cells, and CD19^−^CD3^−^ double negative cells (Figure 2E). T cells were further examined for the expression of CD4 and CD8, which resulted in three populations: CD4^+^ T cells, CD8^+^ T cells, and CD4^−^CD8^−^ double negative (DN) T cells (Figure 2F). Each of the three T cell populations exhibited a memory/effector or a naive phenotype defined by expression of the surface markers CD45RO and CD45RA, respectively (Figures 2G–I). The majority of T cells were CD45RA^−^CD45RO^+^ memory/effector cells. The fact that the ratio of naïve/memory T cells was much lower in tumor tissue (Figure 2) as compared with PBMCs (Supplementary Figure 2) shows that the blood contamination was very low in tumor samples. Furthermore, although naïve T cells are classically being defined as CD45RA^+^CD45RO^−^, it should be noted that CD45RA^+^ effector/memory CD8^+^ T cells (the so called T_EMRA_ cells) have been reported (33, 34). Immunofluorescence staining of NSCLC tumor tissue sections revealed the presence of CD45RA^+^CD3^+^ T cells in both the tumor stroma (presumably T_EMRA_ cells) and in the tertiary lymphoid structures (TLS) that are formed at the tumor periphery (24) (Supplementary Figure 3). CD45RA^+^CD3^+^ T cells in TLS are likely to be naïve T cells (at least for some of those) because CD45RA^+^CD3^+^ naïve T cells have been reported to be enriched in TLS in NSCLC (35).

**Figure 3:**
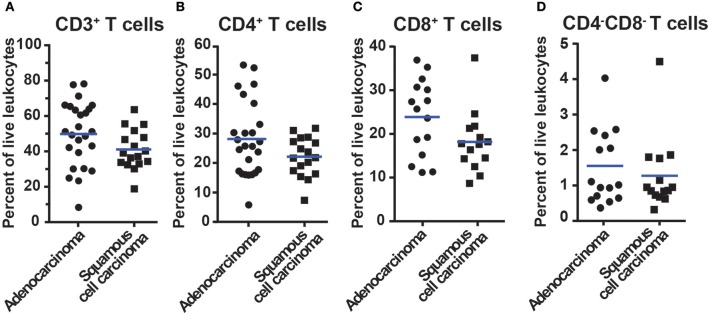
Percentage of T cells in NSCLC tumors of different histological types. Data for adenocarcinoma and squamous cell carcinoma are presented. The gating strategy is described in Figure 2. Percentages of **(A)** All CD3^+^ T cells, **(B)** CD3^+^CD4^+^ T cells, **(C)** CD3^+^CD8^+^ T cells, and **(D)** CD3^+^CD4^−^CD8^−^ T cells, i.e., double negative T cells. The percentages of T cells were calculated from the total number of live leukocytes (CD45^+^PI^−^) in the sample. Each symbol represents data for one patient (*n* = 42; 25 adenocarcinoma, 17 squamous cell carcinoma), and mean values are indicated with blue lines. Statistical calculations were performed with non-parametric Kruskal-Wallis analysis and Dunn's post-test, and no significant differences were observed between the groups.

CD3^+^ T cells constituted on average 49.5% in adenocarcinoma and 41.1% in squamous cell carcinoma of all tumor-infiltrating CD45^+^ leukocytes (Figure 3A). Among CD3^+^ T cells, CD4^+^ T cells were the most frequent (28.6% in adenocarcinoma and 22.1% in squamous cell carcinoma, Figure 3B) followed by CD8^+^ T cells (23.9% in adenocarcinoma and 18.2% in squamous cell carcinoma, Figure 3C). The CD4^−^CD8^−^, DN T cells amounted to a small portion of the T cell population (1.5% in adenocarcinoma and 1.3% in squamous cell carcinoma, Figure 3D). In subsequent analyses, we compared the CD3^+^ T cell representation between different anatomical sites (tumor, distal lung, blood, lymph node), histological tumor subtypes, tumor stages, and smoking histories. No statistically significant differences were observed between tumor and distal lung samples (Supplementary Figures 4, 5). Taken together these data show that CD4^+^ T cells represent the most abundant population of T cells in NSCLC (25.9%) followed by CD8^+^ T cells (21.7%). The CD4^−^CD8^−^ T cells only made a small fraction of tumor-infiltrating immune cells (1.4%). The majority of T cells in tumor are memory/effector T cells.

### B Cells Are Abundant and Diverse in NSCLC Tumors

**Figure 4:**
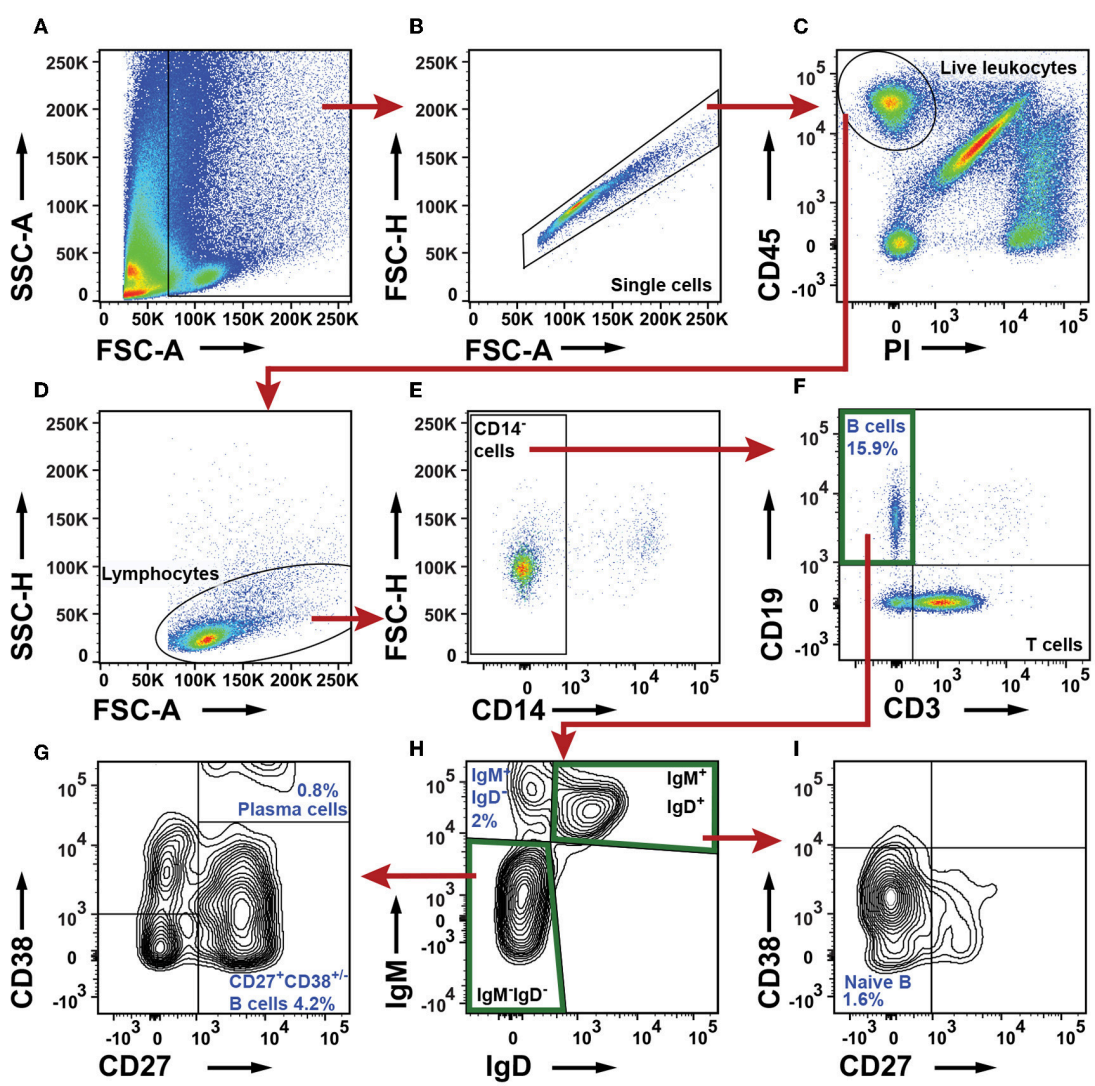
Flow cytometry analysis of B cell sub-populations in NSCLC tumors. **(A)** The FSC-A and SSC-A plot was used to identify nucleated cells. **(B)** In the FSC-A and FSC-H plot single cells were gated and doublets were excluded. **(C)** Live leukocytes were defined as CD45^+^PI^−^. **(D)** A lymphocyte gate was set in a FSC-A and SSC-H plot. **(E)** CD14^+^ macrophages were excluded. **(F)** B cell gate defining all B cells as CD19^+^ and CD3^−^. **(G–I)** Determination of B cell sub-populations. **(G)** Characterization of IgM^−^IgD^−^ B cells. Two populations were identified: CD27^+^CD38^++^ plasma cells and CD27^+^CD38^+/−^ cells **(H)** A IgD/IgM plot was used to identify an IgM^+^IgD^−^ B cell sub-population and to define IgM^−^IgD^−^ and IgM^+^IgD^+^ gates. **(I)** Further characterization of IgM^+^IgD^+^ B cells: naive B cells are defined as CD27^−^CD38^+/−^. The percentage numbers presented in the figure are average values of all NSCLC patients analyzed for the indicated B cell sub-populations (*n* = 23; 12 adenocarcinoma, 11 squamous cell carcinoma). For each patient, the percentages of B cells and their sub-populations were calculated from the total number of live leukocytes.

To characterize the B cells in NSCLC tumors, we first excluded debris, cell clumps, dead cells, and CD45^−^ cells, and defined a lymphocyte gate (Figures 4A–D). From this lymphocyte population, CD14^+^ macrophages were excluded (Figure 4E). The remaining cells were separated based on expression of CD3 and CD19 into three populations: CD19^−^CD3^+^ T cells, CD19^+^CD3^−^ B cells, and a CD19^−^CD3^−^ double negative population (Figure 4F). For further analysis of B-cell sub-populations, we used the nomenclature established by Jackson et al. and Germain et al. (36, 37). IgM and IgD expression patterns were used to identify three populations of cells: IgM^+^IgD^+^, IgM^−^IgD^−^, and IgM^+^IgD^−^ B cells (Figure 4H). From the IgM^−^IgD^−^ DN population, CD27 and CD38 were used to identify a CD27^+^CD38^+/−^ B-cell sub-population as well as CD27^+^CD38^++^ plasma cells (Figure 4G). The CD27^+^CD38^+/−^ B-cell sub-population may consist of either memory B cells or germinal center B cells, or both (38, 39). From the IgM^+^IgD^+^ population, CD27 and CD38 were used to identify naïve B cells being CD38^+/−^CD27^−^ (Figure 4I). For comparison, the gating strategy for B cell sub-populations in blood (PBMCs) is shown in Supplementary Figure 6.

**Figure 5:**
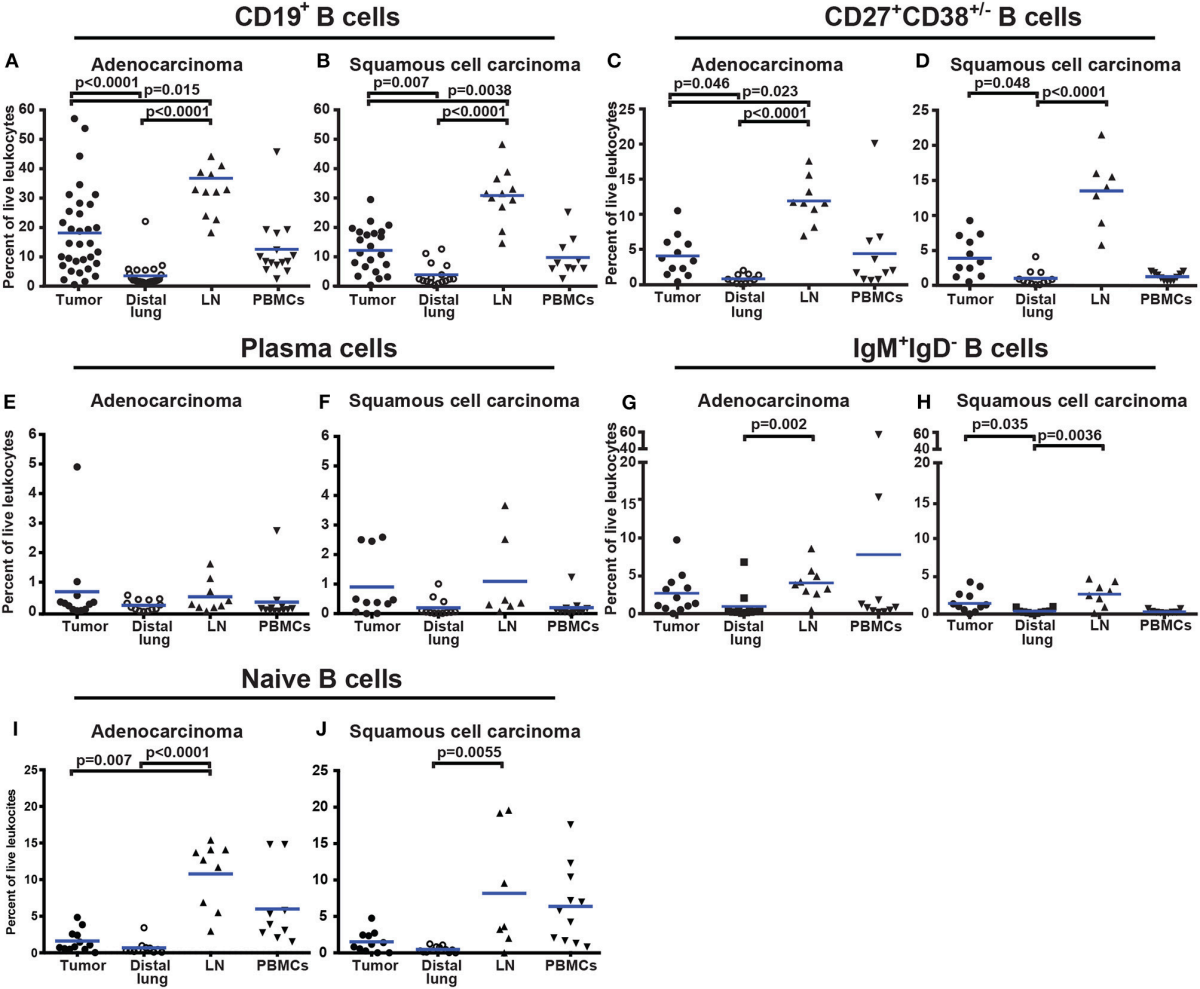
Percentage of all CD19^+^ B cells and B-cell sub-populations in different tissues from NSCLC patients. **(A,B)** Comparison of percentages of CD19^+^ B cells in patients diagnosed with **(A)** adenocarcinoma (*n* = 33) and **(B)** squamous cell carcinoma (*n* = 23). **(C,D)** Percentage of CD27^+^CD38^+/−^ B cells in tissues of patients diagnosed with **(C)** adenocarcinoma (*n* = 12) and **(D)** squamous cell carcinoma (*n* = 11). **(E)** Presence of plasma cells in adenocarcinoma (*n* = 12) and **(F)** in squamous cell carcinoma (*n* = 11). **(G)** Percentage of IgM^+^IgD^−^ B cells in adenocarcinoma (*n* = 12) and **(H)** squamous cell carcinoma (*n* = 11). **(I)** Percentage of naïve B cells in adenocarcinoma (*n* = 12) and **(J)** squamous cell carcinoma (*n* = 11). The cells were gated as indicated in Figure 4. Each symbol represents data from one patient as percentage of the total number of live leukocytes (CD45^+^PI^−^) and blue lines indicate mean values. Statistical analysis was performed using non-parametric Kruskal-Wallis analysis and Dunn's post-test comparing tumor, distal lung, and lymph node (LN).

CD19^+^ B cells constituted on average 18% of all CD45^+^ immune cells in adenocarcinoma and 12.7% in squamous cell carcinoma (Figures 5A,B). Comparison of percentage of B cells in different tissues revealed an increased infiltration of CD19^+^ B cells in tumor compared to distal lung (Figures 5A,B). This was evident in both adenocarcinoma (*p* < 0.0001) and squamous cell carcinoma (*p* = 0.007). The increased percentage of B cells in tumor may be a consequence of an increased number of CD27^+^CD38^+/−^ B cells (Figures 5C,D). The percentage of plasma cells was similar between tumor and distal lung (Figures 5E,F). In squamous cell carcinoma, the percentage of IgM^+^IgD^−^ B cells was higher in tumor compared to distal lung (Figures 5G,H). The percentage of naïve B cells was similar between tumor and distal lung (Figures 5I,J). There was a high variation in the percentage of tumor infiltrating CD19^+^ B cells between the individual patients (Figures 5A,B). The percentages of intratumoral B cells were not related to the histological type of tumor, disease stage, or patients smoking history (Supplementary Figures 7–9).

### NSCLC Tumors Contain Macrophages With High HLA-DR Expression and Three Subsets of Dendritic Cells (DCs)

**Figure 6:**
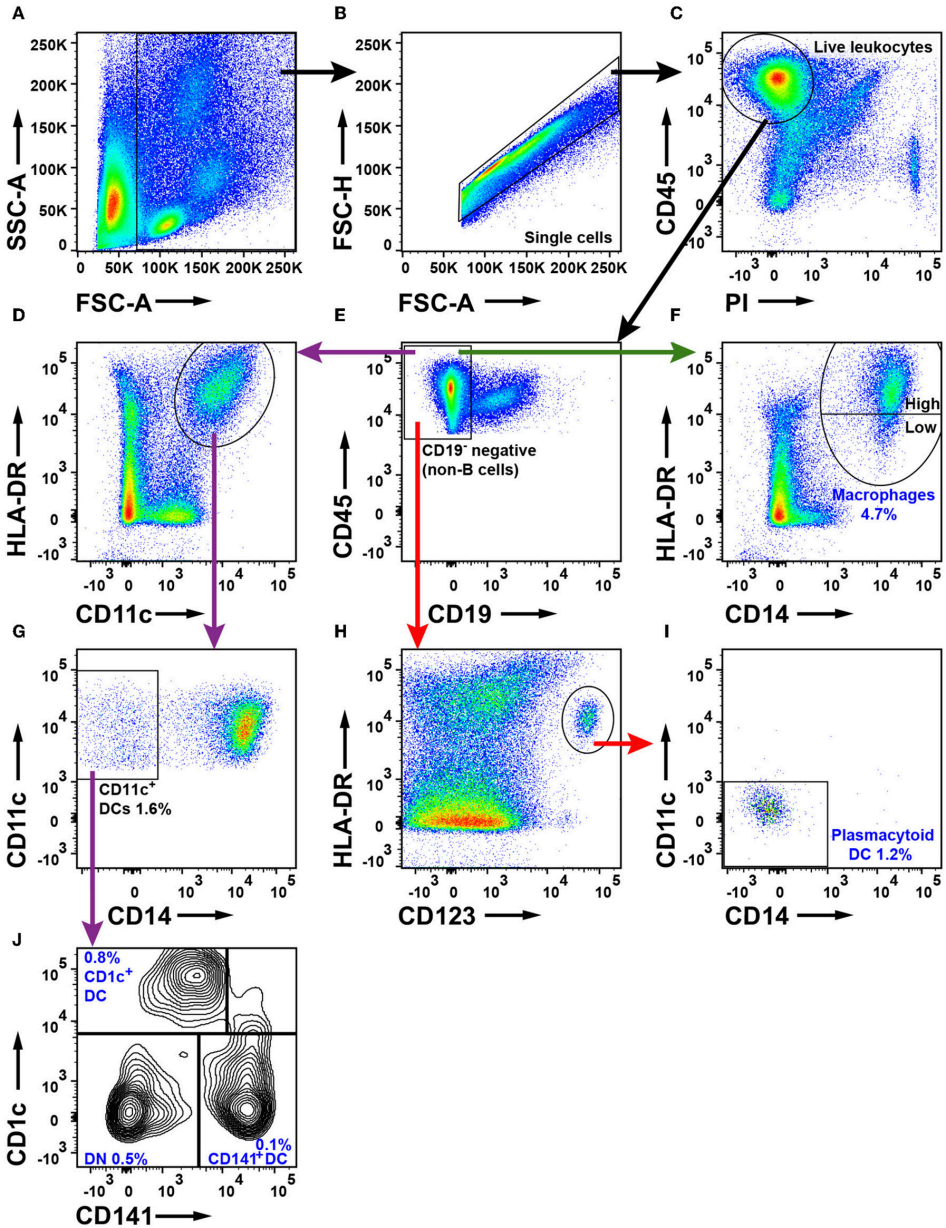
Flow cytometry analysis of macrophages and DCs in NSCLC tumors. **(A)** FSC-A and SSC-A were used to gate nucleated cells. **(B)** FSC-A and FSC-H were used to gate single cells and exclude doublets. **(C)** Live leukocytes were defined as CD45^+^PI^−^. **(E)** Exclusion of CD19^+^ B cells. **(D)** Gate for HLA-DR^+^ and CD11c^+^ cells. **(F)** Macrophages were defined as CD14^+^HLA-DR^+^ cells. HLA-DR expression on macrophages was considered either high or low as shown in the plot. **(G)** Myeloid DCs were defined as CD11c^+^CD14^−^. **(H)** Plasmacytoid DCs were defined as HLA-DR^+^CD123^+^ and also **(I)** CD11c^−^ and CD14^−^. **(J)** Two subsets of myeloid DCs were identified: CD141^+^ DCs and CD1c^+^ DCs. A double negative (DN) population was also observed. The percentages of the cell populations shown in the figure were calculated from the total number of live leukocytes and represent average values from 30 patients (16 adenocarcinoma, 13 squamous cell carcinoma and one large cell carcinoma).

To characterize mononuclear phagocytes in NSCLC, we first identified the live leukocytes (Figures 6A–C) and excluded CD19^+^ B cells (Figure 6E). Three separate strategies were used to identify macrophages and DCs as shown by differently colored arrows in Figure 6. Macrophages were defined as HLA-DR^+^CD14^+^ cells (green arrow, Figure 6F). HLA-DR and CD123 were used to identify HLA-DR^+^CD123^+^ plasmacytoid dendritic cells (pDCs, red arrow, Figure 6H). This population was examined for CD14^+^ and CD11c^+^ expression (red arrow, Figure 6I) to ensure that macrophages and other DCs had been excluded from the gate defining pDCs. To identify myeloid dendritic cells (mDCs), we used HLA-DR and CD11c and observed a distinct population of HLA-DR^+^CD11c^+^ cells (purple arrow, Figure 6D). From the HLA-DR^+^CD11c^+^ population, CD14^+^ macrophages were excluded (Figure 6G). To investigate the mDCs more closely, we used the markers CD1c and CD141 (purple arrow, Figure 6J). We observed CD141^−^CD1c^+^ DCs, CD141^+^CD1c^−^ DCs, and a third population of CD141^−^CD1c^−^ DN cells. CD14^+^HLA-DR^+^ macrophages constituted 4.7% of all tumor-infiltrating leukocytes whereas the three DC populations together represented 2.1% of all leukocytes in tumor. Plasmacytoid DCs were the main DC subset in NSCLC tumors (1.2%), followed by CD1c^+^ mDCs (0.8%) and CD141^+^ DCs (0.1%). It should be noted that CD11c is not a specific marker for DCs in humans, because all human monocytes in blood express CD11c (40). Furthermore, *bona fide* CD14^+^HLA-DR^+^ macrophages in NSCLC tumors also express CD11c (as well as CD11b) (Supplementary Figure 10). Therefore, the CD11c^+^HLA-DR^+^CD14^−^CD1c^−^CD141^−^ “DN” cell population which constituted 0.5% of the leukocytes in tumor (Figure 6J) may contain either CD1c^−^CD141^−^ mDCs or CD14^−^ macrophages (or both). The gating strategy for monocytes and DCs in PBMCs is shown in Supplementary Figure 11. The percentages of DCs in tumor did not seem to vary in NSCLC patients with different disease stages, except possibly for CD141^+^ DCs which were more frequent in stage I compared to stage II NSCLC, although the *p*-value was borderline significant (*p* = 0.04) and will need to be confirmed with a larger patient cohort (Supplementary Figure 12).

**Figure 7:**
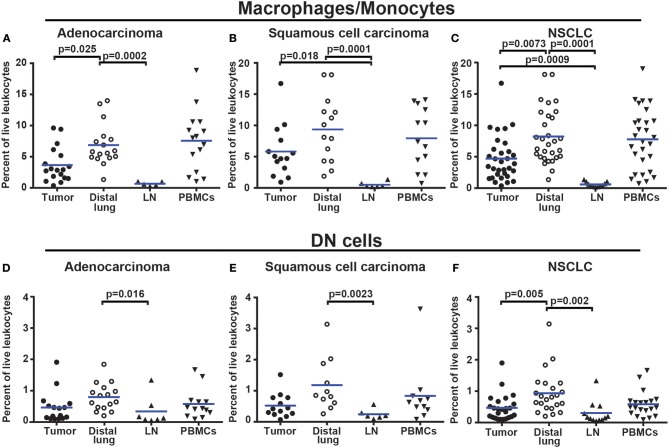
Macrophages and DN myeloid cells are less abundant in NSCLC tumors than in distal lung. **(A)** Percentages of macrophages/monocytes in adenocarcinoma patients (*n* = 18), **(B)** squamous cell carcinoma patients (*n* = 14), and **(C)** NSCLC (*n* = 33). **(D)** Percentages of DN myeloid cells (presumably DCs and/or macrophages) in adenocarcinoma (*n* = 16), **(E)** squamous cell carcinoma (*n* = 13), and **(F)** NSCLC (*n* = 29). Cells were gated as shown in Figure 6. The percentages were calculated from the total number of live leukocytes (CD45^+^PI^−^). Each symbol represents data from one patient. Mean values are indicated by blue lines. Statistical calculations were performed with non-parametric Kruskal-Wallis analysis and Dunn's post-test comparing tumor, distal lung, and lymph node (LN).

**Figure 8:**
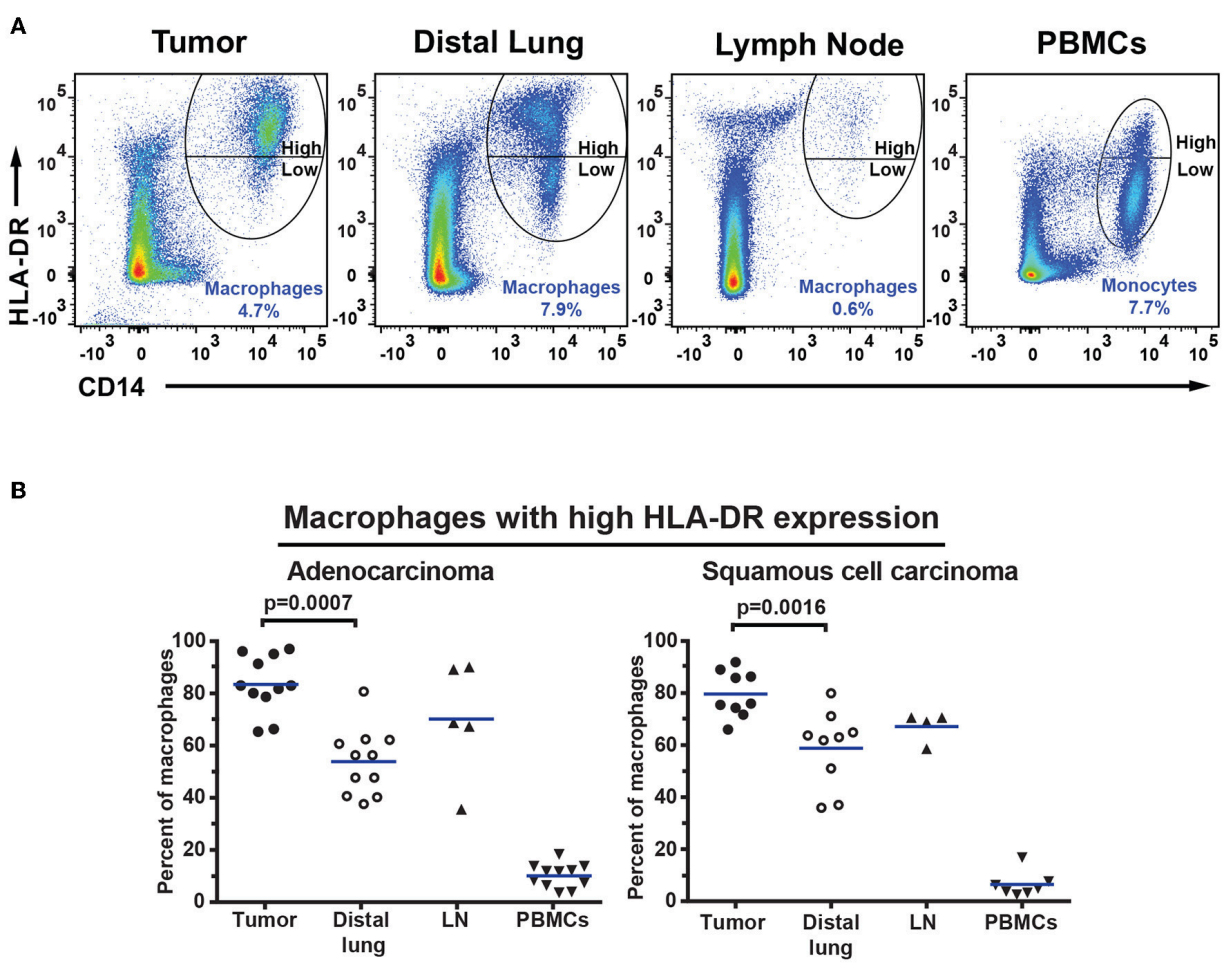
Macrophages in NSCLC tumors express high levels of HLA-DR. **(A)** Macrophages and monocytes were divided into two populations based on the expression level of HLA-DR on the surface: those with high and those with low HLA-DR expression as indicated. **(B)** Percentages of macrophages expressing high levels of HLA-DR in patients with adenocarcinoma (*n* = 11) and squamous cell carcinoma (*n* = 9). Cells were gated as shown in Figure 6. The percentages were calculated from the total number of macrophages/monocytes defined as CD14^+^HLA-DR^+^ cells. Each symbol represents data from one patient. Mean values are indicated by blue lines. Statistical calculations were performed with non-parametric Kruskal-Wallis analysis and Dunn's post-test comparing tumor, distal lung, and lymph node (LN).

The percentage of CD14^+^HLA-DR^+^ macrophages was lower in tumor (for adenocarcinoma and all NSCLC patients) compared to distal lung (Figures 7A–C). No significant difference was observed in the percentage of DC subsets (of all CD45^+^ leukocytes) between tumor and distal lung (Supplementary Figure 13). However, further data analysis suggested that the relative frequency (as percentage of all DCs) may be different between tissues, with pDCs being more frequent and CD1c^+^ mDCs being less frequent in tumor compared to distal lung tissue (Supplementary Figures 14, 15). We also observed a decreased percentage of infiltrating CD11c^+^HLA-DR^+^CD14^−^CD141^−^CD1c^−^ “DN” cells in tumor compared to distal lung when considering all NSCLC patients investigated (Figures 7D–F). Furthermore, a fraction of the macrophages was found to express high levels of HLA-DR (Figure 6F), and the HLA-DR expression levels appeared to be higher on macrophages in tumor, distal lung, and lymph node compared to monocytes in PBMCs (Figure 8A). In fact, some monocytes in the blood of NSCLC patients had very low or absent HLA-DR expression (Figure 8A) and may correspond to the previously reported CD14^+^HLA-DR^−/low^ myeloid-derived suppressor cell population (41). We further analyzed the macrophage population by separating the cells into those with high and low HLA-DR expression (Figure 8A). Using this criterion, we found that the fraction of HLA-DR^high^ macrophages was larger in tumor relative to the distal lung both in adenocarcinoma and squamous cell carcinoma (Figure 8B). These intratumoral HLA-DR^high^ macrophages may potentially represent interferon-γ (IFN-γ) activated macrophages with antitumor activity (42–46), since IFN-γ has been shown to specifically induce HLA-DR expression on macrophages (47).

### Adenocarcinoma Tumors Have a Reduced Percentage of NK Cells

**Figure 9:**
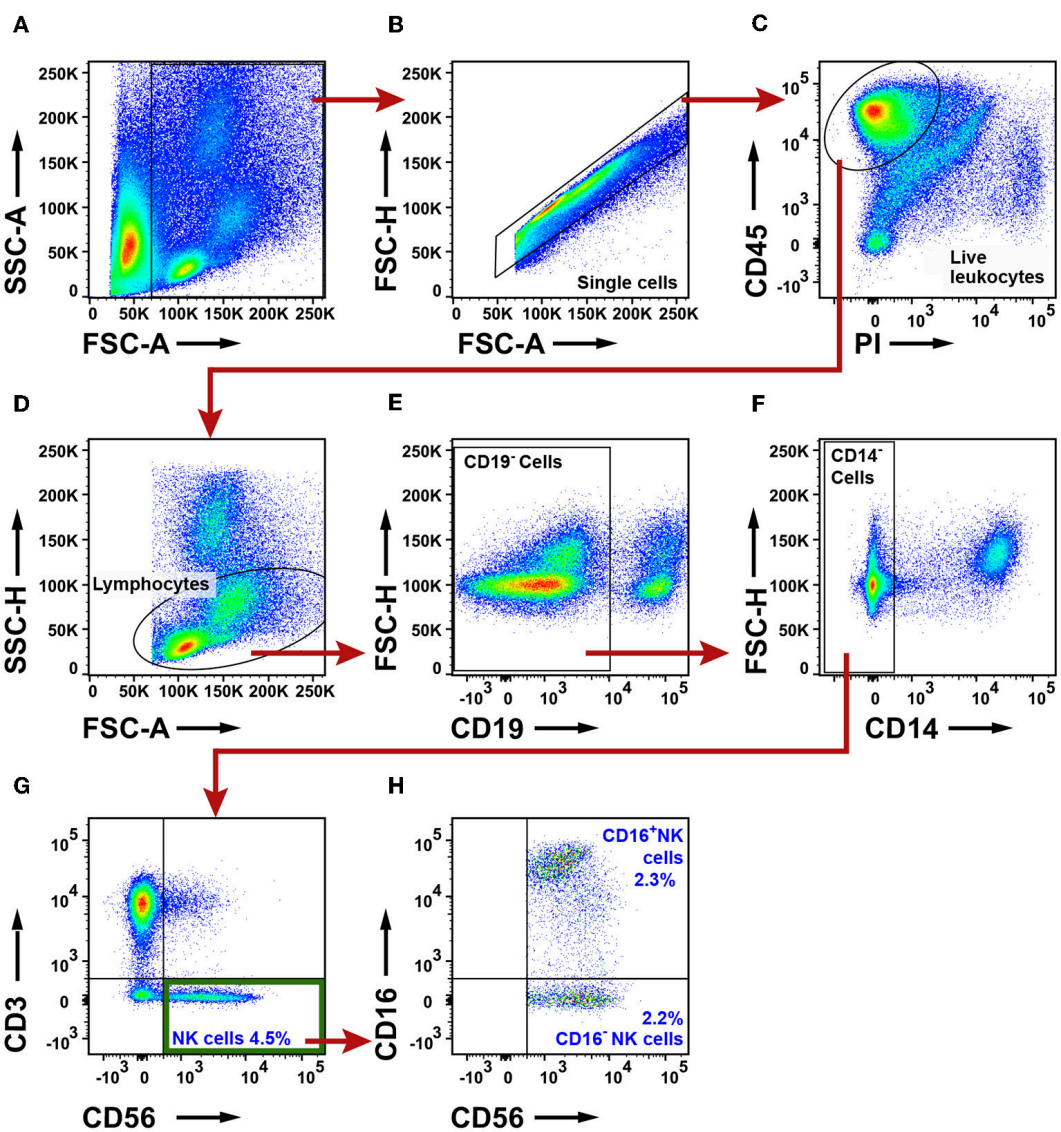
Flow cytometry analysis of NK cells in NSCLC tumors. **(A)** A FSC-A and SSC-A plot was used to identify nucleated cells. **(B)** Single cells were gated and doublets were excluded. **(C)** Live leukocytes were defined as CD45^+^PI^−^ cells. **(D)** A lymphocyte gate was set based on the size and granularity of the cells. **(E)** CD19^+^ B cells were excluded. **(F)** CD14^+^ macrophages were excluded. **(G)** NK cells were defined as CD3^−^CD56^+^. **(H)** Two NK cell subsets were identified: CD16^+^ and CD16^−^ NK cells. The percentages of all the populations were calculated from the total number of live leukocytes, and average values from 19 patients are presented (11 adenocarcinoma, eight squamous cell carcinoma).

**Figure 10:**
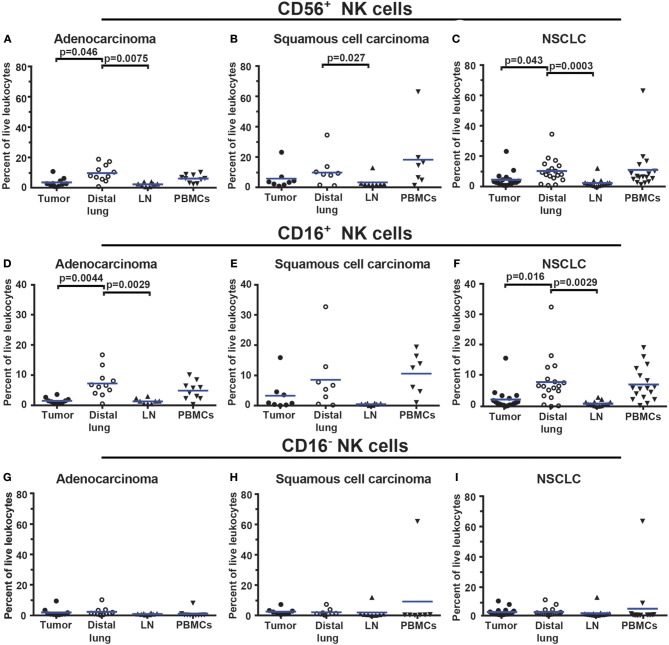
The percentage of NK cells is lower in NSCLC tumors compared to distal lung. **(A–C)** Percentages of all CD56^+^ NK cells in different tissues of patients diagnosed with **(A)** adenocarcinoma (*n* = 11), **(B)** squamous cell carcinoma (*n* = 8), and **(C)** NSCLC (*n* = 19). **(D–F)** Percentages of CD56^+^CD16^+^ NK cells in **(D)** adenocarcinoma, **(E)** squamous cell carcinoma and **(F)** NSCLC. **(G–I)** Percentages of CD56^+^CD16^−^ NK cells in **(G)** adenocarcinoma, **(H)** squamous cell carcinoma, and **(I)** NSCLC. The gating strategy is presented in Figure 9. Each symbol represents data from one patient, as percentage of all live leukocytes (CD45^+^PI^−^). Mean values are indicated with blue lines. Statistical calculations were performed with non-parametric Kruskal-Wallis analysis and Dunn's post-test comparing tumor, distal lung and lymph node (LN).

To identify NK cells, a lymphocyte gate was used (Figures 9A–D). CD19^+^ B cells and CD14^+^ macrophages were excluded (Figures 9E,F). The remaining cells were separated based on the expression of CD3 and CD56 into CD3^+^ T cells and CD3^−^CD56^+^ NK cells (Figure 9G). The CD3^+^CD56^+^ double-positive cell population observed in Figure 9G may consist of either NK T cells or conventional T cells expressing the NK/NKT marker CD56 (48). Additional markers would be required to distinguish NK T cells from conventional T cells expressing CD56. CD3^−^CD56^+^ NK cells were further investigated for expression of CD16, defining two subsets, CD16^+^ and CD16^−^ NK cells, respectively (Figure 9H). The gating strategy for NK cells in PBMCs is shown in Supplementary Figure 16. The total NK cell population made 4.5% of the live leukocytes in tumor. The percentages of CD16^−^ NK cells (2.2%) and CD16^+^ NK cells (2.3%) were similar. The percentage of all CD56^+^ NK cells was found to be lower in tumor (for adenocarcinoma and for all NSCLC patients) compared to distal lung (Figures 10A–C). This difference seemed to be essentially due to a reduced percentage of the CD16^+^ subset of NK cells in the tumor tissue (Figures 10D–F). In contrast, the percentage of CD16^−^ NK cells showed little variation between tissues (Figures 10G–I). Thus, NSCLC tumors contain two populations of NK cells (CD16^+^ and CD16^−^), and the percentage of NK cells is reduced in tumor compared to non-cancerous lung tissue.

### NSCLC Tumors Contain Numerous Neutrophils

**Figure 11:**
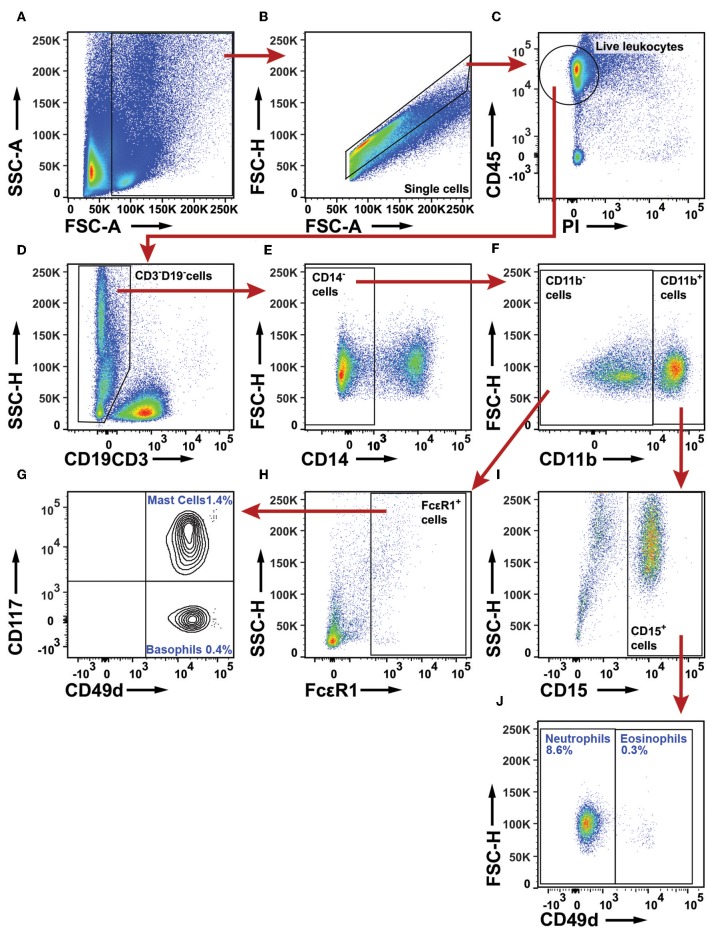
Flow cytometry analysis of granulocytes in NSCLC tumors. **(A)** A FSC-A and SSC-A plot was used to identify nucleated cells. **(B)** Single cells were gated and doublets were excluded. **(C)** Live leukocytes were defined as CD45^+^PI^−^ cells. **(D)** CD3^+^ T cells and CD19^+^ B cells were excluded. **(E)** CD14^+^ macrophages were excluded. **(F)** CD11b was used to separate granulocytes: CD11b^−^ cells include mast cells and basophils, whereas CD11b^+^ cells include neutrophils and eosinophils. **(G)** In the population of CD49d^+^ cells, basophils were distinguished from mast cells by use of CD117. **(H)** Mast cells and basophils are both defined as FcεR1^+^ cells. **(I)** From the CD11b^+^ leukocyte population, neutrophils and eosinophils were defined as CD15^+^ cells. **(J)** In the CD15^+^ population, neutrophils are CD49d^−^ and eosinophils CD49d^+^. The percentages indicated for each granulocyte population were calculated from the total number of live leukocytes for 18 NSCLC patients (11 adenocarcinoma and seven squamous cell carcinoma).

**Figure 12:**
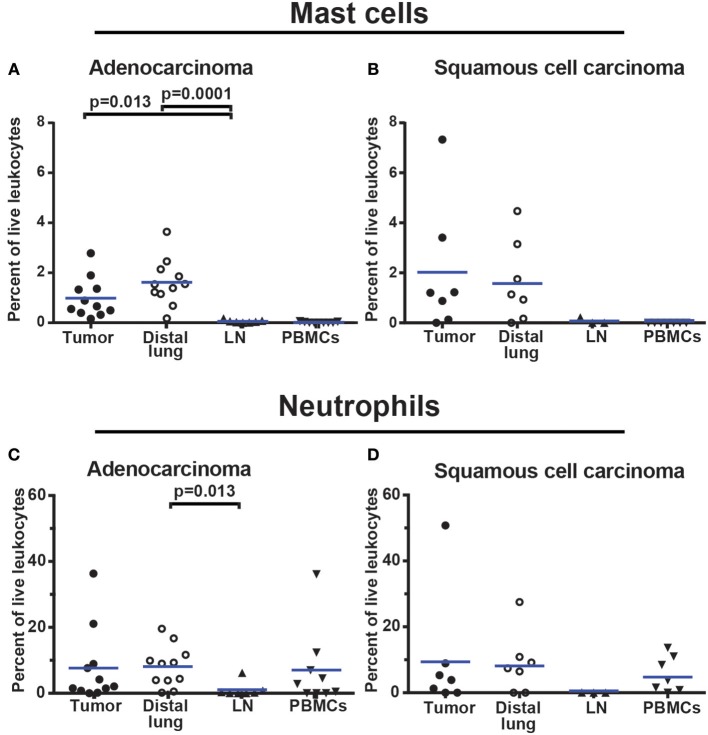
Granulocyte infiltration in NSCLC tumors. **(A,B)** Mast cell infiltration in **(A)** adenocarcinoma (*n* = 11) and **(B)** squamous cell carcinoma (*n* = 7). **(C,D)** Neutrophil infiltration in **(C)** adenocarcinoma (*n* = 11) and **(D)** squamous cell carcinoma (*n* = 7). Each symbol represents data from one patient as percentage of all live leukocytes (CD45^+^PI^−^). The gating strategy is described in Figure 11. Mean values are indicated with blue lines. Statistical calculations were performed with non-parametric Kruskal-Wallis analysis and Dunn's post-test comparing tumor, distal lung, and lymph node (LN).

To examine the granulocyte infiltrate, a live leukocyte gate was used (Figures 11A–C). CD19^+^ B cells, CD3^+^ T cells, and CD14^+^ macrophages were excluded (Figures 11D,E). The remaining leukocytes were separated based on CD11b expression (Figure 11F). The CD11b^−^ population was examined for FcεR1α (Figure 11H). FcεR1α^+^ cells were separated into CD117^+^CD49d^+^ mast cells, and CD117^−^CD49d^+^ basophils (Figure 11G). From the CD11b^+^ population (Figure 11F), cells expressing CD15 were selected (Figure 11I) and further separated into CD49d^−^ neutrophils and CD49d^+^ eosinophils (Figure 11J). The gating strategy for granulocytes in PBMCs is shown in Supplementary Figure 17 (note that the Lymphoprep density gradient used to prepare PBMCs removed most neutrophils which typically represent 50% of all leukocytes in blood). On average, granulocytes constituted 10% of all leukocytes in NSCLC tumors. Neutrophils made up the largest granulocyte population, representing 8.6% of all leukocytes. Basophils and eosinophils constituted 0.4 and 0.3% respectively, while mast cells represented 1.4% of all immune cells in NSCLC tumors (Figure 11). The percentages of mast cells and neutrophils in tumor varied considerably among patients (Figure 12).

### NSCLC Tumors Contain Thirteen Distinct Immune Cell Types

**Figure 13:**
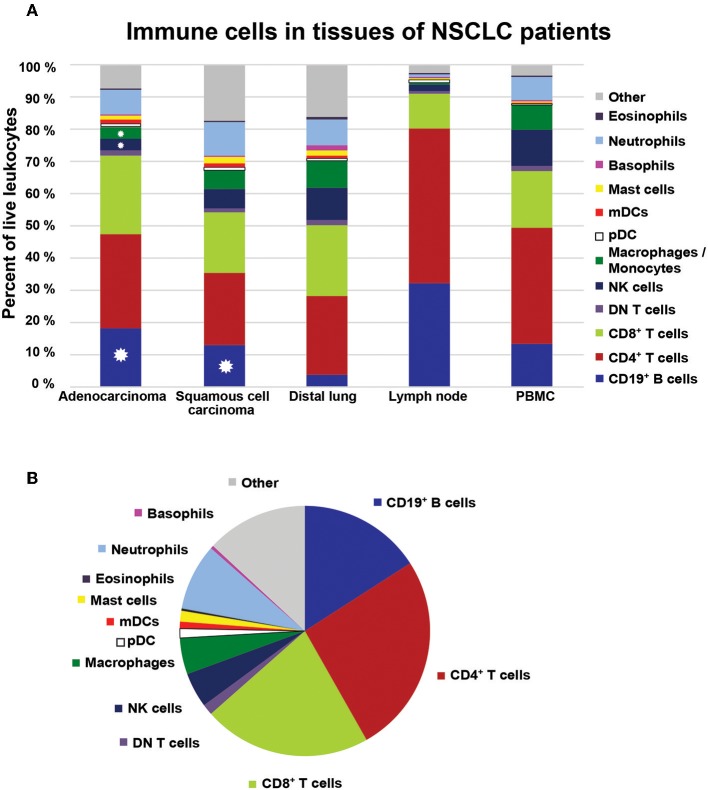
Immune cell composition in NSCLC. **(A)** The bar graph shows the immune cell composition in adenocarcinoma, squamous cell carcinoma, distal lung, regional lymph node, and PBMCs in the cohort of NSCLC patients (*n* = 68). Colors in the bars represent mean values based on data collected for the respective cell populations in the indicated tissue. Distal lung represents pooled data from non-cancerous lung tissue obtained from patients with adenocarcinoma and squamous cell carcinoma. Stars indicate significant differences in percentages of cells between tumor and distal lung calculated by Kruskal-Wallis analysis and Dunn's post-test. **(B)** The immune cell composition in NSCLC tumors illustrated as a pie chart.

All data are summarized in Table 2 and Figure 13. Thirteen distinct immune cell types were identified in NSCLC tumors. T cells were the most frequent immune cells, representing 46.5% of all tumor-infiltrating CD45^+^ leukocytes (Table 2). Three types of T cells were observed in tumor: CD4^+^, CD8^+^, and CD4^−^CD8^−^ DN T cells. CD4^+^ T cells were most abundant (25.9%), closely followed by CD8^+^ T cells (21.7%). B cells were the third most frequent immune cell type in NSCLC tumors (15.9%). Among tumor-infiltrating mononuclear phagocytes, macrophages were the most abundant (4.7%), followed by pDCs (1.2%). Two types of mDCs were identified: CD1c^+^ DCs (0.8%) and CD141^+^ DCs (0.1%). NK cells constituted on average 4.5% of all leukocytes. NSCLC tumors contained few eosinophils (0.3%), basophils (0.4%), and mast cells (1.4%), whereas neutrophils were the most abundant type of granulocytes constituting 8.6% of all leukocytes (Table 2).

**Table 2:** Immune cell types identified in NSCLC tumors^#^.

**Cell population**	**Molecular markers**	**Cell % in adenocarcinoma**	**Cell % in SCC**	**Cell % in NSCLC**
Live Leukocytes	CD45^+^PI^−^	100 (*n* = 32)	100 (*n =* 25)	100 (*n =* 68)
T cells	CD45^+^PI^−^CD3^+^CD19^−^	49.5 (*n =* 25)	41.1 (*n =* 17)	46.5 (*n =* 43)^*^
CD4^+^ T cells	CD45^+^PI^−^CD3^+^CD19^−^CD8^−^CD4^+^	28.6 (*n =* 25)	22.1 (*n =* 17)	25.9 (*n =* 43)^*^
CD8^+^ T cell	CD45^+^PI^−^CD3^+^CD19^−^CD8^+^CD4^−^	23.9 (*n =* 15)	18.2 (*n =* 14)	21.7 (*n =* 30)^*^
CD4^−^ CD8^−^ T cells	CD45^+^PI^−^CD3^+^CD19^−^CD8^−^CD4^−^	1.5 (*n =* 15)	1.3 (*n =* 14)	1.4 (*n =* 30)^*^
B cells	CD45^+^PI^−^CD3^−^CD14^−^CD19^+^	18.0 (*n =* 33)	12.7 (*n =* 22)	15.9 (*n =* 55)
Naive B cells	CD45^+^PI^−^CD3^−^CD14^−^CD19^+^IgM^+^IgD^+^ CD27^−^ CD38^+/−^	1.6 (*n =* 12)	1.6 (*n =* 11)	1.6 (*n =* 23)
CD27^+^CD38^+/−^ B cells	CD45^+^PI^−^CD3^−^CD14^−^CD19^+^IgM^−^IgD^−^ CD27^+^CD38^+/−^	4.1 (*n =* 12)	4.2 (*n =* 11)	4.2 (*n =* 23)
Plasma cells	CD45^+^PI^−^CD3^−^CD14^−^CD19^+^IgM^−^IgD^−^ CD27^+^CD38^++^	0.7 (*n =* 12)	0.9 (*n =* 11)	0.8 (*n =* 23)
IgM^+^IgD^−^ B cells	CD45^+^PI^−^CD3^−^CD14^−^CD19^+^IgM^+^IgD^−^	2.7 (*n =* 12)	1.7 (*n =* 11)	2.2 (*n =* 23)
Macrophages	CD45^+^PI^−^CD19^−^CD14^+^HLA-DR^+^	3.7 (*n =* 18)	5.8 (*n =* 14)	4.7 (*n =* 33)^*^
pDCs	CD45^+^PI^−^CD19^−^CD14^−^HLA-DR^+^CD11c^−^CD123^+^	1.3 (*n =* 16)	1.0 (*n =* 13)	1.2 (*n =* 29)
Classical DCs	CD45^+^PI^−^CD19^−^CD14^−^HLA-DR^+^CD11c^+^	1.7 (*n =* 16)	1.5 (*n =* 13)	1.6 (*n =* 29)
CD1c^+^ DCs	CD45^+^PI^−^CD19^−^CD14^−^ HLA-DR^+^CD11c^+^ CD1c^+^CD141^−^	0.9 (*n =* 16)	0.7 (*n =* 13)	0.8 (*n =* 29)
CD141^+^ DCs	CD45^+^PI^−^CD19^−^CD14^−^ HLA-DR^+^ CD11c^+^CD1c^−^CD141^+^	0.1 (*n =* 16)	0.2 (*n =* 13)	0.1 (*n =* 29)
“DN DCs”	CD45^+^PI^−^CD19^−^CD14^−^HLA-DR^+^CD11c^+^ CD1c^−^CD141^−^	0.5 (*n =* 16)	0.5 (*n =* 13)	0.5 (*n =* 29)
NK cells	CD45^+^PI^−^CD19^−^CD14^−^CD3^−^CD56^+^	3.5 (*n =* 10)	5.8 (*n =* 8)	4.5 (*n =* 18)
CD16^+^ NK cells	CD45^+^PI^−^CD19^−^CD14^−^CD3^−^CD56^+^CD16^+^	1.5 (*n =* 10)	3.3 (*n =* 8)	2.3 (*n =* 18)
CD16^−^ NK cells	CD45^+^PI^−^CD19^−^CD14^−^CD3^−^CD56^+^CD16^−^	2.0 (*n =* 10)	2.5 (*n =* 8)	2.2 (*n =* 18)
Neutrophils	CD45^+^PI^−^CD19^−^CD3^−^C14^−^CD11b^+^CD15^+^CD49d^−^	7.6 (*n =* 11)	10.2 (*n =* 7)	8.6 (*n =* 18)
Basophils	CD45^+^PI^−^CD19^−^CD3^−^C14^−^CD11b^−^FcεR1α^+^CD117^−^CD49d^+^	0.5 (*n =* 11)	0.4 (*n =* 7)	0.4 (*n =* 18)
Eosinophils	CD45^+^PI^−^CD19^−^CD3^−^C14^−^CD11b^+^CD15^+^CD49d^+^	0.2 (*n =* 11)	0.4 (*n =* 7)	0.3 (*n =* 18)
Mast cells	CD45^+^PI^−^CD19^−^CD3^−^C14^−^CD11b^−^FcεR1α^+^CD117^+^CD49d^+^	1.0 (*n =* 11)	2.0 (*n =* 7)	1.4 (*n =* 18)

# *Numbers indicate mean percentages of all live leukocytes (defined as CD45^+^PI^−^ cells) for all NSCLC tumor samples analyzed for each immune cell type. n = number of patients analyzed for each cell type*.

* *Includes one large cell carcinoma tumor*.

Comparison of the leukocyte composition in tumor vs. distal lung showed that there was a significant increase in the fraction of infiltrating B cells for both adenocarcinoma and squamous cell carcinoma. In contrast, the percentages of macrophages and NK cells were lower in NSCLC tumors compared to distal lung (Figure 13). The remaining cell populations showed similar percentages in tumors compared to distal lung. Steroid treatment (which was used by 50% of the patients with CODP included in this study) did not seem to affect the immune cell composition in NSCLC tumors (Supplementary Figure 18). Statistical analysis revealed several correlations between immune cell types in NSCLC tumors (Supplementary Table 7; Supplementary Figure 19). For example, the percentage of macrophages in tumor was negatively correlated with the percentage of B cells (Supplementary Figure 19C). Furthermore, several granulocyte combinations (neutrophils/eosinophils, eosinophils/mast cells, neutrophils/mast cells) were positively correlated, suggesting that the microenvironment of some NSCLC tumors may be prone to attract granulocytes in general (granulocytic inflammation) (Supplementary Table 7; Supplementary Figure 19I–K). The gating strategy used to identify immune cells in lymph nodes is shown in Supplementary Figures 20–24.

When we summed up the average values for each immune cell type identified, there was a lacking fraction, denoted “other” in Figures 13A,B, to achieve 100% of CD45^+^ leukocytes. This “other” population of potentially unidentified immune cells appeared quite large, in particular in squamous cell carcinoma tumors (Figure 13A). However, we suspected that such a large population of unidentified cells was not real but rather the result of an artifact due to the usage of five different panels to identify all types of immune cells. To clarify this, we set up a single multicolor flow cytometry panel (Supplementary Table 6) covering most immune cell types identified: T cells, B cells, macrophages, NK cells, and granulocytes. By using this single panel to analyze samples from two additional NSCLC patients, we found that we had successfully identified 95.5 and 96.2% of all CD45^+^ leukocytes in squamous cell carcinoma and adenocarcinoma, respectively (Supplementary Figures 25, 26). The remaining 4–5% of unidentified leukocytes may potentially consist of DCs or innate lymphoid cells which were not stained by this panel. Thus, we can conclude that >95% of all CD45^+^ immune cells in NSCLC tumors were fully characterized and that 13 distinct immune cell types were identified.

## Discussion

In this study, we characterized the immune cell composition in tumor, distal lung, lymph node, and blood from 68 NSCLC patients. Samples from tumor, distal lung tissue and lymph nodes were processed with the same protocol using enzymatic digestion to allow comparison between tissues. In contrast, PBMCs were isolated from the blood of patients using a different protocol (a density gradient), because PBMCs were essentially used as a positive control for the staining and cell identification and not for direct comparison with the three tissues investigated. We found that adenocarcinoma tumors had a higher percentage of CD45^+^ leukocytes compared to distal lung. A similar trend was observed for squamous cell carcinoma tumors, although the *p*-value did not reach statistical significance. These data are in accordance with previous findings (28, 30) and confirm that the microenvironment of NSCLC tumors is rich in immune cells. Thirteen distinct immune cell types were identified in NSCLC tumors which altogether comprise >95% of all CD45^+^ leukocytes: CD4^+^ T cells, CD8^+^ T cells, DN T cells, B cells, macrophages, pDCs, CD1c^+^ mDCs, CD141^+^ mDCs, NK cells, neutrophils, basophils, eosinophils, and mast cells.

T cells were found to dominate the immune landscape (on average 46.5% of all CD45^+^ cells). The observed high percentage of T cells among tumor-infiltrating leukocytes in NSCLC is in accordance with previous reports (28–30, 49, 50). However, we did not observe an increased frequency of T cells in tumors as compared to distal lung as reported by others (28–30, 49). It should be noted that our gating strategy did not allow distinction between αβ T cells (presumably the large majority of CD3^+^ T cells in NSCLC) and γδ T cells, which have also been reported in lung tumors (51). The majority of CD4^+^ and CD8^+^ T cells in tumor exhibited a CD45RA^−^CD45RO^+^ effector/memory phenotype, as could be expected from T cells present in non-lymphoid organs. Interestingly, small populations of CD45RA^+^CD45RO^−^ CD4^+^ T cells and CD8^+^ T cells were also observed in NSCLC tumors. Immunofluorescence staining showed that these CD45RA^+^CD3^+^ T cells were located both in tumor stroma (i.e., between tumor cell areas) and at the tumor periphery in TLS. CD45RA^+^CD3^+^ T cells in TLS are likely to be *bona fide* naïve T cells (35). In contrast, naïve T cells are not expected to reside in tumor stroma, i.e., outside secondary or tertiary lymphoid organs. Therefore, it is likely that the CD45RA^+^CD3^+^ T cells in tumor stroma consist of CD45RA^+^ effector memory CD8^+^ T cells, the so called T_EMRA_ cells (33, 34). T cell presence in tumor has been reported to be positively correlated with survival in several types of cancers including NSCLC (52, 53). Therefore, the presence and the activation status of T cells may be useful as a marker of disease outcome in NSCLC.

The observed increase in percentage of B cells in NSCLC tumors compared to distal lung tissue is in accordance with previous reports (28–30). However, in a previous study which included a large number of patients (*n* = 73), B cells represented only 4.4% of the CD45^+^ cells in NSCLC tumors (24), which is about 3.5 times less than the percentage (15.9%) found in our cohort (24). This large difference in B cell proportions may be due to differences in how the tumor tissue samples were collected because B cells are present mostly at the tumor periphery (54), where they cluster in TLS together with DCs and T cells (24). B cells have been reported to be a good marker of clinical outcome, both in combination with DCs or T cells, as well as on their own (24, 37, 55).

Three types of DCs, namely pDCs (CD123^+^), CD141^+^ DCs, and CD1c^+^ DCs were identified in NSCLC tumors, by use of established markers. CD141^+^ DCs have previously been reported to be decreased in tumors compared to non-cancerous lung tissue (49). Our data did not confirm such a decrease, although we analyzed a rather large cohort of patients for DC subsets (*n* = 29). It is likely that other DC subsets are present in NSCLC tumors but were not detected by our gating strategy. For example, HLA-DR^+^CD11c^+^CD1c^+^CD14^+^ inflammatory DCs (56) were not examined because our gating strategy used CD14 as a specific marker for macrophages and all DCs were considered to be CD14^−^. In addition, we excluded a few DCs being CD141^+^CD1c^+^, a subset that has been observed in other studies (57, 58). Ontogenetic alignments with mouse conventional DCs (cDC) have classified human CD141^+^ DCs and CD1c^+^ DC as cDC1 and cDC2, respectively (59). DCs of distinct cellular origin display different functions. Whereas, pDCs have the potential to enhance antitumor immunity by production of type I IFNs (46, 60), cDC1 and cDC2 are considered to be specialized in antigen presentation and activation of naïve T cells. CD1c^+^ DCs (cDC2) were found to be much more frequent (0.8% of all leukocytes) than CD141^+^ DCs (cDC1, 0.1%) in NSCLC tumors. This observation is consistent with cDC2 playing the central role of “guardians of the mucosa” in the lungs as suggested by a recent report (58).

The percentages of macrophages were reduced in tumors (adenocarcinoma and all NSCLC) compared to distal lung in accordance with a previous report (30). Notably, Kargl et al. reported that macrophages represented on average 15% of all CD45^+^ cells in NSCLC tumors, which is three times higher than in our study (4.7%) (30). We defined macrophages as CD45^+^CD3^−^CD19^−^CD14^+^HLA-DR^+^ cells, whereas Kargl et al. used CD68^+^ to identify macrophages from live CD45^+^ leukocytes. The discrepancy in gating strategy may at least partly explain the observed difference in proportions of macrophages. Our study shows that there is an increase of proportion of macrophages with high HLA-DR expression in tumor compared to distal lung. These HLA-DR^high^ macrophages may potentially represent IFN-γ activated macrophages with antitumor activity (42–46), since IFN-γ has been shown to specifically induce HLA-DR expression on macrophages (47). Macrophages may contribute to the efficacy of PD-1 immune checkpoint blockade in NSCLC as suggested by a recent study reporting a large number of infiltrating macrophages (and lymphocytes) in responding patients (61).

Our analysis of NK cells identified both CD16^+^ and CD16^−^ NK cells in NSCLC and revealed a reduction of NK cells in tumor compared to distal lung, which is in accordance with previous reports (29, 30). The reduced percentage of NK cells in tumor was mostly due to lower levels of the CD16^+^ subset of NK cells. Although less frequent than in normal lung tissue, NK cells represented a significant fraction (4.5%) of all immune cells in NSCLC tumors. NK cells may potentially participate in antitumor immunity both by killing cancer cells and by secreting IFN-γ. However, it has been reported that NK cells in NSCLC tumors may have a reduced ability both to kill target cells and produce IFN-γ (62, 63).

Granulocytes were shown to represent ~10% of all immune cells in NSCLC tumors. The largest population was the neutrophils (8.6%) followed by mast cells (1.4%), basophils (0.4%), and eosinophils (0.3%). A high frequency of neutrophils in NSCLC tumors has been previously shown (30). In fact, it was reported that neutrophils were the most abundant immune cell type in NSCLC tumors, accounting for nearly 20% of all CD45^+^ cells (30). Although our analysis also showed high numbers of neutrophils in tumors, we found a percentage of neutrophils (8.6% of CD45^+^ cells) ~50% lower than the one reported by Kargl et al. (30). This difference may partially be explained by the different markers used and by differences in gating strategy. Even though there is a discrepancy in the percentage of neutrophils in different studies, neutrophils clearly represent a significant portion of immune cells in both lung adenocarcinoma and squamous cell carcinoma. Despite this fact, very little is currently known about the role of neutrophils in lung cancer. It has been suggested that neutrophils should be divided into N1 and N2 phenotypes with antitumor and protumor activity, respectively (64, 65). A few studies indicated that neutrophils might play a detrimental role in NSCLC (66, 67).

The recent success of cancer immunotherapy in clinical trials has revolutionized cancer treatment and put the immune system in focus as a prognostic marker and a target for novel therapy. However, it remains largely unknown why some patients respond to immunotherapy while others do not. A detailed characterization of the immune cell composition in tumors is likely to be fundamental for the development of novel therapeutic agents, as well as prognostic and predictive biomarkers.

## Author Contributions

BS performed experiments, analyzed the data, prepared the figures, conducted the statistical analysis, and wrote the first draft of manuscript. HB and RS performed experiments, analyzed data, and prepared figures. HB and EB contributed to the conception and design of the study and helped with the experiments. AF and CH conducted the immunohistochemistry/immunofluorescence analysis. EM and KB helped with the experiments. PW recruited patients and provided biopsy samples. ÅH and OB organized the biobank and helped providing clinical data. IØ provided supervision, discussed the results and wrote sections of the manuscript. AC designed and supervised the study, evaluated the experiments, and contributed to writing the manuscript. All authors read and approved the final version of the manuscript.

### Conflict of Interest Statement

The authors declare that the research was conducted in the absence of any commercial or financial relationships that could be construed as a potential conflict of interest.

## Abbreviations

CD: cluster of differentiation
COPD: chronic obstructive pulmonary disease
CTLA-4: cytotoxic T-lymphocyte-associated protein 4
cDC: conventional dendritic cell
DC: dendritic cell
DN: double negative
FBS: fetal bovine serum
FFPE, formalin fixed: paraffin-embedded
FSC-A: forward scatter area
FSC-H: forward scatter height
HLA-DR: human leukocyte antigen–antigen D related
IFN: interferon
Ig: immunoglobulin
LN: lymph node
mDC: myeloid dendritic cell
NK: natural killer
NSCLC: non-small cell lung cancer
PBMC: peripheral blood mononuclear cell
PD-1: programmed cell death protein 1
pDC: plasmacytoid dendritic cell
PI: propidium iodide
SSC-A: side scatter area
SSC-H: side scatter height
TLS: tertiary lymphoid structures
TNM, tumor: lymph node and metastasis-based cancer staging.
