# Supplementary material for: Immune Cell Composition in Human Non-small Cell Lung Cancer
Source: Front Immunol. 2019 Feb 1;9:3101. doi: 10.3389/fimmu.2018.03101 (PMC6367276; doi:10.3389/fimmu.2018.03101)
Supplement: Supplementary file 1 [file Data_Sheet_1.pdf]

Supplementary Material

Immune Cell Composition in Human Non-Small Cell Lung Cancer

Branislava Stankovic, Heidi Anine Korsmo Bjørhovde, Renate Skarshaug, Henrik Aamodt, Astri Frafjord, Elisabeth Müller, Clara Hammarström, Kahsai Beraki, Espen S. Bækkevold, Per Reidar Woldbæk, Åslaug Helland, Odd Terje Brustugun, Inger Øynebråten, and Alexandre Corthay

Table of contents:

Supplementary Table 1. Antibody mix used to assess T cells in NSCLC patients.....2
Supplementary Table 2. Antibody mix used to assess B cells in NSCLC patients.....2
Supplementary Table 3. Antibody mix used to assess APCs in NSCLC patients.....2
Supplementary Table 4. Antibody mix used to assess NK cells in NSCLC patients.....2
Supplementary Table 5. Antibody mix used to assess granulocyte populations in NSCLC patients.....3
Supplementary Table 6. Antibody mix used to assess "Other" populations in NSCLC patients.....3
Supplementary Table 7. Correlation between immune cell infiltrates in NSCLC tumor.....4
Supplementary Figure 1. No influence of steroid use and COPD on immune cell infiltration in NSCLC.....5
Supplementary Figure 2. Flow cytometry analysis of T cells in PBMCs from NSCLC patients.....6
Supplementary Figure 3. Immunofluorescent staining of CD45RA$^+$CD3$^+$ cells in NSCLC tumor tissue.....7
Supplementary Figure 4. Percentages of T cells in different tissues from NSCLC patients.....8
Supplementary Figure 5. Percentages of T cells in different stages of NSCLC and in patients with different smoking histories.....9
Supplementary Figure 6. Flow cytometry analysis of B cell sub-populations in PBMCs from NSCLC patients.....10
Supplementary Figure 7. Percentages of B cells in adenocarcinoma and squamous cell carcinoma.....11
Supplementary Figure 8. Percentages of B cells in different stages of NSCLC.....12
Supplementary Figure 9. Percentages of B cells in NSCLC patients with different smoking histories.....13
Supplementary Figure 10. Expression of CD11b and CD11c on macrophages and DCs in NSCLC tumor.....14
Supplementary Figure 11. Flow cytometry of monocytes and DCs in PBMCs from NSCLC patients.....15
Supplementary Figure 12. Percentages of DC subsets and macrophages in different stages of NSCLC.....16
Supplementary Figure 13. Percentages of DC subsets in NSCLC adenocarcinoma and squamous cell carcinoma.....17
Supplementary Figure 14. Proportions of different DC populations in NSCLC.....18
Supplementary Figure 15. Percentages of DC subsets in different tissues from NSCLC patients.....18
Supplementary Figure 16. Flow cytometry analysis of NK cells in PBMCs from NSCLC patients.....19
Supplementary Figure 17. Flow cytometry analysis of granulocyte populations in PBMCs from NSCLC patients.....20
Supplementary Figure 18. Immune cell infiltrates in NSCLC tumor from patients grouped based on steroid treatment.....21
Supplementary Figure 19. Linear regression analysis of immune cell infiltrates in NSCLC tumor.....22
Supplementary Figure 20. Flow cytometry analysis of T cells in lymph node from NSCLC patients.....23
Supplementary Figure 21. Flow cytometry analysis of B cells in lymph node from NSCLC patients.....24
Supplementary Figure 22. Flow cytometry analysis of macrophages and DCs in lymph node of NSCLC patients.....25
Supplementary Figure 23. Flow cytometry analysis of NK cells in lymph node from NSCLC patients.....26
Supplementary Figure 24. Flow cytometry analysis of granulocyte populations in lymph node from NSCLC patients.....27
Supplementary Figure 25. Flow cytometry analysis of "Other" population in squamous cell carcinoma.....28
Supplementary Figure 26. Flow cytometry analysis of "Other" population in adenocarcinoma.....29

Supplementary Table 1. Antibody mix used to assess T cells in NSCLC patients

Specificity	Fluorophore	Clone	Volume	Manufacturer	Cat. No
CD3	A488	UCHT1	2.5 µl	Biolegend	300415
CD4	PE	OKT4	2.5 µl	Biolegend	317410
CD8	APC/Cy7	SK1	2.5 µl	Biolegend	344714
CD19	A700	HIB19	1 µl	Biolegend	302226
CD45	Pacific Blue	HI30	2.5 µl	Biolegend	304029
CD45RO	APC	UCHL1	2.5 µl	Biolegend	304210
CD45RA	PE/Cy7	HI100	2.5 µl	Biolegend	304126

Supplementary Table 2. Antibody mix used to assess B cells in NSCLC patients

Specificity	Fluorophore	Clone	Volume	Manufacturer	Cat. No
CD14	APC/Cy7	HCD14	2.5 µl	Biolegend	325620
CD38	APC	HB-7	2.5 µl	Biolegend	356606
CD19	PE/Cy7	HIB19	2.5 µl	Biolegend	302216
CD27	PE	O323	2.5 µl	Biolegend	302808
IgD	A488	IA6-2	2.5 µl	Biolegend	348216
CD3	A700	UCHT1	1 µl	Biolegend	300424
IgM	BV605	MHM-88	2.5 µl	Biolegend	314524
CD45	Pacific Blue	HI30	2.5 µl	Biolegend	304029

Supplementary Table 3. Antibody mix used to assess APCs in NSCLC patients

Specificity	Fluorophore	Clone	Volume	Manufacturer	Cat. No
CD11c	A488	3.9	2.5 µl	Biolegend	301618
CD14	APC/Cy7	HCD14	2.5 µl	Biolegend	325620
HLA-DR	PE/Cy7	L243	2.5 µl	Biolegend	307616
CD141	APC	AD5-14H12	2.5 µl	Miltenyi Biotec	130-090-907
CD1c	PE	L161	2.5 µl	Biolegend	331506
CD123	BV605	6H6	2.5 µl	Biolegend	306025
CD19	A700	HIB19	1 µl	Biolegend	302226
CD45	Pacific Blue	HI30	2.5 µl	Biolegend	304029

Supplementary Table 4. Antibody mix used to assess NK cells in NSCLC patients

Specificity	Fluorophore	Clone	Volume	Manufacturer	Cat. No
CD3	A488	UCHT1	2.5 µl	Biolegend	300415
CD19	BV605	HIB19	2.5 µl	Biolegend	302244
CD56	PE	HCD56	2.5 µl	Biolegend	318305
CD16	APC	3G8	2.5 µl	Biolegend	302012
CD14	APC/Cy7	HCD14	2.5 µl	Biolegend	325620
CD45	Pacific Blue	HI30	2.5 µl	Biolegend	304029

Supplementary Table 5. Antibody mix used to assess granulocyte populations in NSCLC patients

Specificity	Fluorophore	Clone	Volume	Manufacturer	Cat. No
CD45	Pacific Blue	HI30	2.5 µl	Biolegend	304029
HLA DR	BV605	l243	2.5 µl	Biolegend	307640
CD19	A700	HIB19	1 µl	Biolegend	302226
CD3	A700	UCHT1	1 µl	Biolegend	300424
CD14	APC/Cy7	HCD14	2.5 µl	Biolegend	325620
CD15	A488	W6D3	2.5 µl	Biolegend	323010
CD11b	BV510	ICRF44	2.5 µl	Biolegend	301334
CD49d	PE	9F10	2.5 µl	Biolegend	304304
CD117	PE/Cy7	104D2	2.5 µl	Biolegend	313212
FceR1a	APC	AER-37	2.5 µl	eBioscience	17-5899-42

Supplementary Table 6. Antibody mix used to assess "Other" populations in NSCLC patients

Specificity	Fluorophore	Clone	Volume	Manufacturer	Cat. No
CD45	Pacific Blue	HI30	2.5 µl	Biolegend	304029
CD14	APC/Cy7	HCD14	2.5 µl	Biolegend	325620
HLA-DR	PE/Cy7	L243	2.5 µl	Biolegend	307616
CD19	BV605	HIB19	2.5 µl	Biolegend	302244
CD3	A700	UCHT1	1 µl	Biolegend	300424
CD56	PE	HCD56	2.5 µl	Biolegend	318305
FcεR1a	APC	AER-37 (CRA1)	2.5 µl	eBioscience	17-5899-42
CD15	A488	W6D3	2.5 µl	Biolegend	323010
CD11b	BV510	ICRF44	2.5 µl	Biolegend	301334

Supplementary Table 7. Correlation between immune cell infiltrates in NSCLC tumor#

	Correlations		$CD3^+$ T cells	$CD19^+$ B cells	$CD56^+$ NK cells	$CD141^+$ DCs	$CD1c^+$ DCs	DN DCs	Macrophages	Eosinophils	Neutrophils	Basophils	Mast cells
Spearman's rho	$CD3^+$ T cells	Correlation	1.000	-0.140	-0.018	0.288	0.034	0.023	-0.148	-0.285	0.297	0.673*	0.248
		P value	.	0.361	0.931	0.104	0.848	0.907	0.389	0.425	0.405	0.033	0.489
		N ¤	52	45	25	33	34	29	36	10	10	10	10
	$CD19^+$ B cells	Correlation		1.000	-0.188	-0.313	-0.506**	-0.287	- 0.604**	0.505	0.056	0.148	0.492
		P value		.	0.357	0.086	0.003	0.147	0.000	0.055	0.844	0.599	0.063
		N		57	26	31	32	27	34	15	15	15	15
	$CD56^+$ NK cells	Correlation			1.000	0.632**	0.390	0.501*	0.349	-0.046	0.165	0.666**	-0.178
		P value			.	0.002	0.080	0.021	0.120	0.876	0.573	0.009	0.543
		N			30	21	21	21	21	14	14	14	14
	$CD141^+$ DCs	Correlation				1.000	0.344	0.277	0.094	-0.455	0.245	0.329	-0.308
		P value				.	0.050	0.132	0.593	0.138	0.443	0.297	0.331
		N				35	33	31	35	12	12	12	12
	$CD1c^+$ DCs	Correlation					1.000	0.486**	0.368*	0.042	-0.046	0.214	-0.014
		P value					.	0.006	0.027	0.897	0.888	0.504	0.965
		N					36	31	36	12	12	12	12
	DN DCs	Correlation						1.000	0.307	-0.210	0.186	0.515	0.249
		P value						.	0.093	0.512	0.564	0.087	0.436
		N						31	31	12	12	12	12
	Macrophages	Correlation							1.000	-0.084	-0.252	0.049	-0.357
		P value							.	0.795	0.430	0.880	0.255
		N							38	12	12	12	12
	Eosinophils	Correlation								1.000	0.560*	-0.149	0.562*
		P value								.	0.016	0.554	0.015
		N								18	18	18	18
	Neutrophils	Correlation									1.000	0.314	0.520*
		P value									.	0.205	0.027
		N									18	18	18
	Basophils	Correlation										1.000	0.253
		P value										.	0.311
		N										18	18
	Mast cells	Correlation											1.000
		P value											.
		N											18

Spearman's rho in IBM SPSS version 25 was used to assess the strength of association between types of immune cells in NSCLC tumor. Value of 1 or -1 represents perfect correlation, whereas 0 indicates no association. Correlations that were considered significant are highlighted in yellow. Percentages of immune cells calculated from the live leukocyte population were used in the analysis.

¤ N=Number of NSCLC patients. All analyzed NSCLC patient tumors were included in the analysis.

* $p<0.05$ level (2-tailed)

** $p<0.01$ level (2-tailed)

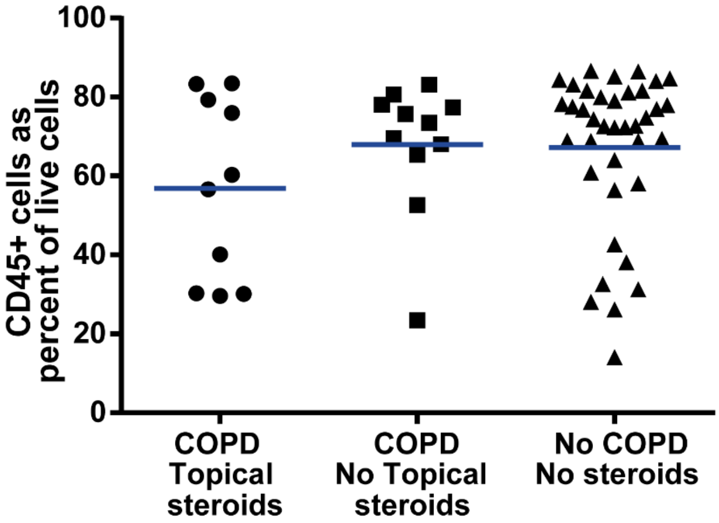

Supplementary Figure 1. No influence of steroid use and chronic obstructive pulmonary disease (COPD) on immune cell infiltration in NSCLC. The figure shows $CD45^+$ leukocyte infiltration in tumor tissue from NSCLC patients with COPD who used topical steroids, NSCLC patients with COPD who did not use topical steroids, and NSCLC patients who did not exhibit COPD and did not use steroids. Leukocytes ($CD45^+$ cells) are presented in percent of all live (PI^-) cells. Each symbol represents data from one patient. Blue lines indicate mean values, and differences between group means were tested using Kruskal-Wallis test with Dunn's post-test. No significant p values were observed.

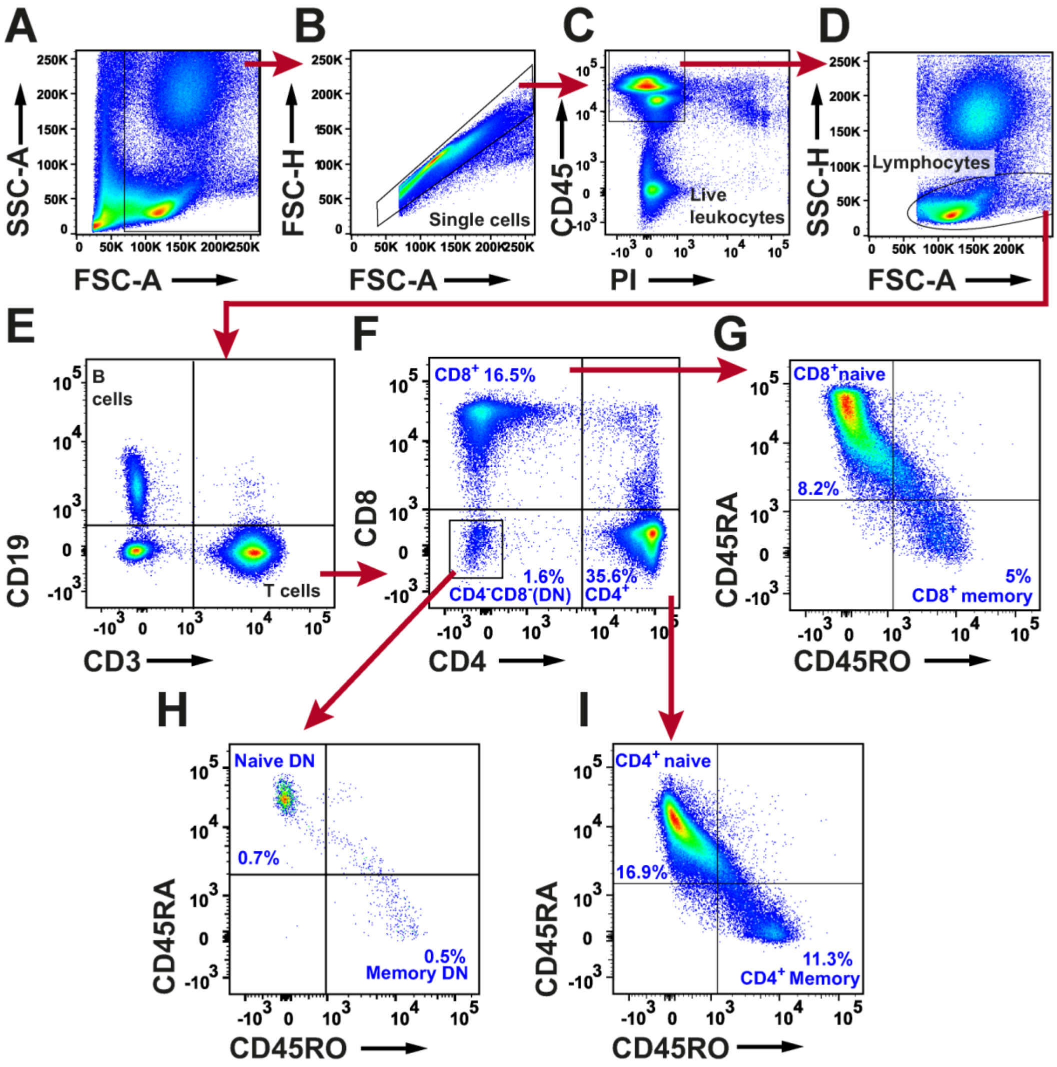

Supplementary Figure 2. Flow cytometry analysis of T cells in PBMCs from NSCLC patients. (A) The FSC-A versus SSC-A plot was used to gate nucleated cells. **(B)** Single cells were gated in an FSC-A versus FSC-H plot. **(C)** Live leukocytes were defined as $CD45^+PI^-$ cells. **(D)** The lymphocyte gate. **(E)** T cells are defined as $CD19^{--}$ $CD3^+$ cells which were divided in **(F)** $CD4^+$ population, $CD8^+$ population, and $CD4^-CD8^-$ population. Each subset was examined for $CD45RA^+CD45RO^-$ naive phenotype and $CD45RA^-CD45RO^+$ memory phenotype. **(G)** Naive/memory phenotyping of $CD8^+$ T cells. **(H)** Naive/memory phenotyping of $CD4^-CD8^-$ T cells. (**I**) Naive/memory phenotyping of $CD4^+$ T cells. The numbers indicated for all populations represent mean percent calculated from the total number of live leukocytes for 26 NSCLC patients (adenocarcinoma n=15, and squamous cell carcinoma n=11). DN, $CD4^-CD8^-$ double negative T cells.

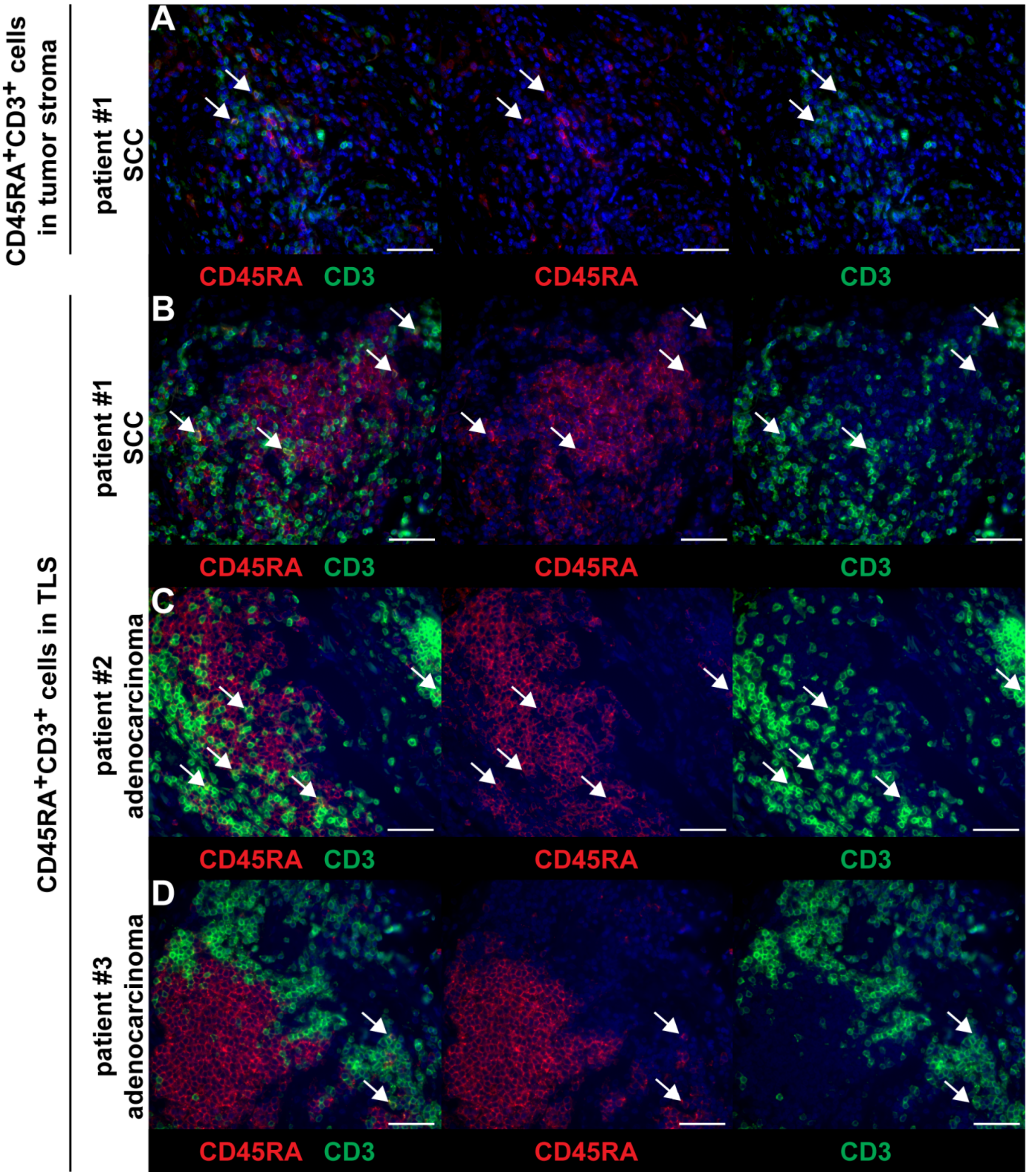

Supplementary Figure 3. Immunofluorescent staining of CD45RA⁺CD3⁺ cells in NSCLC tumor tissue.

(A-D) Representative images of paraffin-embedded sections from tumor tissue of 1 patient with lung squamous cell carcinoma and 2 patients with lung adenocarcinoma stained with anti-CD3 monoclonal antibody clone 2GV6 (green) and anti-CD45RA monoclonal antibody clone 158-4D3 (red). The sections were incubated with a mixture of the anti-CD45RA and anti-CD3 antibodies overnight at 4 °C, followed by incubation with a mixture of Alexa Fluor 488-labeled goat anti-rabbit IgG and Alexa Fluor 555-labeled goat anti-mouse IgG2a for 2 h at room temperature. Some double positive $CD3^+CD45RA^+$ T cells (white arrows) were observed both in **(A)** tumor stroma and **(B-D)** tertiary lymphoid structures (TLS). Double positive $CD3^+CD45RA^+$ T cells in tumor stroma are presumably T_{EMRA} cells, whereas $CD3^+CD45RA^+$ T cells in TLS more likely represent naive T cells. Similar results were observed in tissue from all the investigated NSCLC patients (n=5). Histological subtypes for each patient are indicated. SCC, squamous cell carcinoma. TLS, tertiary lymphoid structures. Original magnification, 400x. Scale bars, 100 µm.

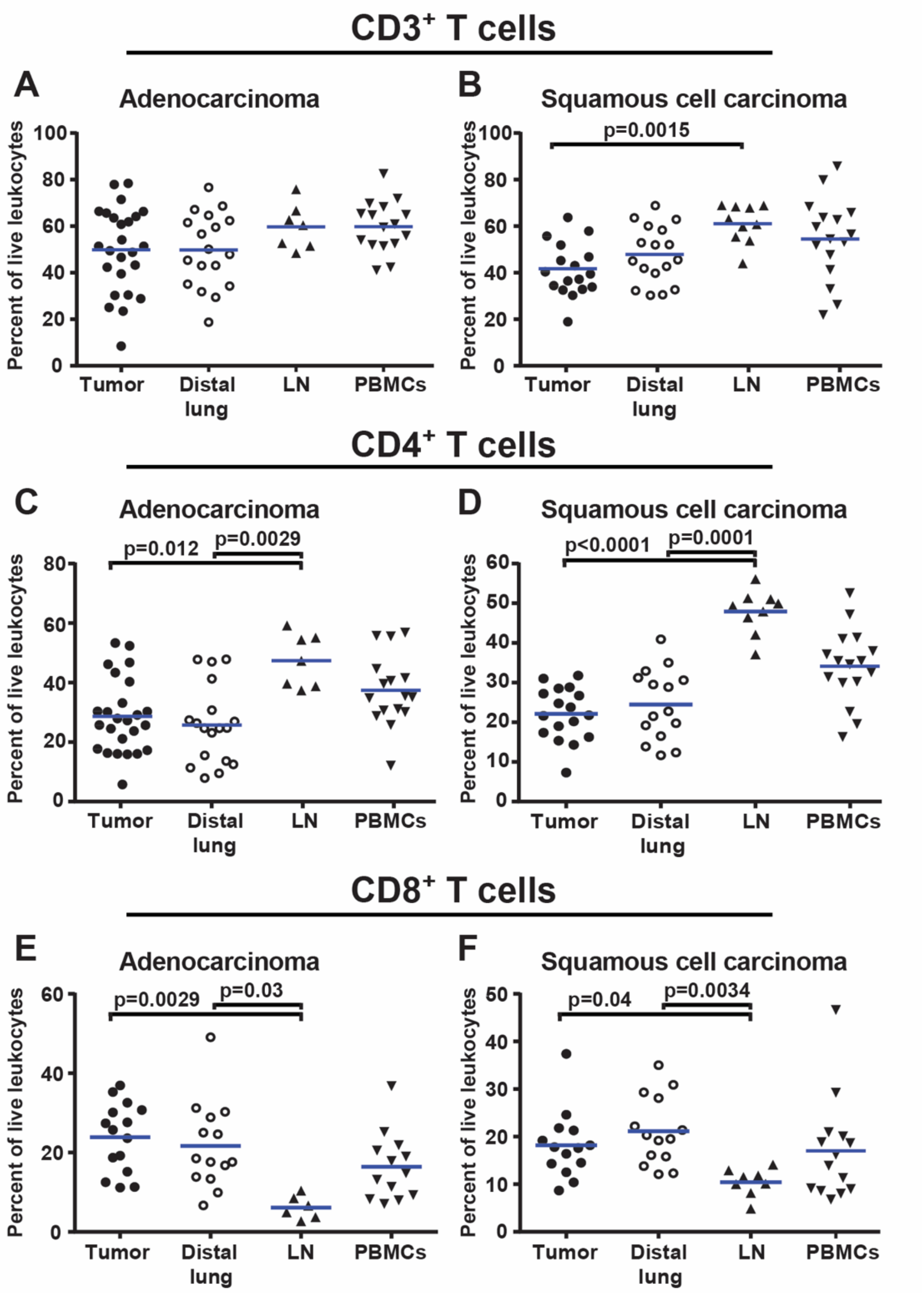

Supplementary Figure 4. Percentages of T cells in different tissues from NSCLC patients. (A,B) Comparison of $CD3^+$ T cell percentages in tissues from different anatomical locations of patients diagnosed with **(A)** adenocarcinoma and **(B)** squamous cell carcinoma. **(C,D)** Percentages of $CD4^+$ T cells in different tissues from patients with **(C)** adenocarcinoma and **(D)** squamous cell carcinoma. **(E,F)** Comparison of $CD8^+$ T cell percentages in **(E)** adenocarcinoma and **(F)** squamous cell carcinoma. The percentages were calculated from the total number of live leukocytes ($CD45^+PI^-$), and each symbol represents data from one patient. Blue lines indicate mean values. Kruskal-Wallis test and Dunn's post-test were used to detect differences between tumor, distal lung, and lymph node (LN).

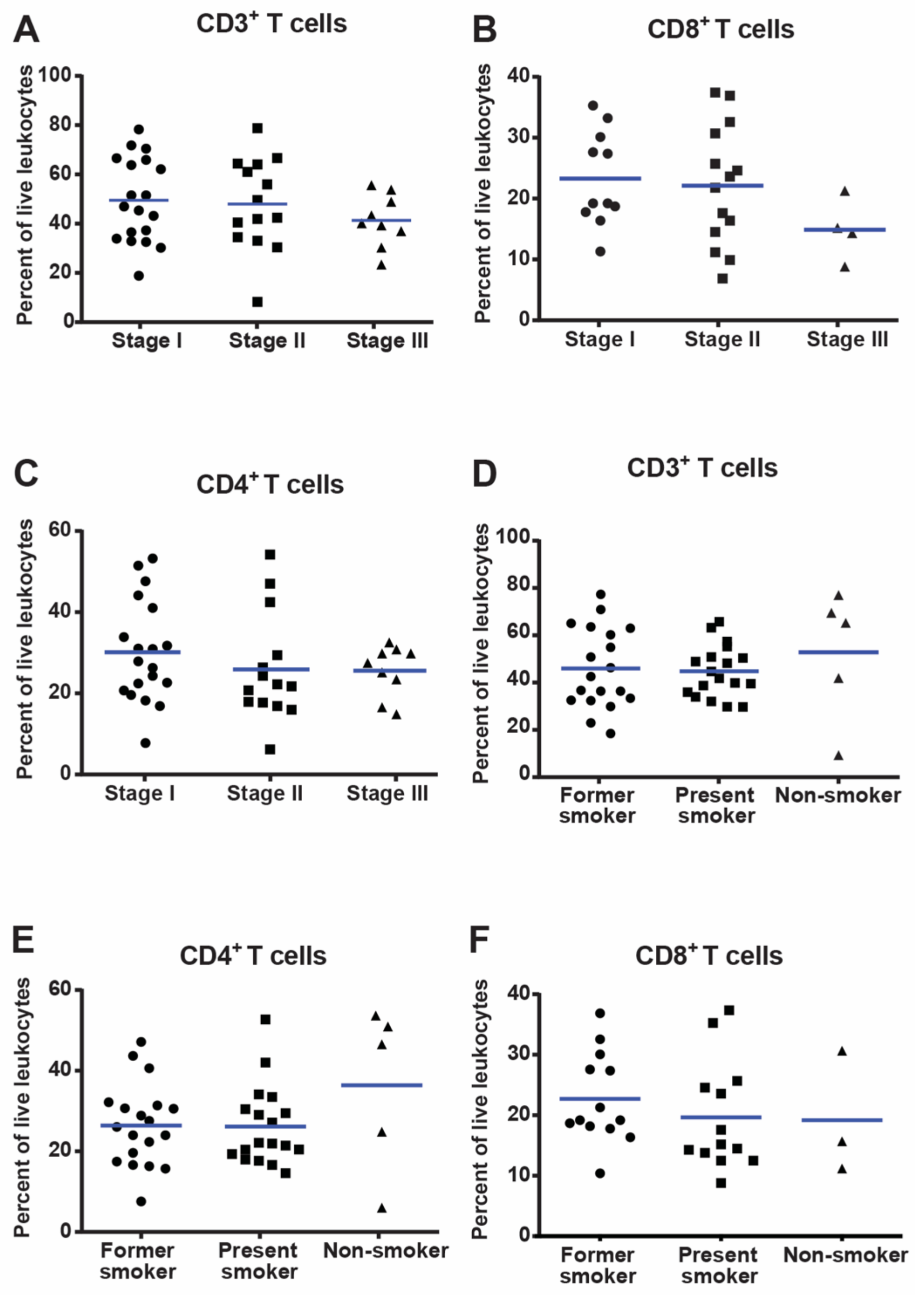

Supplementary Figure 5. Percentages of T cells in different stages of NSCLC and in patients with different smoking histories. (A-C) T cells as percentage of live leukocytes ($CD45^{+}PI^{-}$) in NSCLC tumor stages I, II and III: **(A)** $CD3^{+}$ T cells, **(B)** $CD8^{+}$ T cells, and **(C)** $CD4^{+}$ T cells. **(D-F)** T cells as percentage of live leukocytes ($CD45^{+}PI^{-}$) in tumor from NSCLC patients with different smoking histories: **(D)** $CD3^{+}$ T cells, **(E)** $CD4^{+}$ T cells, and **(F)** $CD8^{+}$ T cells. The group former smoker includes patients who stopped smoking at the latest 6 months before surgery; present smoker smoked at the time of surgery or at least 6 months prior to surgery; and non-smoker never smoked. Each symbol represents data from one patient, and blue lines indicate mean values. The gating strategy is shown in Figure 2. Kruskal-Wallis test and Dunn's post-test were used to detect differences between group means. No significant p values were obtained.

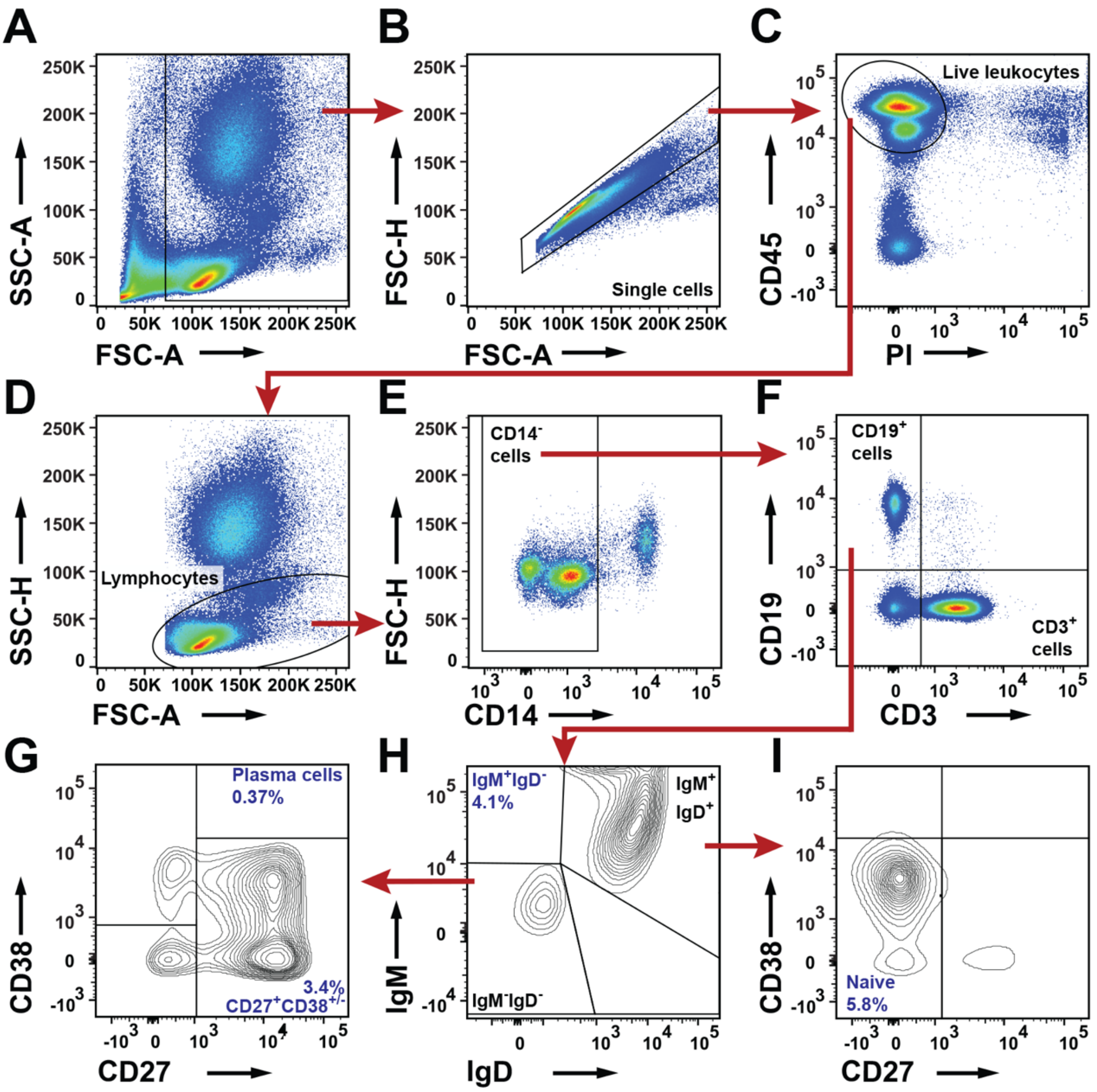

Supplementary Figure 6. Flow cytometry analysis of B cell sub-populations in PBMCs from NSCLC patients. (A) FSC-A and SSC-A were used to exclude debris and gate nucleated cells. **(B)** The FSC-A versus FSC-H plot was used to gate single cells and exclude doublets. **(C)** Live leukocytes were defined as $CD45^{+}PI^{-}$. **(D)** The lymphocyte gate in FSC and SSC plot. **(E)** Gate excluding $CD14^{+}$ monocytes. **(F)** The B cell gate defining all B cells as $CD19^{+}$ and $CD3^{-}$. **(G-I)** Characterization of B cell sub-populations. **(G)** $IgM^{-}IgD^{-}$ B cells could be divided into $CD27^{+}CD38^{+/-}$ B cells and $CD27^{+}CD38^{++}$ plasma cells. **(H)** $CD19^{+}CD3^{-}$ were separated into three populations based on IgM and IgD expression. **(I)** Naive B cells are defined as $CD27^{-}CD38^{-/+}$. Percentages shown in the figure are calculated from the total number of live leukocytes and represent mean values for PBMCs of 25 NSCLC patients (adenocarcinoma n=15, and squamous cell carcinoma n=10).

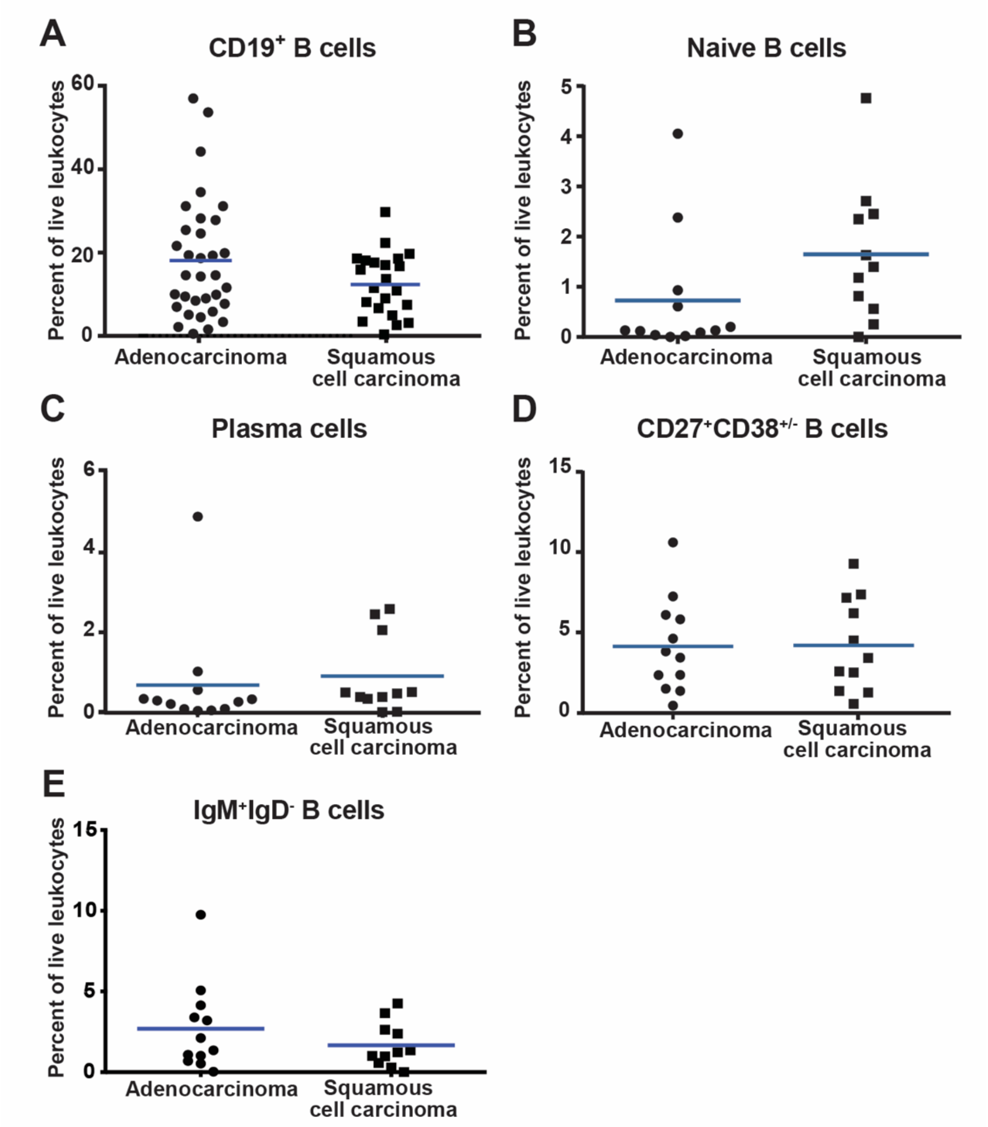

Supplementary Figure 7. Percentages of B cells in adenocarcinoma and squamous cell carcinoma. (A-E) Percentages of $CD19^+$ B cells and B cell sub-populations in NSCLC tumor tissue of adenocarcinoma and squamous cell carcinoma. **(A)** Percentages of $CD19^+$ B cells, **(B)** naive B cells, **(C)** plasma cells, **(D)** $CD27^+CD38^{+/-}$ B cells, and **(E)** IgM^+IgD^- B cells. The percentages are calculated from the population of live leukocytes ($CD45^+PI^-$), and the gating strategy is shown in Figure 4. Each symbol represents data from one patient, and blue lines indicate mean values. Kruskal-Wallis test and Dunn's post-test were used to detect differences between groups. No significant p values were obtained.

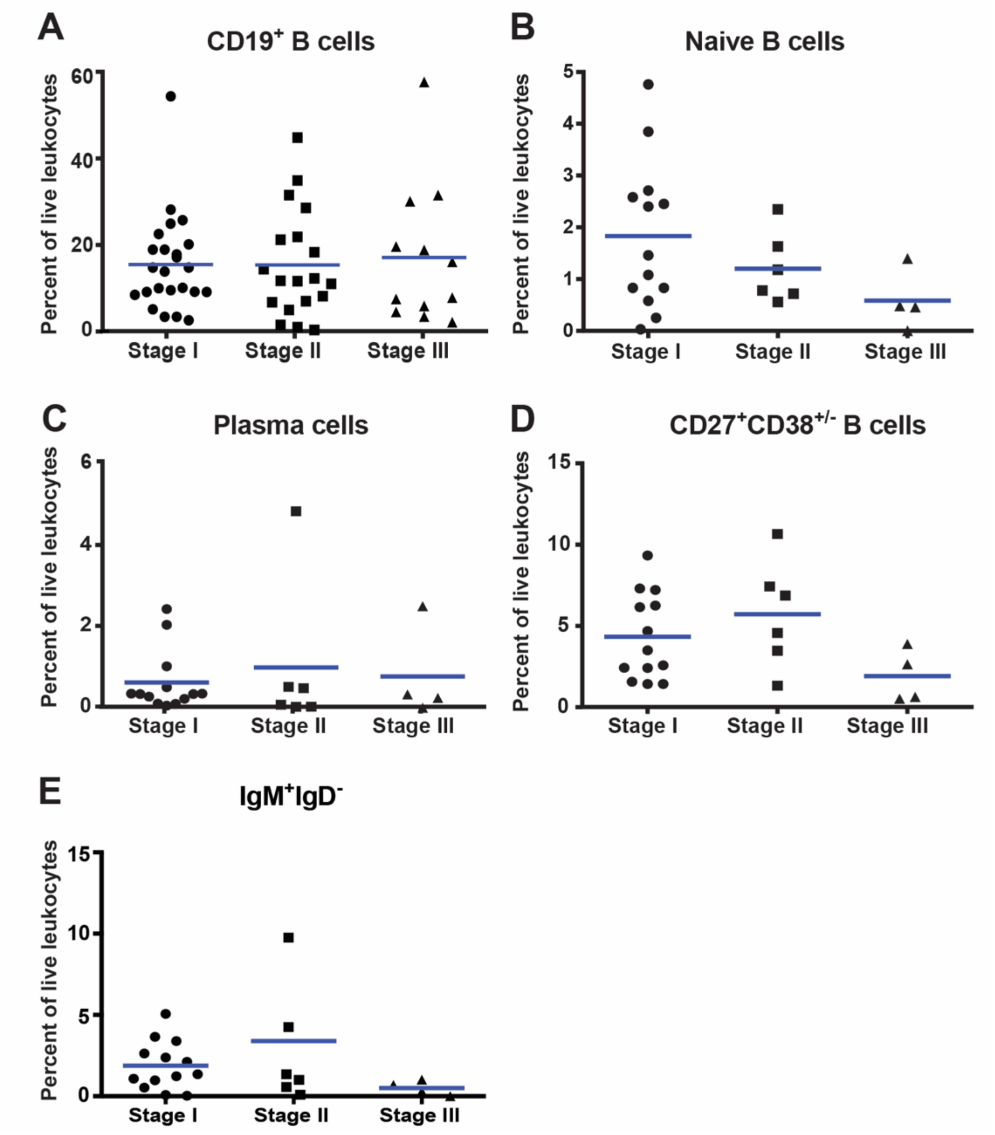

Supplementary Figure 8. Percentages of B cells in different stages of NSCLC. (A-E) Comparison of percentages of $CD19^+$ B cells and sub-populations in tumor tissue of NSCLC in stage I, II or III. **(A)** Percentages of $CD19^+$ B cells, **(B)** naive B cells, **(C)** plasma cells, **(D)** $CD27^+CD38^{+/-}$ B cells, and **(E)** IgM^+IgD^- B cells. The percentages are calculated from the total number of live leukocytes ($CD45^+PI^-$), and the gating strategy is shown in Figure 4. Each symbol represents data from one patient. Blue lines indicate mean values, and Kruskal-Wallis test and Dunn's post-test were used to detect differences between group means. No significant p values were obtained.

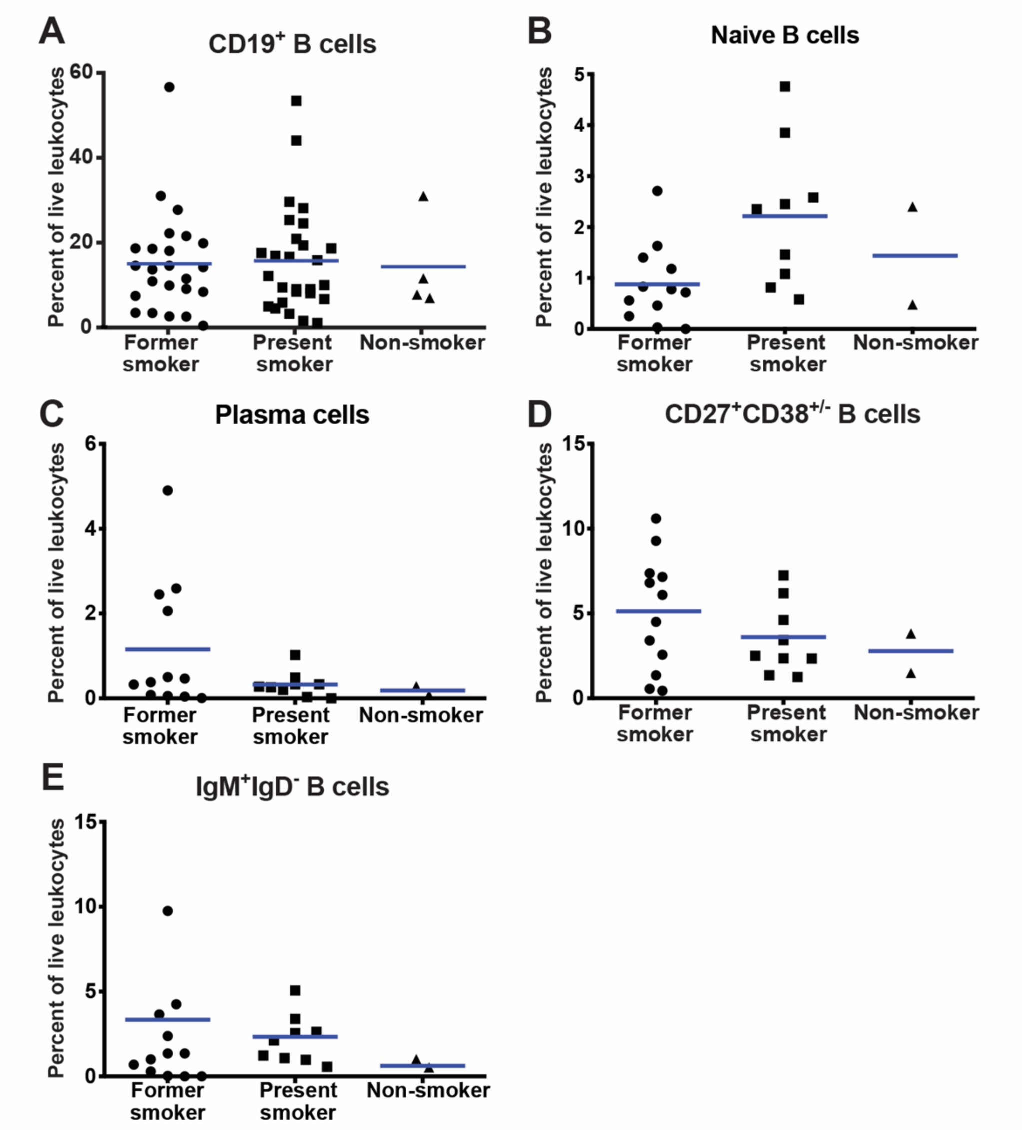

Supplementary Figure 9. Percentages of B cells in NSCLC patients with different smoking histories. (A-E) NSCLC patients were divided into 3 groups based on their smoking histories: former smoker (stopped smoking at the latest 6 months before surgery), present smoker (smoked at the time of surgery or at least 6 months prior to surgery), and non-smoker (never smoked). The proportion of B cells in tumor tissue is given as percentage of live leukocytes ($CD45^{+}PI^{-}$) and is shown for **(A)** $CD19^{+}$ B cells, **(B)** naive B cells, **(C)** plasma cells, **(D)** $CD27^{+}CD38^{+/-}$ B cells, and **(E)** $IgM^{+}IgD^{-}$ B cells. The cells were gated as indicated in Figure 4. Each symbol represents data from one patient, and blue lines indicate mean values. Kruskal-Wallis test and Dunn's post-test were used to detect differences between the groups. No significant p values were obtained.

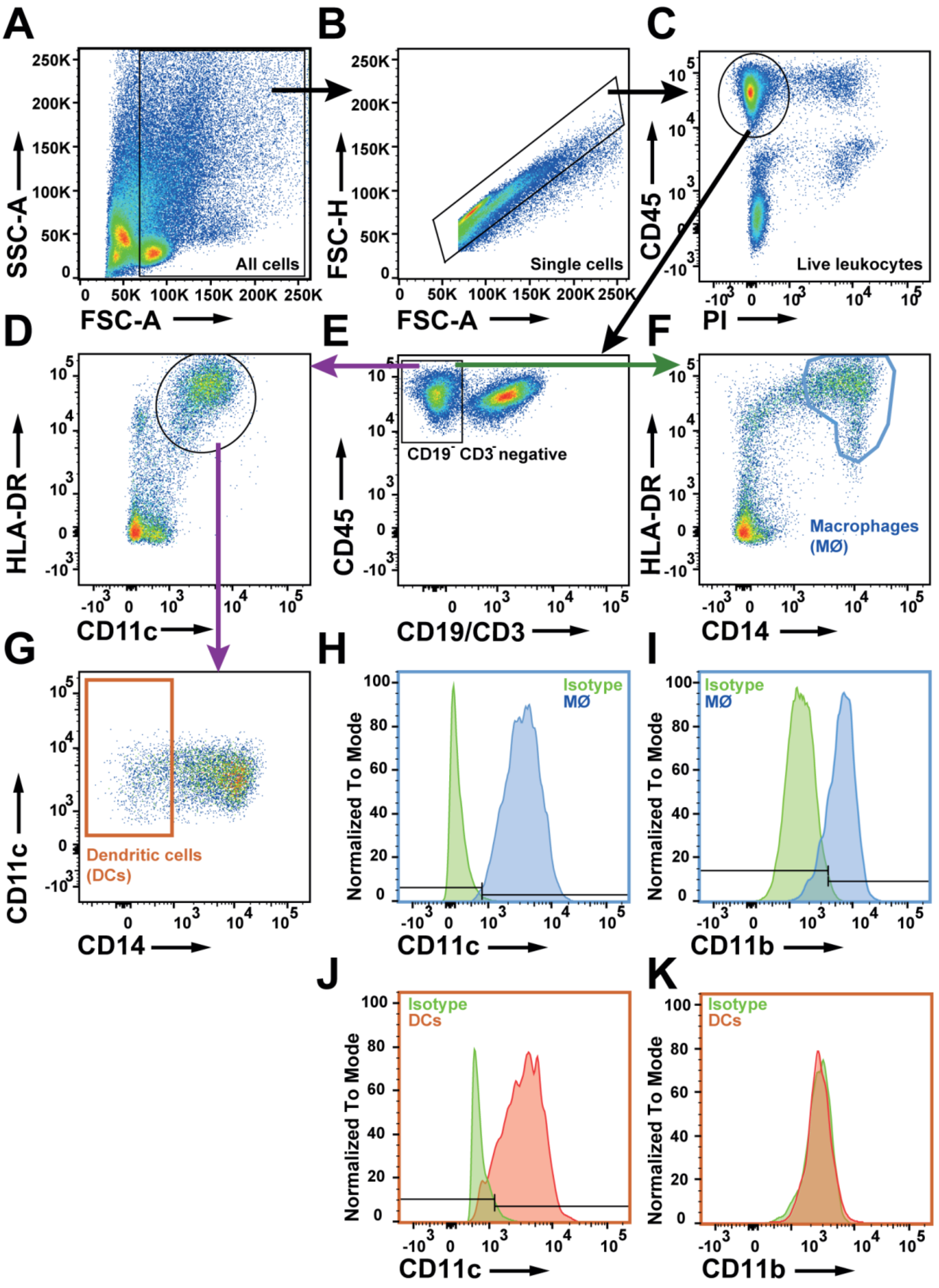

Supplementary Figure 10. Expression of CD11b and CD11c on macrophages and DCs in NSCLC tumor. The figure shows CD11b and CD11c expression in a tumor sample from one patient diagnosed with adenocarcinoma. **(A)** FSC-A and SSC-A were used to gate nucleated cells. **(B)** An FSC-A versus FSC-H plot was used to gate single cells and exclude doublets. **(C)** Live leukocytes were defined as $CD45^{+}PI^{-}$. **(D)** Cells double positive for CD11c and HLA-DR were gated among $CD19^{-}CD3^{-}$ live leukocytes. **(E)** The plot shows exclusion of $CD3^{+}$ and $CD19^{+}$ cells. **(F)** Macrophages were defined as $CD14^{+}HLA\text{-}DR^{+}$ and gated among $CD19^{-}CD3^{-}$ live leukocytes. **(G)** Myeloid DCs defined as $CD14^{-}CD11c^{+}$. **(H)** Expression of CD11c by the macrophage population (MØ) gated in panel F. **(I)** Expression of CD11b by the macrophage population (MØ) gated in panel F. **(J)** CD11c expression on the DCs gated in panel G. **(K)** Expression of CD11b by the DCs gated in panel G. Isotype-matched control antibody stainings are shown in green in the panels H-K.

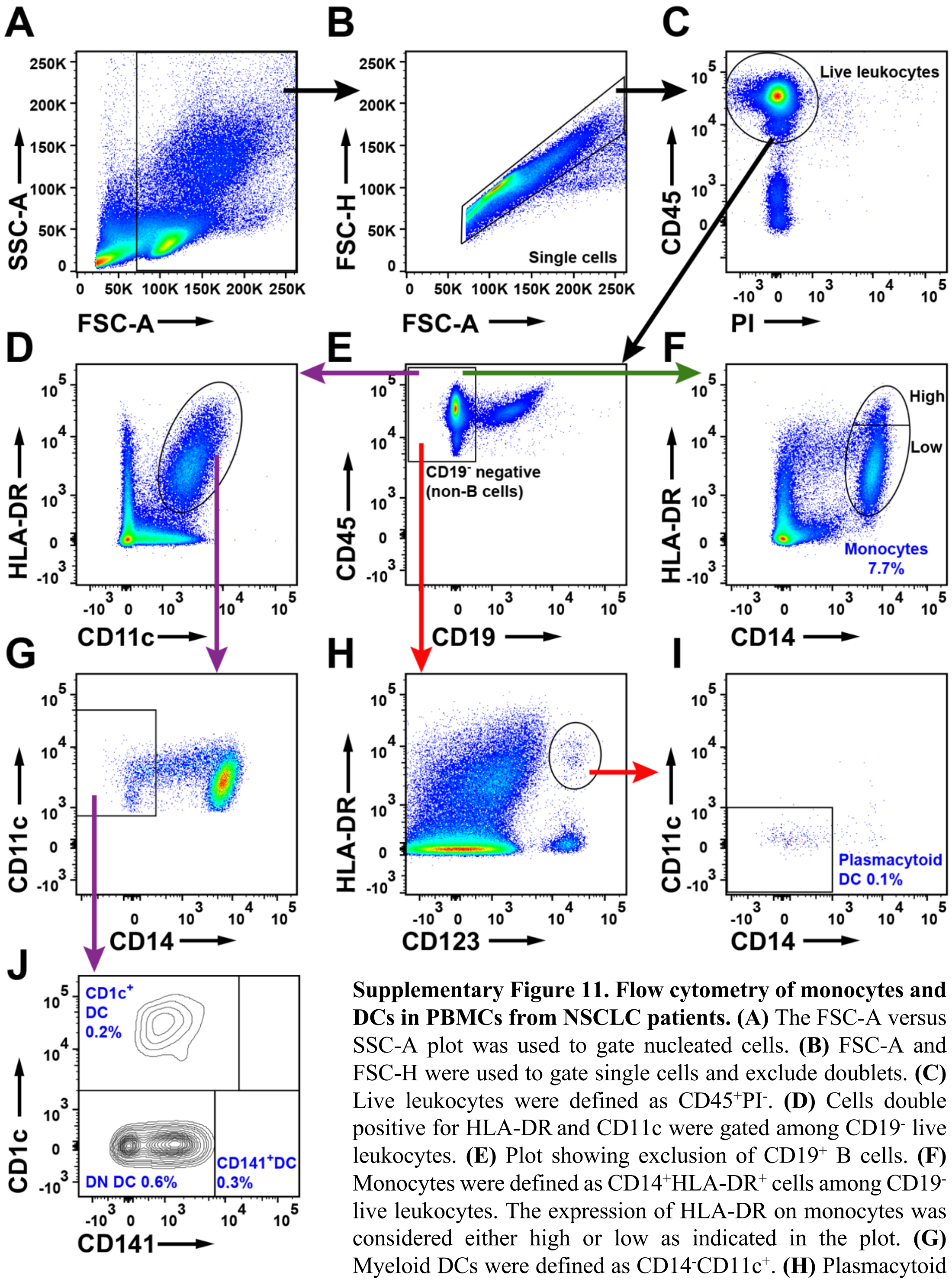

Supplementary Figure 11. Flow cytometry of monocytes and DCs in PBMCs from NSCLC patients. (A) The FSC-A versus SSC-A plot was used to gate nucleated cells. **(B)** FSC-A and FSC-H were used to gate single cells and exclude doublets. **(C)** Live leukocytes were defined as $CD45^{+}PI^{-}$. **(D)** Cells double positive for HLA-DR and CD11c were gated among $CD19^{-}$ live leukocytes. **(E)** Plot showing exclusion of $CD19^{+}$ B cells. **(F)** Monocytes were defined as $CD14^{+}HLA\text{-}DR^{+}$ cells among $CD19^{-}$ live leukocytes. The expression of HLA-DR on monocytes was considered either high or low as indicated in the plot. **(G)** Myeloid DCs were defined as $CD14^{-}CD11c^{+}$. **(H)** Plasmacytoid DCs are $CD123^{+}HLA\text{-}DR^{+}$ and also **(I)** $CD14^{-}$ and $CD11c^{-}$. **(J)** Two subsets of myeloid DCs were identified: $CD141^{+}$ DCs, and $CD1c^{+}$ DCs in addition to a population of double negative (DN) cells. The percentages of the cell populations shown in the figure were calculated from the total number of live leukocytes and represent mean values from data of 26 patients (14 adenocarcinoma, 11 squamous cell carcinoma, and 1 large cell carcinoma).

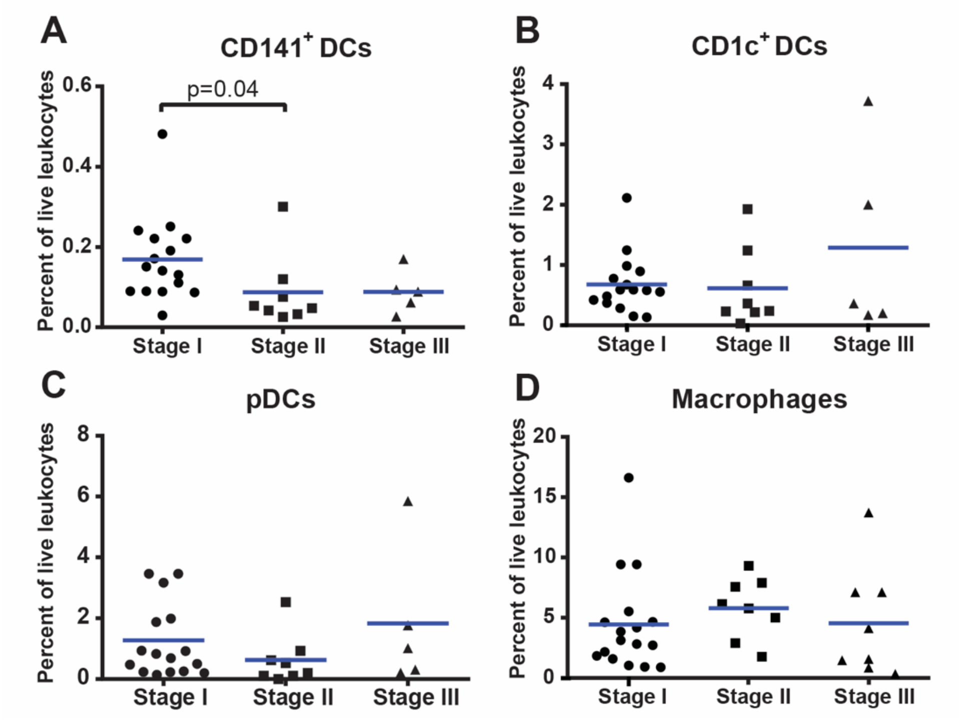

Supplementary Figure 12. Percentages of DC subsets and macrophages in different stages of NSCLC. (A-D) DC subsets and macrophages in tumor tissue are shown as percentage of live leukocytes ($CD45^{+}PI^{-}$) in stages I, II, and III of NSCLC: **(A)** $CD141^{+}$ DCs, **(B)** $CD1c^{+}$ DCs, **(C)** plasmacytoid DCs (pDCs), and **(D)** macrophages. The cells were gated as indicated in Figure 6. Each symbol represents data from one patient, and blue lines indicate mean values. Kruskal-Wallis test and Dunn's post-test were used to detect differences between stages of NSCLC.

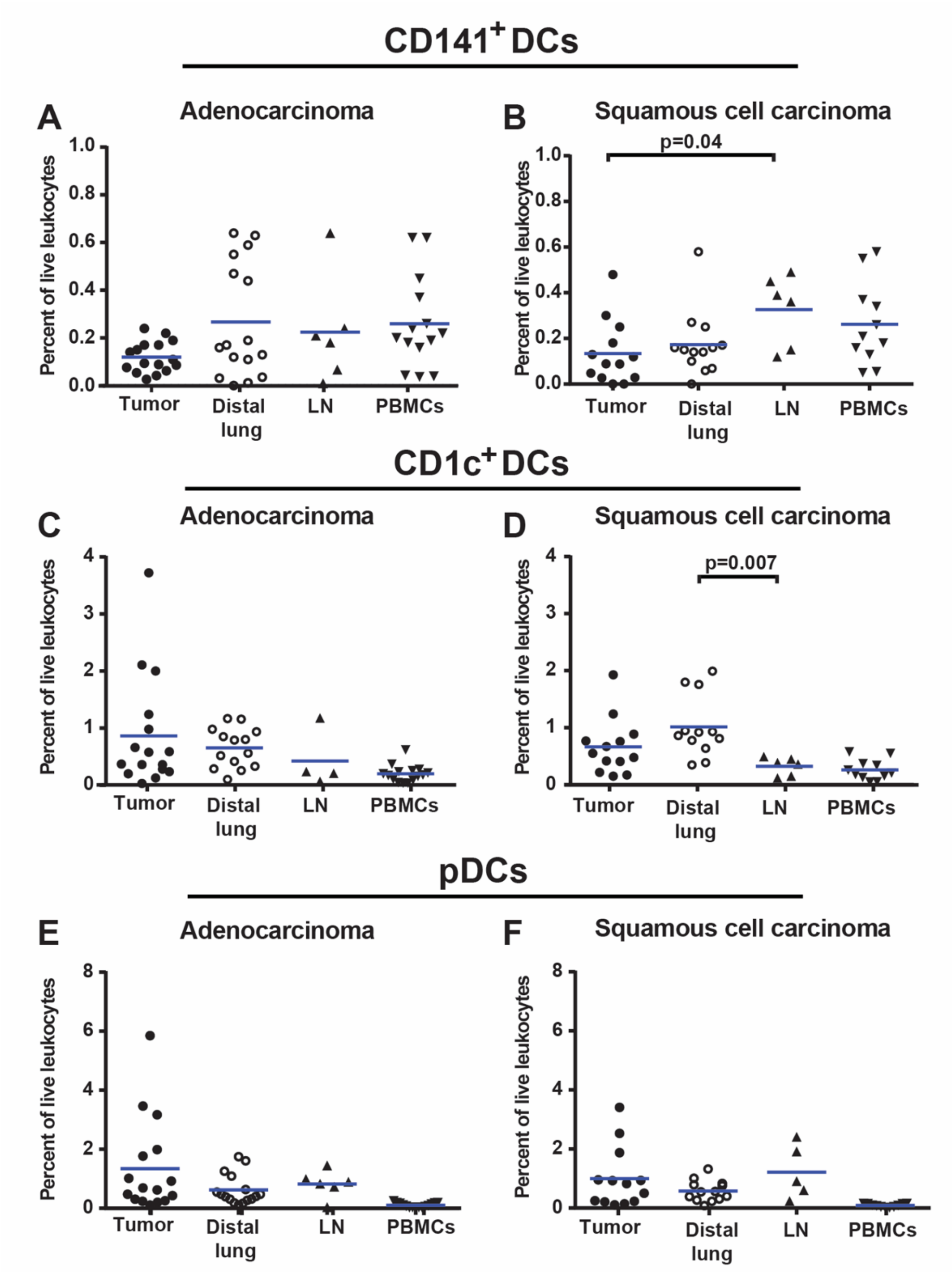

Supplementary Figure 13. Percentages of DC subsets in NSCLC adenocarcinoma and squamous cell carcinoma. **(A)** Percentages of $CD141^+$ DCs in adenocarcinoma patients. **(B)** Percentages of $CD141^+$ DCs in squamous cell carcinoma patients. **(C)** $CD1c^+$ DCs in adenocarcinoma, and **(D)** squamous cell carcinoma. **(E)** Plasmacytoid DCs (pDCs) in adenocarcinoma, and **(F)** squamous cell carcinoma. The percentages were calculated from the total number of live leukocytes ($CD45^+PI^-$), and each symbol represents data from one individual. Mean values are indicated by blue lines. The non-parametric Kruskal-Wallis test and Dunn's post-test were used to detect differences between tumor, distal lung, and lymph node (LN).

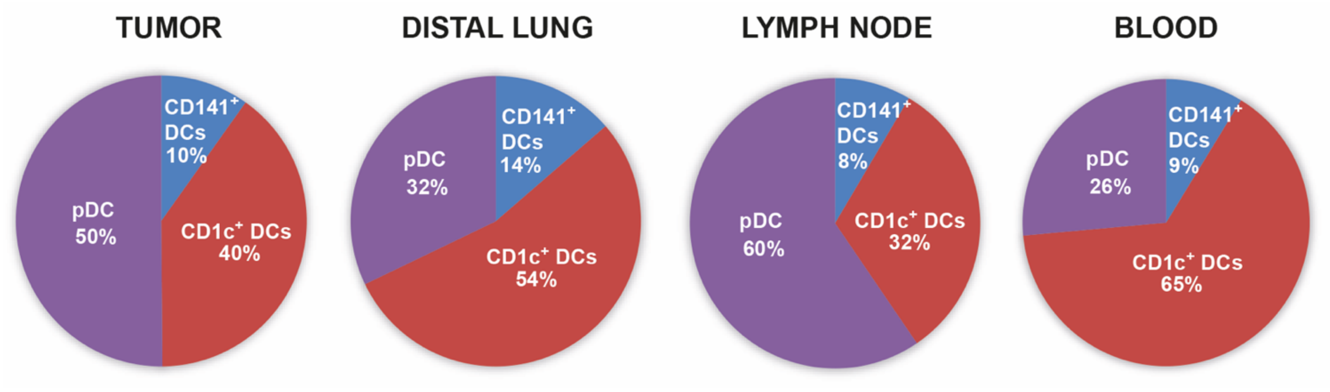

Supplementary Figure 14. Proportions of different DC populations in NSCLC. The DC subsets CD141$^+$, CD1c$^+$, and plasmacytoid DCs (pDCs) are presented as percentage of the whole DC population. Tumor tissue, distal lung (non-cancerous lung), lymph node, and blood from 29 NSCLC patients were analyzed (16 adenocarcinoma, and 13 squamous cell carcinoma). The gating strategy is shown in Figure 6, Supplementary Figure 11 and 22. Data of the individual patients included in the pie chart are shown in Supplementary Figure 15.

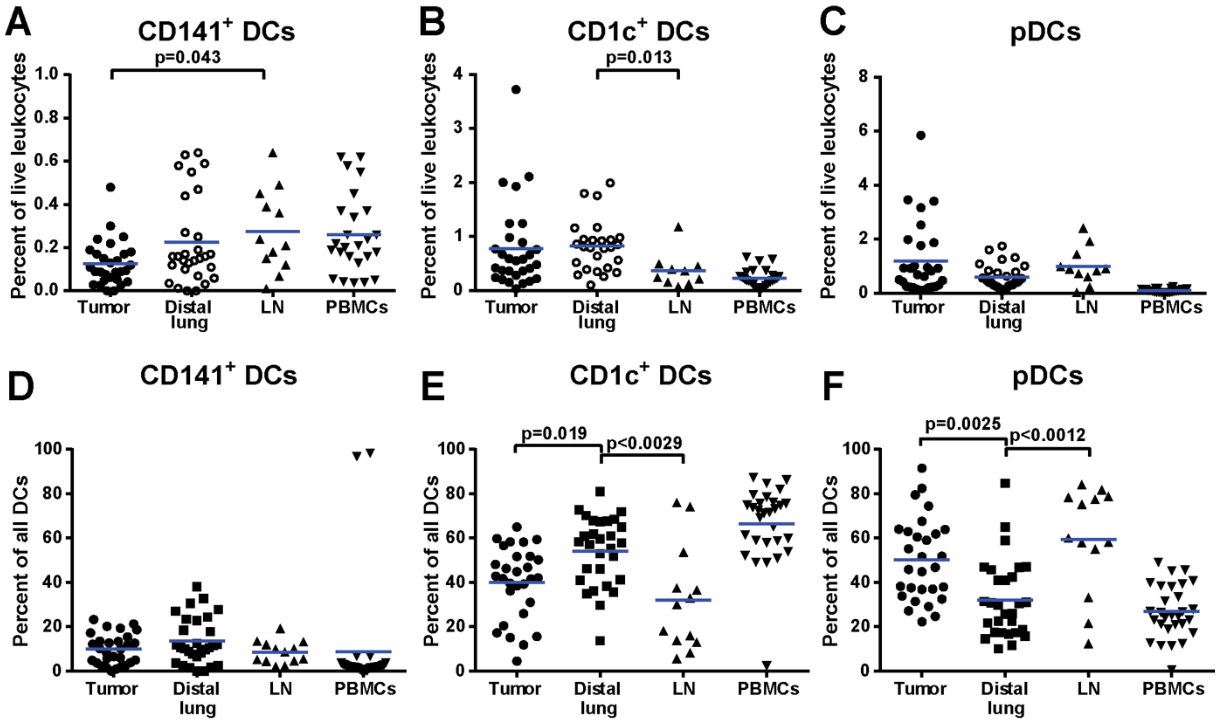

Supplementary Figure 15. Percentages of DC subsets in different tissues from NSCLC patients. **(A-F)** DC subsets were analyzed in tumor tissue, distal lung (non-cancerous lung), lymph node (LN), and PBMCs of 29 NSCLC patients (16 adenocarcinoma, and 13 squamous cell carcinoma). Subsets of DCs: **(A,D)** CD141$^+$ DCs, **(B,E)** CD1c$^+$ DCs, and **(C,F)** plasmacytoid DCs (pDCs) were identified by the gating strategies shown in Figure 6, Supplementary Figure 11 and 22. **(A-C)** The percentages of the DC subsets were calculated from the live leukocyte population (CD45$^+$PI$^-$). **(D-F)** The percentages for all three DC subsets per sample (CD141$^+$, CD1c$^+$, and pDC) calculated for panels A-C were summarized and set to 100% in order to present the proportion of each subset from the whole DC population. Each symbol in the graphs represents data from one patient, and mean values are indicated by blue lines. The non-parametric Kruskal-Wallis test and Dunn's post-test were used to detect differences between tumor, distal lung, and lymph node (LN). The results in D-F are also presented in pie charts in Supplementary Figure 14.

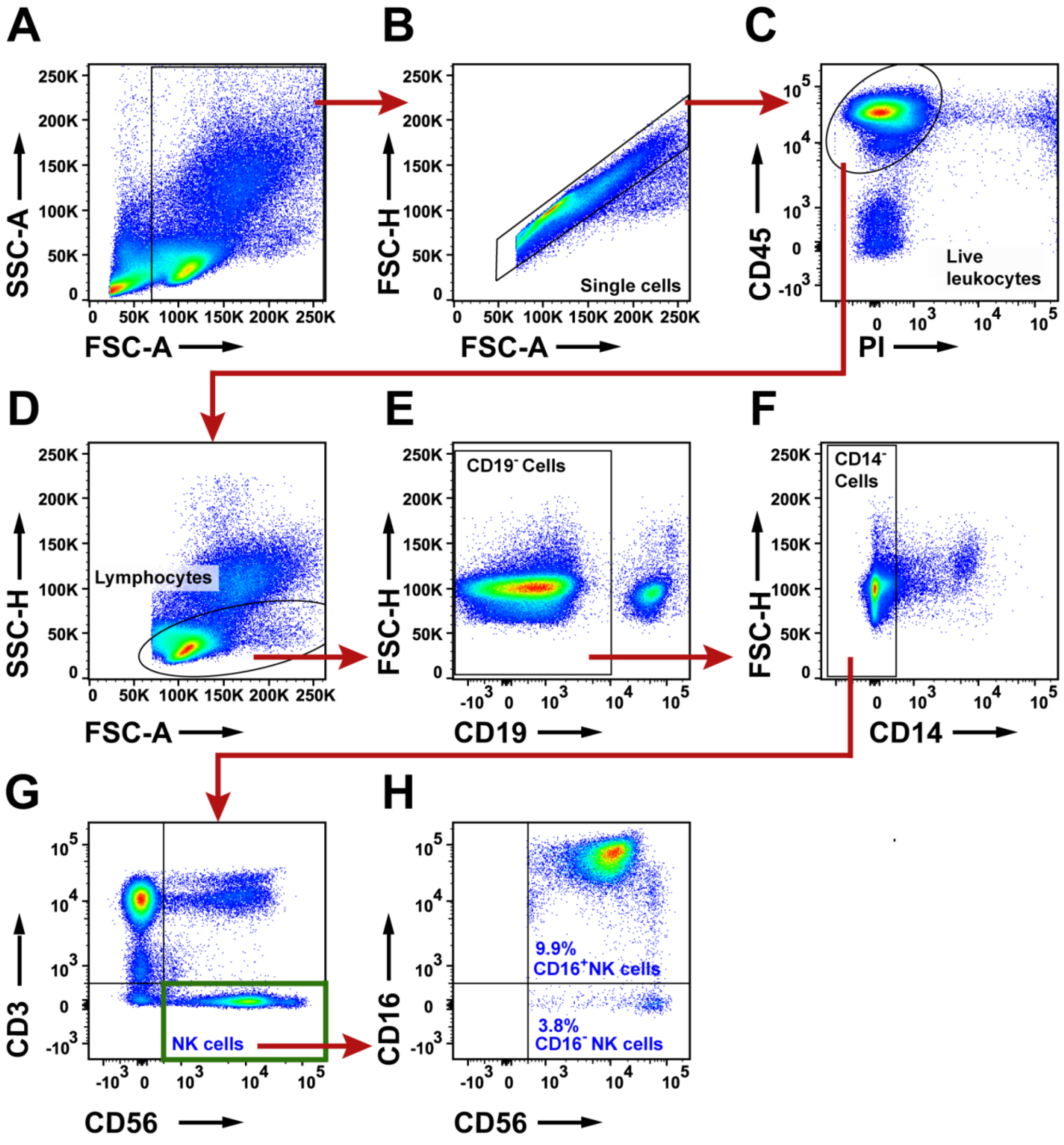

Supplementary Figure 16. Flow cytometry analysis of NK cells in PBMCs from NSCLC patients.
(A) FSC-A and SSC-A were used to gate nucleated cells. **(B)** FSC-A and FSC-H were used to exclude doublets and gate single cells. **(C)** Live leukocytes are defined as $CD45^{+}PI^{-}$ cells. **(D)** The lymphocyte gate. **(E)** $CD19^{+}$ B cells were excluded and **(F)** $CD14^{+}$ cells were excluded from the population of live leukocytes. **(G)** NK cells are defined as $CD3^{-}CD56^{+}$. **(H)** Two NK cell subsets were identified: $CD16^{+}$ and $CD16^{-}$ NK cells. The percentages shown in the figure were calculated from the population of live leukocytes, and represent mean values from data of 17 NSCLC patients (10 adenocarcinoma, 7 squamous cell carcinoma).

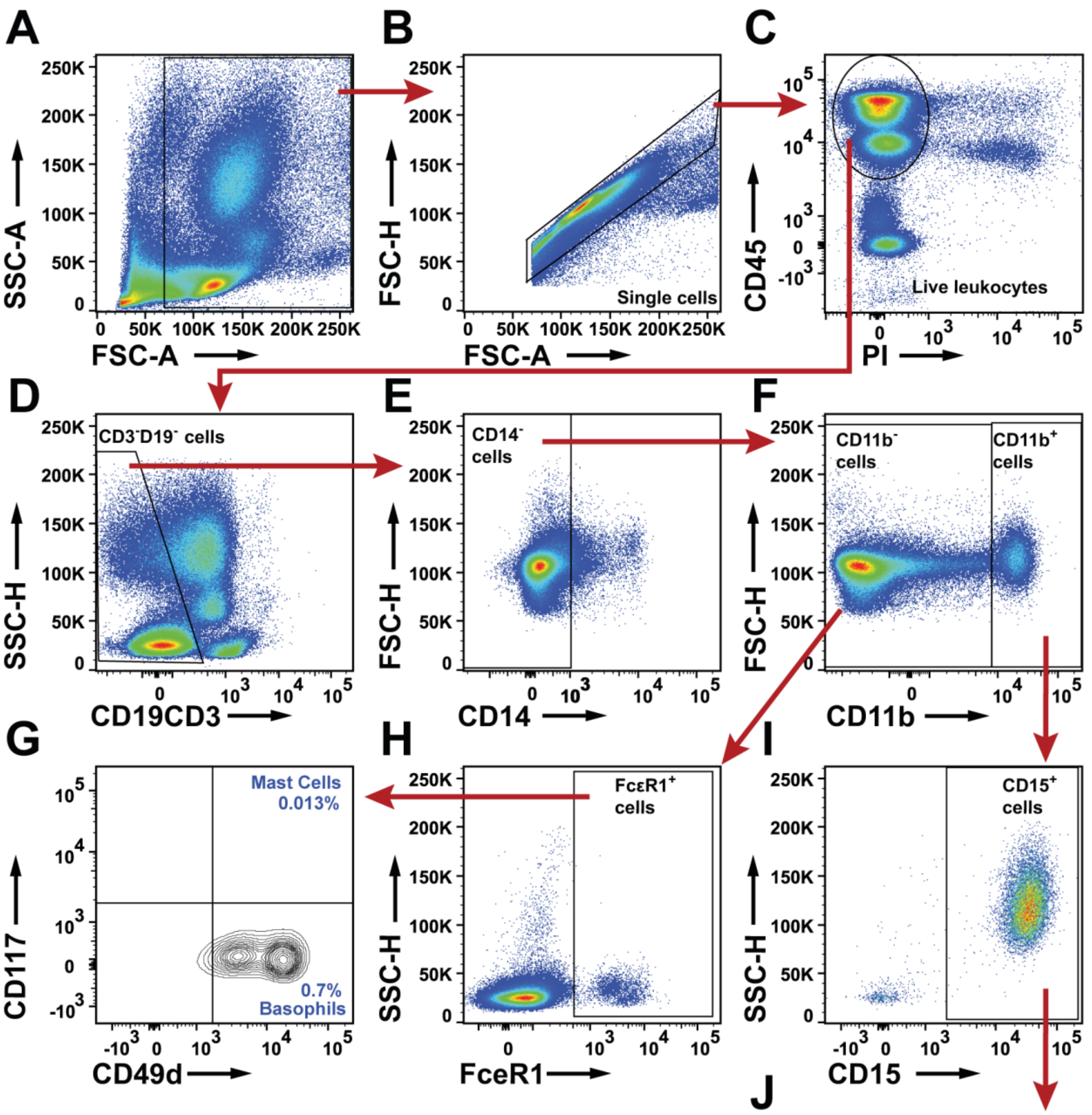

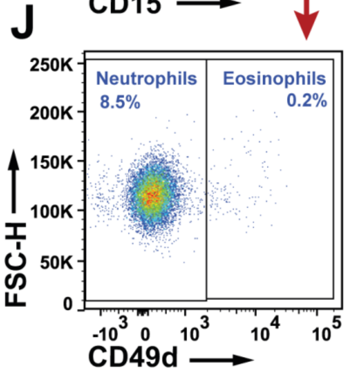

Supplementary Figure 17. Flow cytometry analysis of granulocyte populations in PBMCs from NSCLC patients. **(A)** The FSC-A versus SSC-A plot was used to gate nucleated cells and exclude debris. **(B)** The single cell gate excluding doublets. **(C)** Live leukocytes are defined as $CD45^{+}PI^{-}$. **(D)** B cells and T cells were excluded from the live leukocyte population by gating of the $CD3^{-}CD19^{-}$ cells. **(E)** $CD14^{+}$ cells were excluded. **(F)** The live leukocytes negative for CD3, CD19, and CD14 were divided into $CD11b^{-}$ cells and $CD11b^{+}$ cells. **(G-J)** The cells were further separated into mast cells and basophils ($CD11b^{-}$), and neutrophils and eosinophils ($CD11b^{+}$). **(G)** Expression of CD117 was used to divide the $CD11b^{+}Fc\varepsilon R1^{+}CD49d^{+}$ population into mast cells ($CD117^{+}$) and basophils ($CD117^{-}$). **(H)** The plot shows gating of $Fc\varepsilon R1^{+}$ cells. **(I)** In the $CD11b^{+}$ leukocyte population, neutrophils and eosinophils are defined as $CD15^{+}$. **(J)** Neutrophils are further defined as $CD49d^{-}$ and eosinophils are defined as $CD49d^{+}$. The percentage of each granulocyte population was calculated from the total number of live leukocytes, and the indicated percentages are mean values of data from 16 NSCLC patients (9 adenocarcinoma and 7 squamous cell carcinoma).

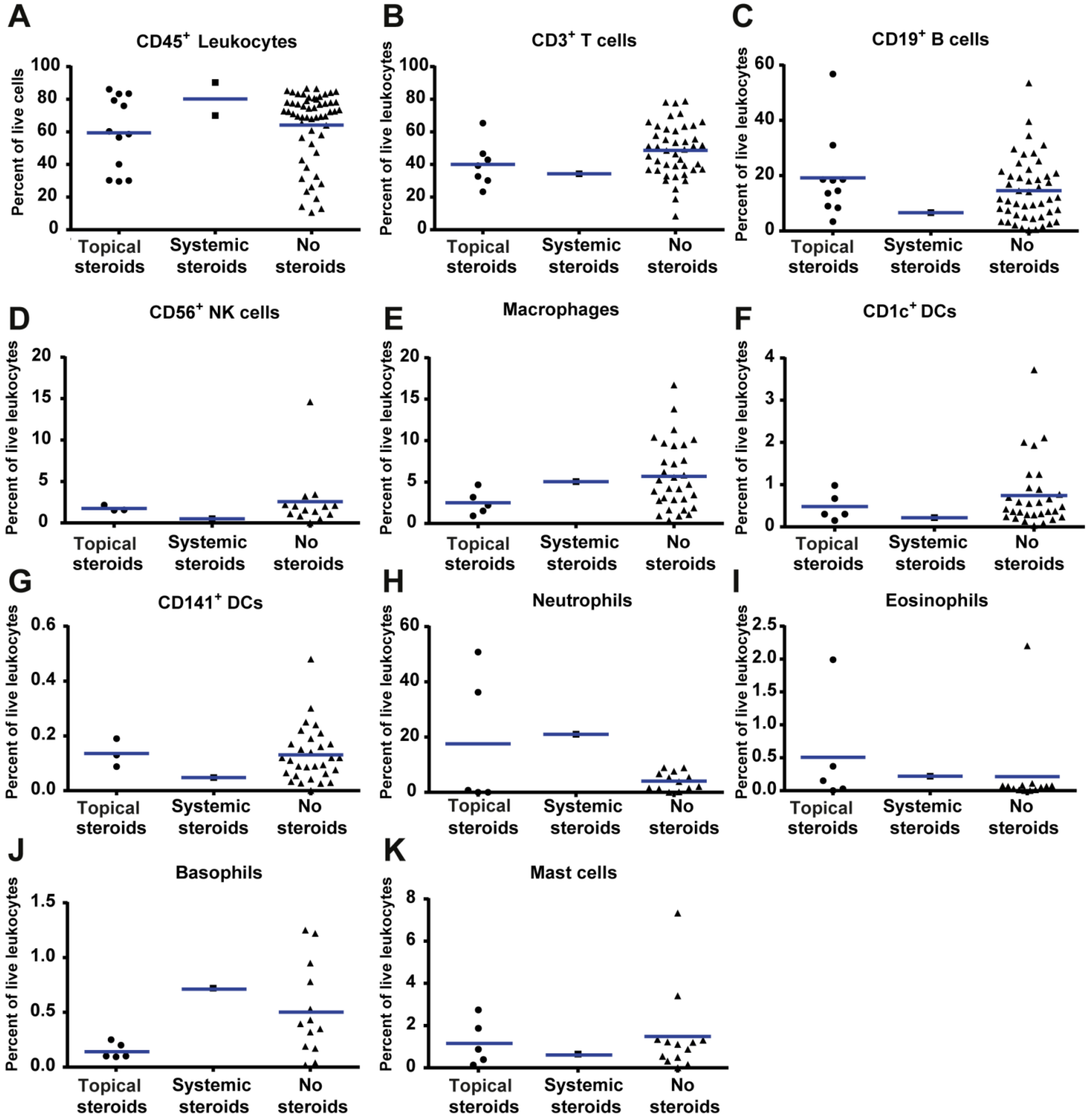

Supplementary Figure 18. Immune cell infiltrates in NSCLC tumor from patients grouped based on steroid treatment. Immune cell infiltrates were analyzed in tumors from patients treated with topical steroids, patients treated with systemic steroids, and patients who did not use steroids. **(A)** Occurrence of $CD45^{+}$ leukocytes in NSCLC tumor as percentage of all live cells. **(B-K)** Occurrence in NSCLC tumor of the indicated immune cells shown as percentage of the live leukocyte population. Each symbol represents data from one patient, and blue lines indicate mean values. Differences between group means were tested using Kruskal-Wallis test with Dunn's post-test (panel A) or Mann-Whitney test (panels B-K). No significant p values were observed.

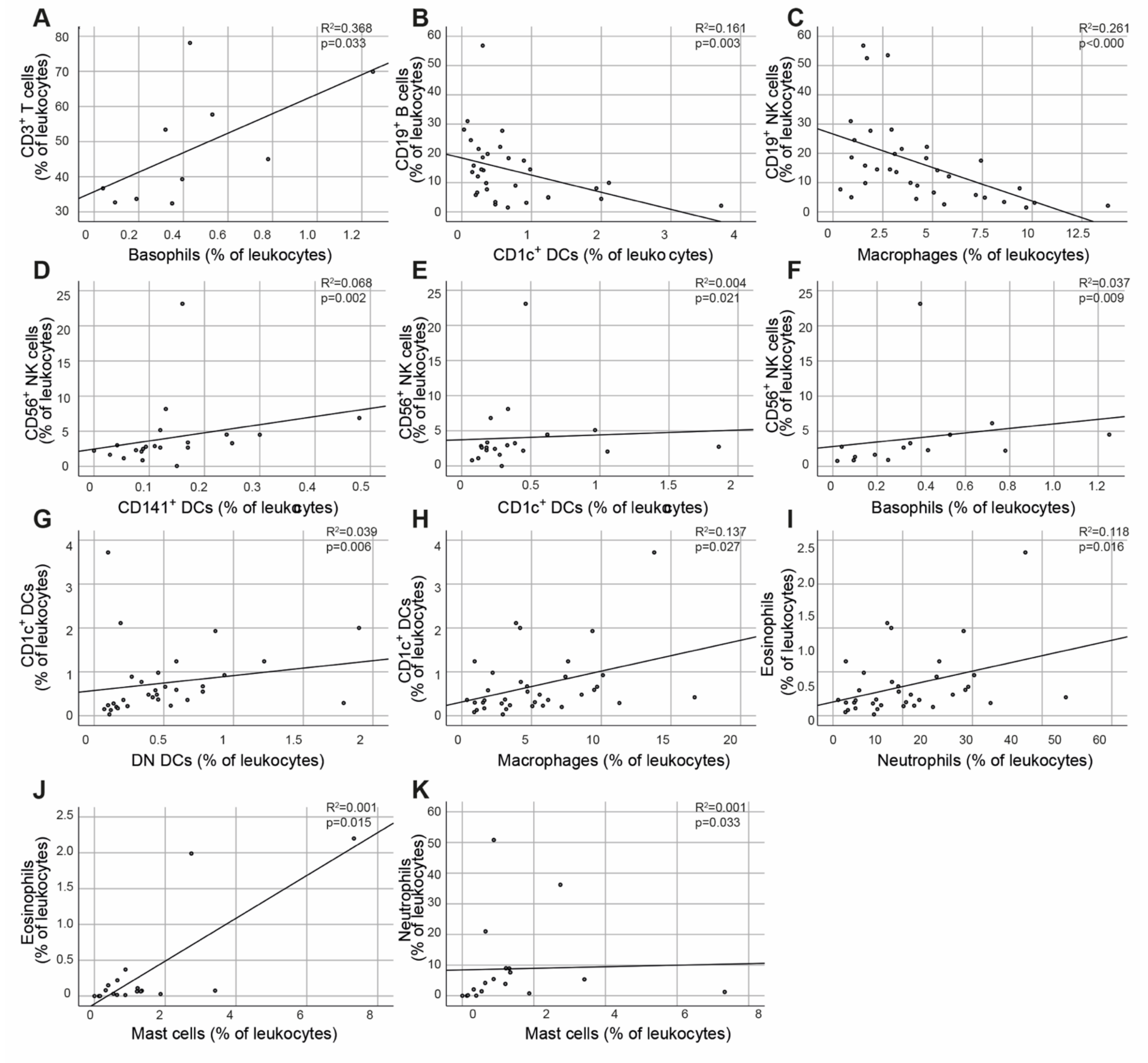

Supplementary Figure 19. Linear regression analysis of immune cell infiltrates in NSCLC tumor. Correlation of immune cell infiltrates in NSCLC tumor was analyzed by Spearman's rho and presented in Supplementary Table 7. Eleven combinations of immune cells showed significant correlation by Spearman's rho and were therefore further analyzed by regression analysis presented in the graphs **(A-K)**. Significant p values suggest an association between the indicated immune cells. The analysis was performed by IBM SPSS version 25. Percentages of the immune cells used in the analysis were calculated from the live leukocyte population. Each symbol represents data from one NSCLC patient.

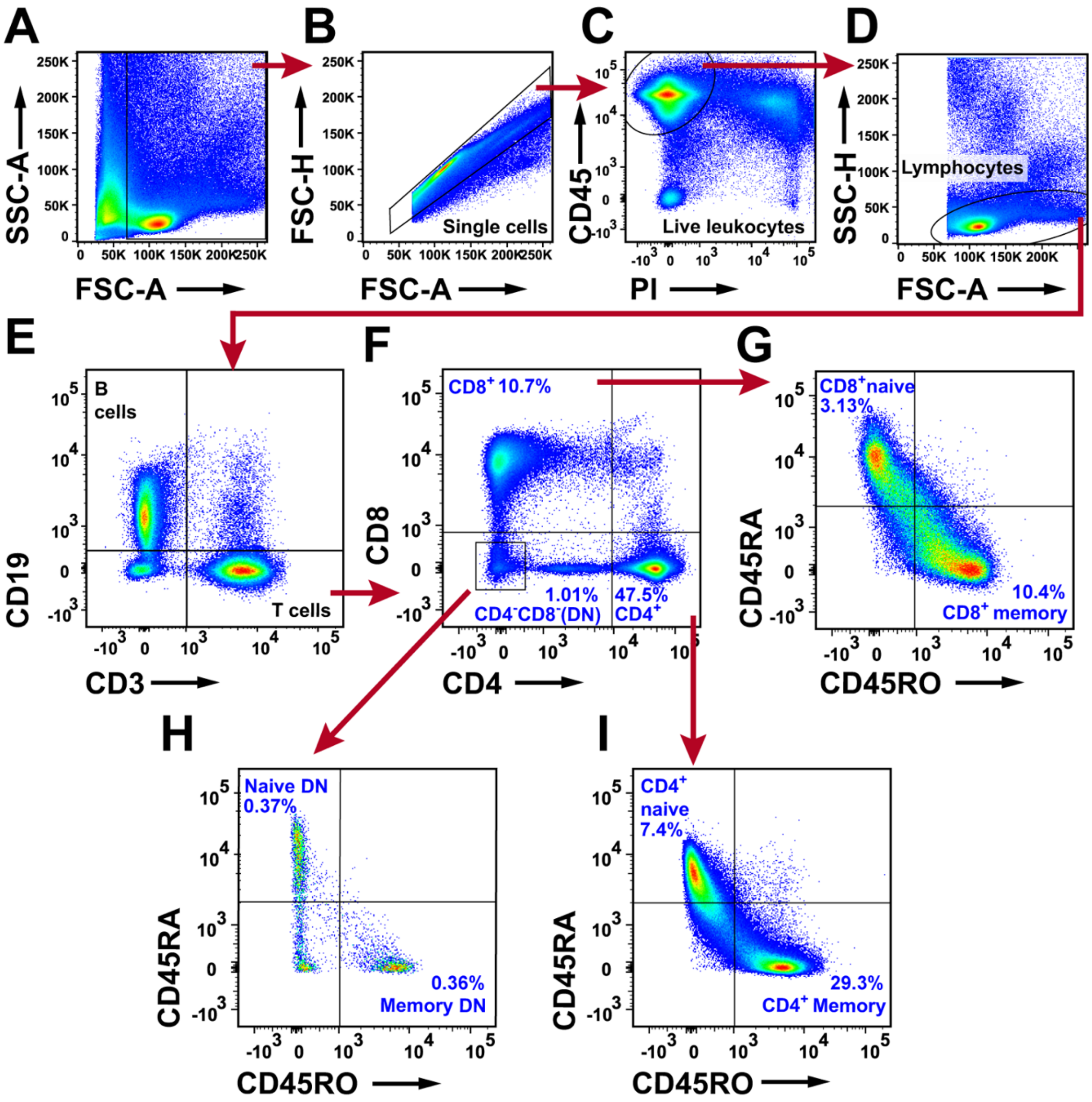

Supplementary Figure 20. Flow cytometry analysis of T cells in lymph node from NSCLC patients. (A) FSC-A and SSC-A were used to gate nucleated cells. **(B)** The FSC-A versus FSC-H plot was used to gate single cells. **(C)** Live leukocytes defined as $CD45^+PI^-$ cells. **(D)** The lymphocyte gate. **(E)** T cells are defined as the $CD19^-CD3^+$ population and were further divided in **(F)** $CD4^+$ population, $CD8^+$ population, and a $CD4^-CD8^-$ population. Each subset was examined for expression of CD45RA and CD45RO in order to separate the cells into a $CD45RA^+CD45RO^-$ naive phenotype and $CD45RA^-CD45RO^+$ memory phenotype. **(G)** Naive/memory phenotyping of $CD8^+$ T cells. **(H)** Naive/memory phenotyping of $CD4^-CD8^-$ T cells. **(I)** Naive/memory phenotyping of $CD4^+$ T cells. The percentage of each population was calculated from the total number of live leukocytes, and the indicated percentages are mean values of 26 NSCLC patients (adenocarcinoma n=15, and squamous cell carcinoma n=11). DN, $CD4^-CD8^-$ double negative T cells.

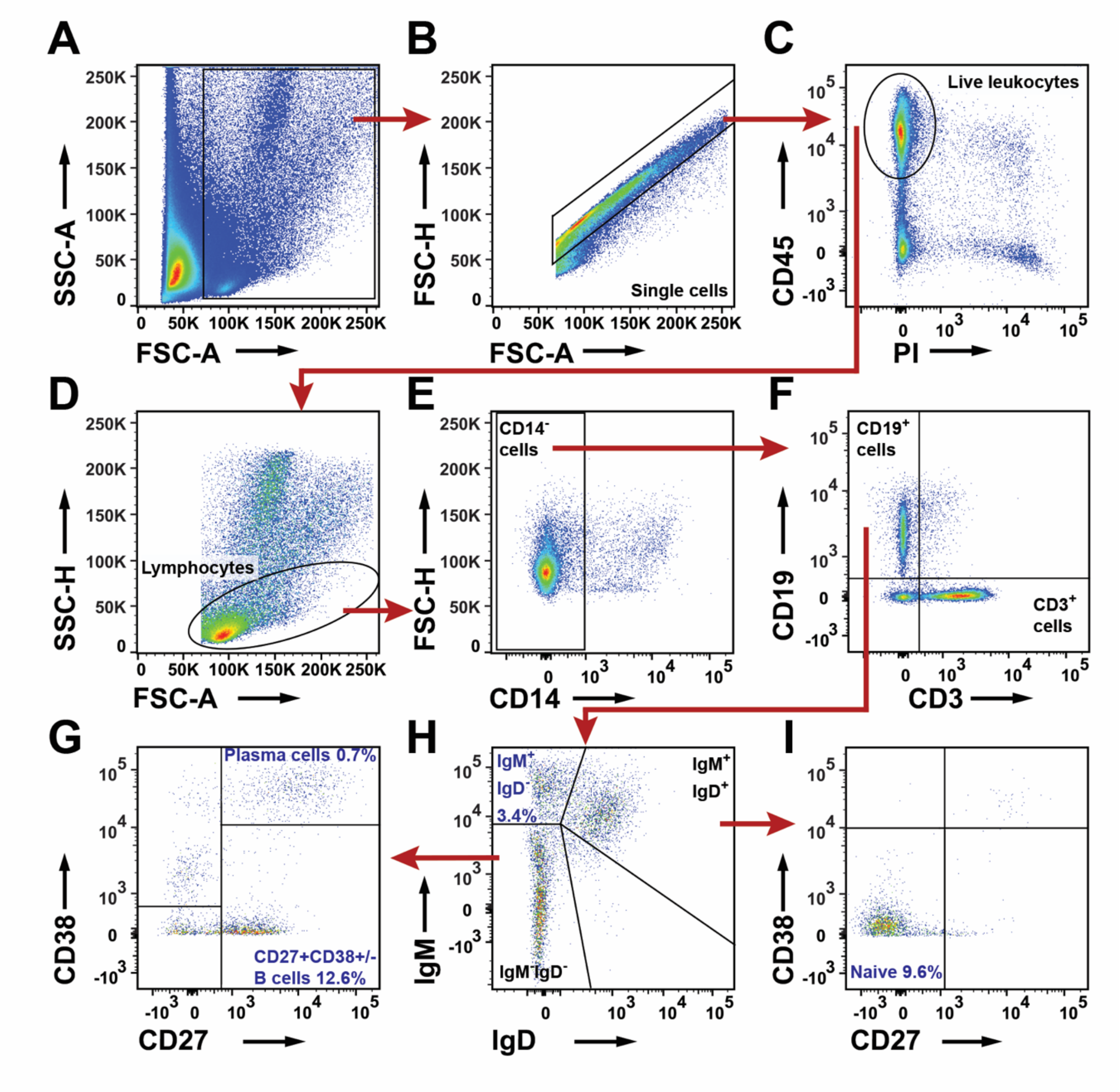

Supplementary Figure 21. Flow cytometry analysis of B cells in lymph node from NSCLC patients. **(A)** FSC-A and SSC-A were used to gate nucleated cells. **(B)** An FSC-A versus FSC-H plot was used to exclude doublets and gate single cells. **(C)** Live leukocytes are defined as $CD45^{+}PI^{-}$. **(D)** A lymphocyte gate was set in an FSC and SSC plot. **(E)** Gate excluding $CD14^{+}$ cells. **(F)** B cell gate defining the B cells as $CD19^{+}$ and $CD3^{-}$. **(G)** $IgM^{-}IgD^{-}CD19^{+}$ B cells could be separated into $CD27^{+}CD38^{+/-}$ B cells and $CD27^{+}CD38^{++}$ plasma cells. **(H)** Separation of $CD19^{+}$ B cells based on IgD and IgM expression. **(I)** Naive B cells are defined as $CD27^{-}CD38^{-/+}$. The proportions of B cell sub-populations indicated in the figure are percentages of the live leukocyte population and are mean values of 22 NSCLC patients (11 adenocarcinoma, and 11 squamous cell carcinoma).

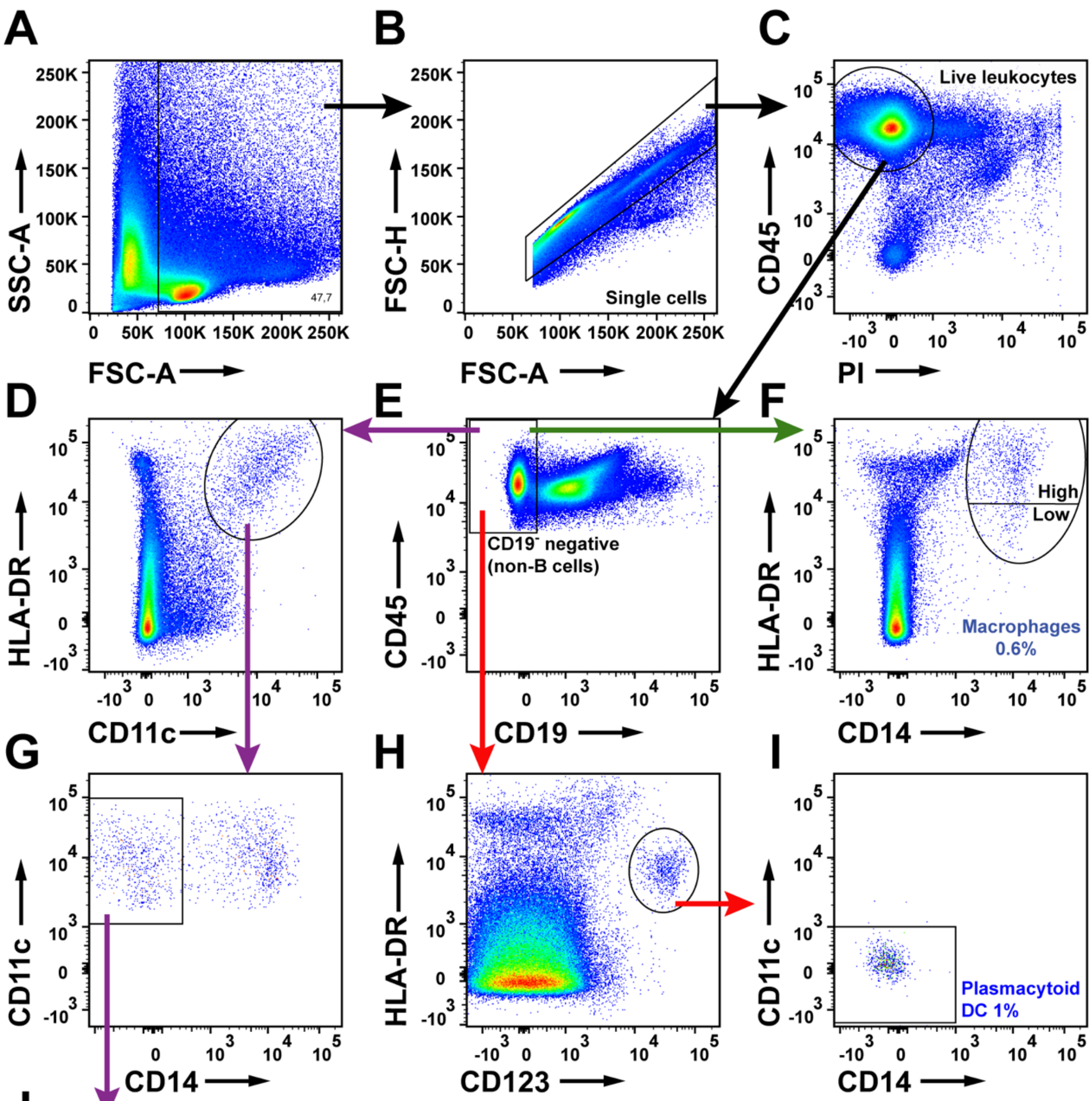

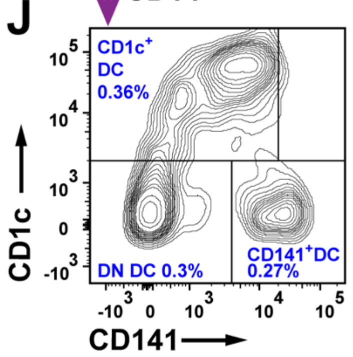

Supplementary Figure 22. Flow cytometry analysis of macrophages and DCs in lymph node from NSCLC patients. **(A)** FSC-A and SSC-A were used to gate nucleated cells. **(B)** An FSC-A versus FSC-H plot was used to exclude doublets and gate single cells. **(C)** Live leukocytes were defined as $CD45^+PI^-$. **(D)** Gate for $HLA\text{-}DR^+$ and $CD11c^+$ cells in the population of $CD19^-$ live leukocytes. **(E)** $CD19^+$ B cells were excluded from the live leukocytes. **(F)** Macrophages were defined as $CD14^+HLA\text{-}DR^+$ cells. HLA-DR expression on macrophages was considered either high or low as shown in the plot. **(G)** Myeloid DCs were defined as $CD11c^+CD14^-$. **(H)** Plasmacytoid DCs are $HLA\text{-}DR^+CD123^+$ and also **(I)** $CD11c^-$ and $CD14^-$. **(J)** Two subsets of DCs were identified: $CD141^+$ DCs, and $CD1c^+$ DCs, in addition to a population of double negative (DN) cells. The percentage of each population was calculated from the total number of live leukocytes, and the indicated percentages are mean values of data from 26 NSCLC patients (14 adenocarcinoma, 11 squamous cell carcinoma, and 1 large cell carcinoma).

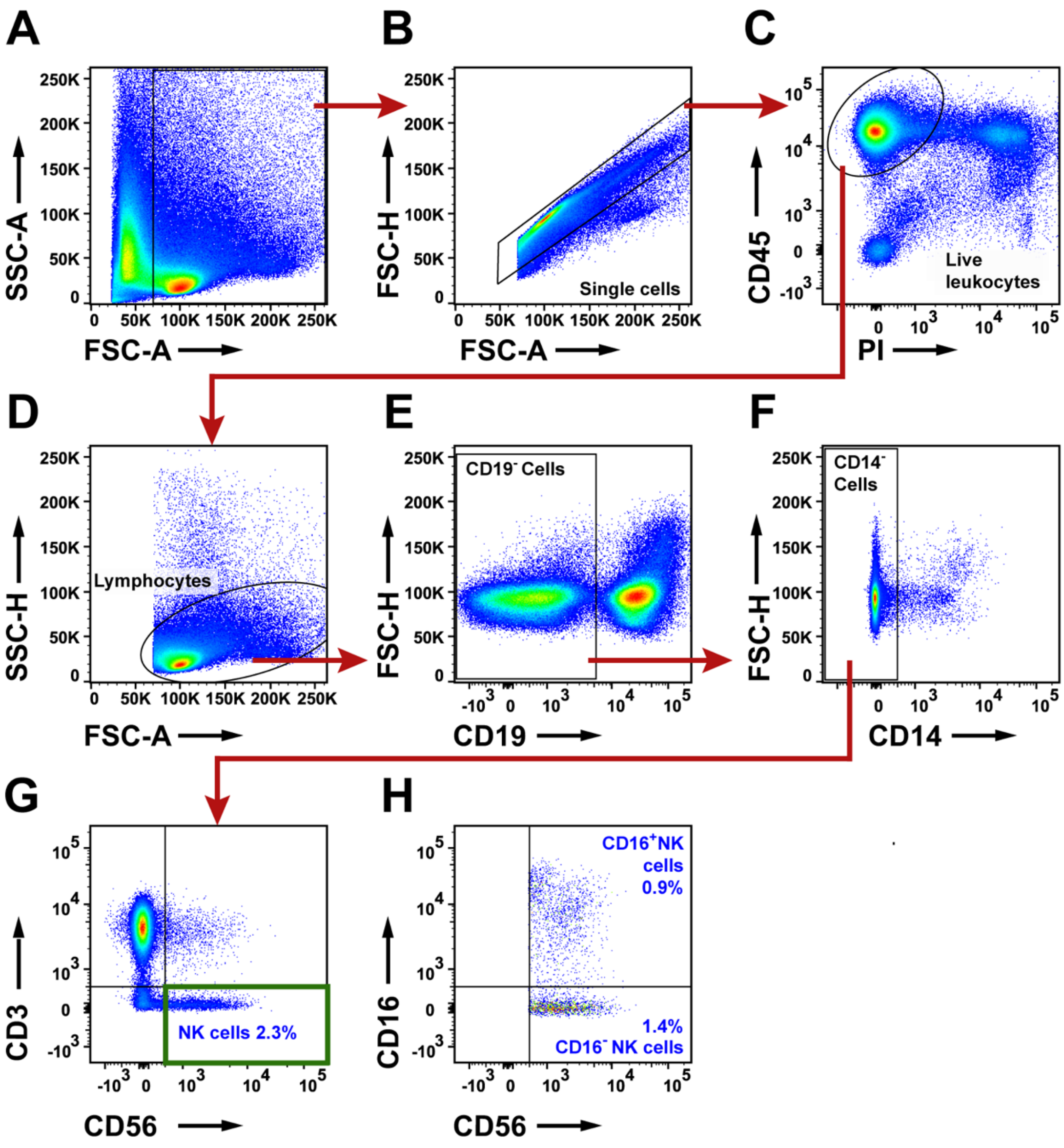

Supplementary Figure 23. Flow cytometry analysis of NK cells in lymph node from NSCLC patients. **(A)** FSC-A and SSC-A were used to exclude debris and gate nucleated cells. **(B)** An FSC-A versus FSC-H plot was used to exclude doublets and gate single cells. **(C)** Live leukocytes were defined as CD45^{+}PI^{-}. **(D)** Lymphocyte gate based on size and granularity. **(E)** CD19^{+} B cells were excluded, and **(F)** CD14^{+} macrophages were excluded from the gated lymphocytes. **(G)** NK cells are defined as CD3^{-}CD56^{+}. **(H)** Two NK cell subsets were identified: CD16^{+} and CD16^{-} NK cells. The percentages of the cell populations shown in the figure were calculated from the total number of live leukocytes and represent mean values from data of 17 NSCLC patients (10 adenocarcinoma, 7 squamous cell carcinoma).

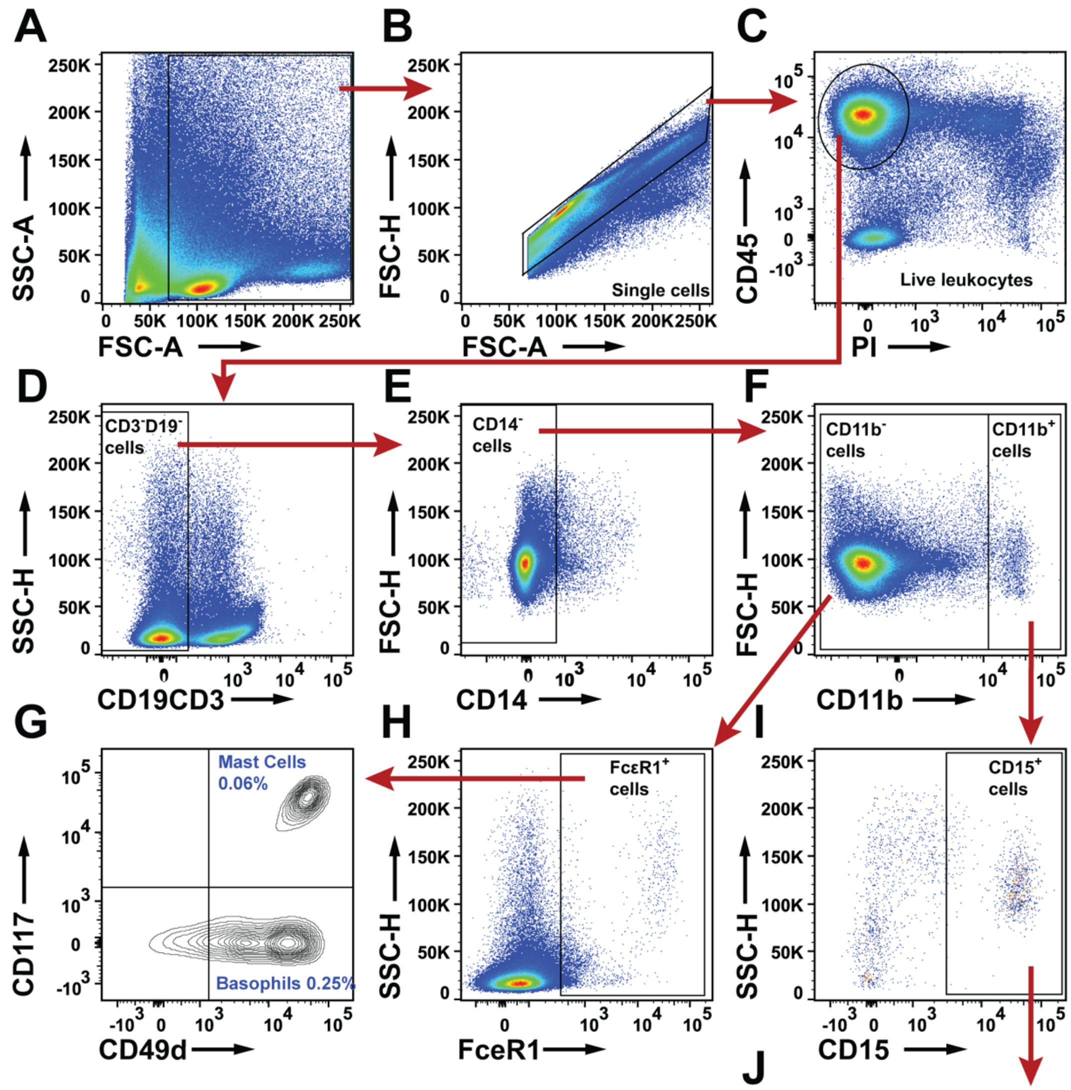

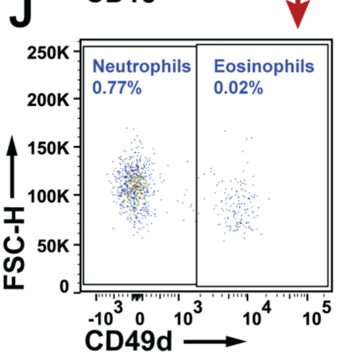

Supplementary Figure 24. Flow cytometry analysis of granulocyte populations in lymph node from NSCLC patients. **(A)** FSC-A and SSC-A were used to exclude debris and gate nucleated cells. **(B)** An FSC-A versus FSC-H plot was used to gate single cells and exclude doublets. **(C)** Live leukocytes are defined as $CD45^+PI^-$. **(D)** $CD3^+$ T cells and $CD19^+$ B cells were excluded. **(E)** $CD14^+$ macrophages were excluded. **(F)** CD11b was used to separate granulocytes: $CD11b^-$ cells include mast cells and basophils, whereas $CD11b^+$ cells include neutrophils and eosinophils. **(G)** In the population of $CD11b^-Fc\varepsilon R1^+CD49d^+$ cells, basophils were separated from mast cells by use of CD117. **(H)** Mast cells and basophils are both defined as $Fc\varepsilon R1^+$ cells. **(I)** Neutrophils and eosinophils are $CD11b^+CD15^+$. **(J)** Neutrophils are $CD49^-$ and eosinophils $CD49^+$. The percentages indicated for the granulocyte populations were calculated from the total number of live leukocytes and are mean values of 16 NSCLC patients (9 adenocarcinoma and 7 squamous cell carcinoma).

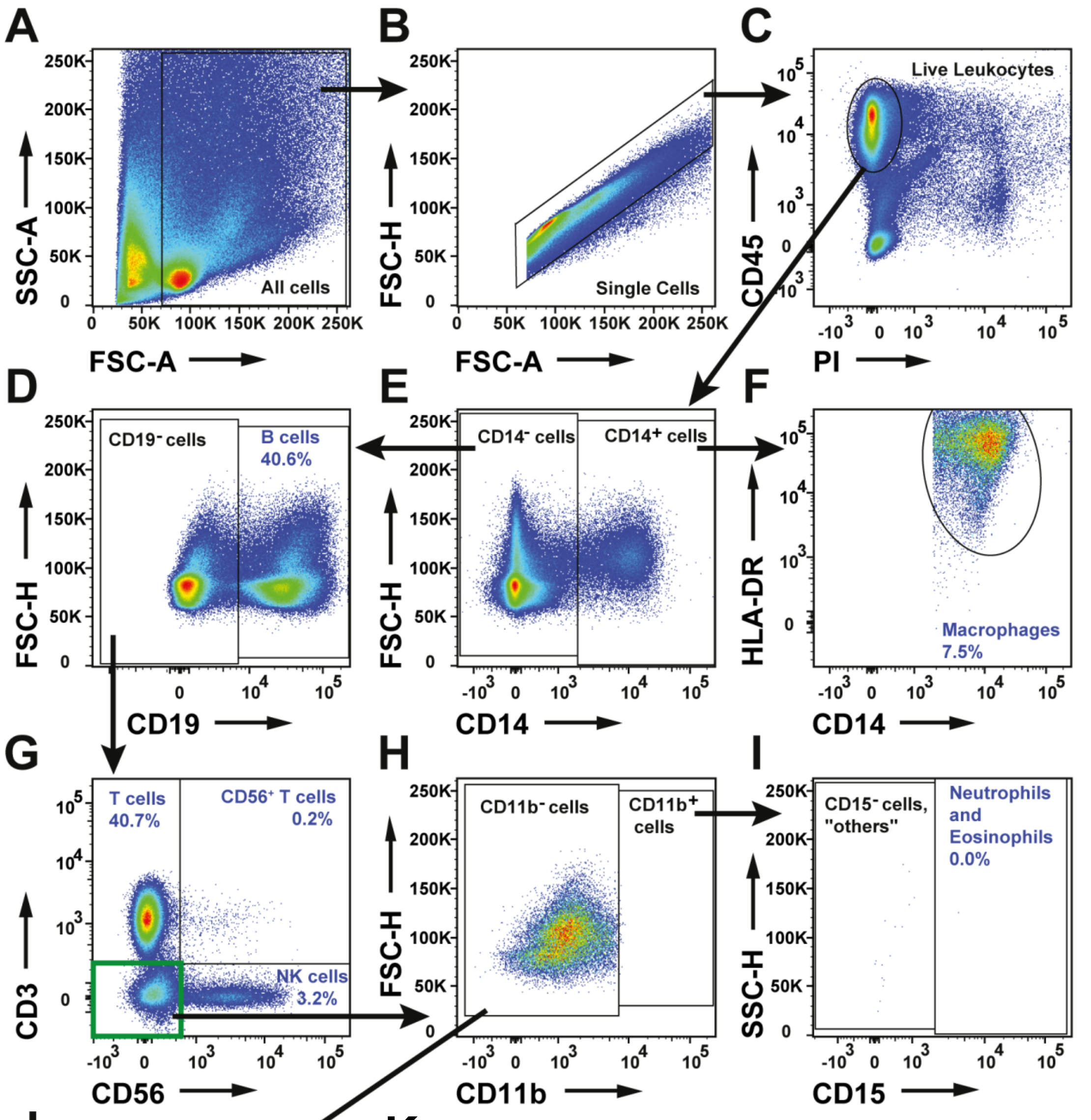

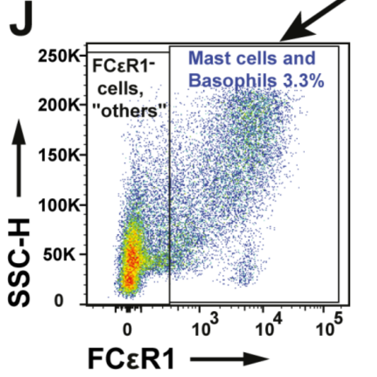

K

Cell type	Tumor	Distal lung	PBMCs
Live Leukocytes	100%	100%	100%
B cells	40.6%	19.7%	25.3%
Macrophages/Monocytes	7.5%	21.8%	20.2%
Mast cells & Basophils	3.3%	4.5%	0.9%
Neutrophils & Eosinophils	0.0%	0.0%	0.0%
NK cells	3.2%	17.2%	9.8%
$CD56^+$ T cells	0.2%	0.3%	0.3%
T cells	40.7%	27.8%	40.8%
**Total percentage of defined populations:**	**95.5%**	**91.3%**	**97.3%**
"Other"	4.5%	8.7%	2.7%

Supplementary Figure 25. Flow cytometry analysis of "Other" population in NSCLC squamous cell carcinoma. (A) The FSC-A versus SSC-A plot was used to gate nucleated cells. **(B)** FSC-A and FSC-H were used to gate single cells and exclude doublets. **(C)** Live leukocytes were defined as $CD45^+PI^-$. **(D)** $CD14^-$ leukocytes were examined for the expression of CD19. **(E)** Live leukocytes were separated into $CD14^-$ and $CD14^+$ populations. **(F)** Macrophages were defined as $CD14^+HLA\text{-}DR^+$ cells. **(G)** CD3 and CD56 were used to define T cells ($CD3^+CD56^-$), $CD56^+$ T cells ($CD3^+CD56^+$), and NK cells ($CD3^-CD56^+$). **(H)** The $CD3^-CD56^-$ population was separated based on CD11b expression. **(I)** The $CD11b^+$ population which expressed CD15 was defined as neutrophils and eosinophils. **(J)** The $CD11b^-$ cells which expressed FcεR1, were defined as mast cells and basophils. **(K)** Table presenting the proportions of immune cells as percentage of the live leukocyte population in a squamous cell carcinoma patient.

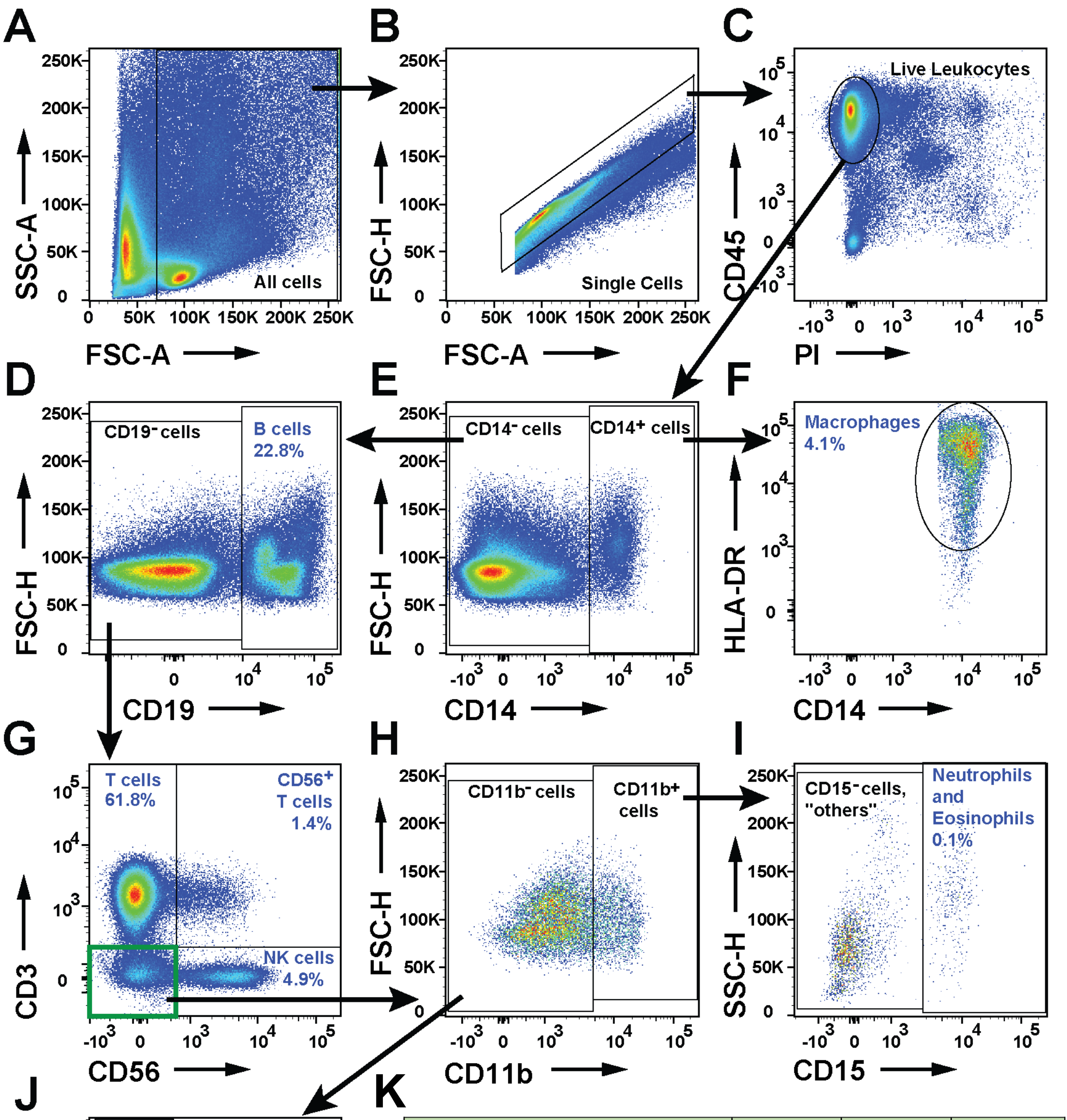

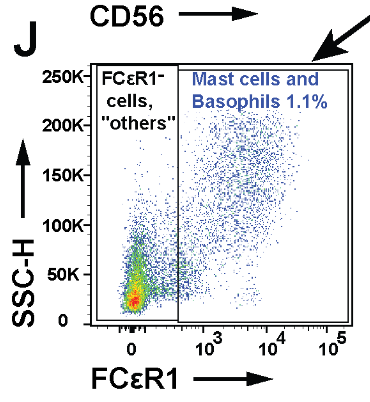

K

Cell type	Tumor	Distal lung	PBMCs
**Live Leukocytes**	100%	100%	100%
**B cells**	22.8%	20.4%	7.4%
**Macrophages/Monocytes**	4.1%	16.9%	11.1%
**Mast cells & Basophils**	1.1%	6.2%	0.5%
**Neutrophils & Eosinophils**	0.1%	0.3%	0.6%
**NK cells**	4.9%	8.2%	12.3%
**CD56^{+} T cells**	1.4%	4.4%	10.0%
**T cells**	61.8%	39.1%	55.2%
**Total percentage of defined populations:**	**96.2%**	**95.5%**	**97.1%**
**"Other"**	3.8%	4.5%	2.9%

Supplementary Figure 26. Flow cytometry analysis of "Other" population in NSCLC adenocarcinoma. **(A)** The FSC-A versus SSC-A plot was used to gate nucleated cells. **(B)** FSC-A and FSC-H were used to gate single cells and exclude doublets. **(C)** Live leukocytes were defined as CD45^{+}PI^{-}. **(D)** CD14^{-} leukocytes were examined for expression of CD19. **(E)** Live leukocytes were separated into CD14^{-} and CD14^{+} populations. **(F)** Macrophages were defined as CD14^{+}HLA-DR^{+} cells. **(G)** CD3 and CD56 were used to define T cells (CD3^{+}CD56^{-}), CD56^{+} T cells (CD3^{+}CD56^{+}), and NK cells (CD3^{-}CD56^{+}). **(H)** The CD3^{-}CD56^{-} population was further separated based on CD11b expression. **(I)** The CD11b^{+} population which expressed CD15 was defined as neutrophils and eosinophils. **(J)** The CD11b^{-} cells which expressed FcεR1^{+}, were defined as mast cells and basophils. **(K)** Table presenting the proportions of immune cells as percentage of the live leukocyte population in an adenocarcinoma patient.
